# Supplementary material for: Autonomous mobile robots for exploratory synthetic chemistry
Source: Nature. 2024 Nov 6;635(8040):890–7. doi: 10.1038/s41586-024-08173-7 (PMC11602721; doi:10.1038/s41586-024-08173-7)
Supplement: Supplementary file 1 — Supplementary Figs. 1–195 and Tables 1–53. This file contains general experimental notes, outcome of all automation experiments, manual characterization of all synthesized compounds, discussion of workflow safety and autonomy, crystallographic data and laboratory layout. [file 41586_2024_8173_MOESM1_ESM.docx]

**Supplementary Information for:**

**Autonomous mobile robots for exploratory synthetic chemistry**

Leverhulme Research Centre for Functional Materials Design and Materials Innovation Factory, University of Liverpool, 51 Oxford St, Liverpool L7 3NY, UK

**Table of Contents**

[1. Autonomous Synthesis Experiments 4](#_Toc177743005)

[1.1 General Notes 4](#_Toc177743006)

[1.2 Encoding Information from Decision-Maker to Chemspeed 4](#_Toc177743007)

[2. Parallel Organic Synthesis for Structural Diversity 5](#_Toc177743008)

[2.1 (Thio)urea Screening Experiments 5](#_Toc177743009)

[2.2 (Thio)urea Screening Results 6](#_Toc177743010)

[2.2.1 Screening (**6**) 6](#_Toc177743011)

[2.2.2 Screening (**7**) 9](#_Toc177743012)

[2.2.3 Screening (**8**) 12](#_Toc177743013)

[2.2.4 Screening (**9**) 15](#_Toc177743014)

[2.2.5 Screening (**10**) 18](#_Toc177743015)

[2.2.6 Screening (**11**) 21](#_Toc177743016)

[2.3 (Thio)rea Scale-up Synthesis 23](#_Toc177743017)

[2.4 (Thio)urea Scale-up Results 24](#_Toc177743018)

[2.4.1 Scale-up (**6**) 24](#_Toc177743019)

[2.4.2 Scale-up (**7**) 26](#_Toc177743020)

[2.4.3 Scale-up (**8**) 28](#_Toc177743021)

[2.4.4 Scale-up (**9**) 30](#_Toc177743022)

[2.4.5 Scale-up (**10**) 32](#_Toc177743023)

[2.5 Sonogashira Cross-Coupling 34](#_Toc177743024)

[2.6 (Thio)urea Sonogashira Diversification Results 35](#_Toc177743025)

[2.6.1 Diversification (**13**) 35](#_Toc177743026)

[2.6.2 Diversification (**14**) 38](#_Toc177743027)

[2.6.3 Diversification (**15**) 40](#_Toc177743028)

[2.6.4 Diversification (**16**) 43](#_Toc177743029)

[2.6.5 Diversification (**17**) 44](#_Toc177743030)

[2.7 CuAAc Reactions 45](#_Toc177743031)

[2.8 (Thio)urea CuAAC Diversification Results 46](#_Toc177743032)

[2.8.1 Diversification (**19**) 46](#_Toc177743033)

[2.8.2 Diversification (**20**) 49](#_Toc177743034)

[2.8.3 Diversification (**21**) 51](#_Toc177743035)

[2.8.4 Diversification (**22**) 53](#_Toc177743036)

[2.8.5 Diversification (**23**) 55](#_Toc177743037)

[3. Autonomous Discovery of Supramolecular Host-Guest Assemblies 56](#_Toc177743038)

[3.1 Screening for Discovery of Supramolecular Complexes 56](#_Toc177743039)

[3.2 Screening Results 57](#_Toc177743040)

[3.2.1 Supramolecular Screening One 57](#_Toc177743041)

[3.2.2 Supramolecular Screening Two 60](#_Toc177743042)

[3.2.3 Supramolecular Screening Three 62](#_Toc177743043)

[3.2.4 Supramolecular Screening Four 64](#_Toc177743044)

[3.2.5 Supramolecular Screening Five 66](#_Toc177743045)

[3.2.6 Supramolecular Screening Six 68](#_Toc177743046)

[3.2.7 Supramolecular Screening Seven 70](#_Toc177743047)

[3.2.8 Supramolecular Screening Eight 73](#_Toc177743048)

[3.2.9 Supramolecular Screening Nine 75](#_Toc177743049)

[3.2.10 Supramolecular Screening Ten 77](#_Toc177743050)

[3.2.11 Supramolecular Screening Eleven 80](#_Toc177743051)

[3.2.12 Supramolecular Screening Twelve 82](#_Toc177743052)

[3.2.13 Supramolecular Screening Thirteen 85](#_Toc177743053)

[3.2.14 Supramolecular Screening Fourteen 87](#_Toc177743054)

[3.2.15 Supramolecular Screening Fifteen 89](#_Toc177743055)

[3.2.16 Supramolecular Screening Sixteen 92](#_Toc177743056)

[3.2.17 Supramolecular Screening Seventeen 95](#_Toc177743057)

[3.2.18 Supramolecular Screening Eighteen 97](#_Toc177743058)

[3.3 Replication Experiments 99](#_Toc177743059)

[3.4 Replication Results 99](#_Toc177743060)

[3.4.1 Replication of Cage [Zn_4_(**24**_3_,**28**)_4_]^8+^ 99](#_Toc177743061)

[3.4.2 Replication of Helicate [Zn_2_(**24**_2_,**29**)_3_]^4+^ 107](#_Toc177743062)

[3.5 Host-Guest Binding Experiments 115](#_Toc177743063)

[3.6 Host-Guest Results 116](#_Toc177743064)

[3.6.1 Host-Guest Binding Studies of Cage [Zn_4_(**24**_3_,**28**)_4_](NTf_2_)_8_ 116](#_Toc177743065)

[3.6.2 Host-Guest Binding Studies of Helicate [Zn_2_(**24**_2_,**29**)_3_](NTf_2_)_4_ 119](#_Toc177743066)

[4. Offline Photochemical Synthesis 121](#_Toc177743067)

[4.1 General Reaction Procedure 121](#_Toc177743068)

[4.2 Photocatalysis Results 121](#_Toc177743069)

[4.2.1 General Comments 121](#_Toc177743070)

[4.2.2 Photocatalysis UPLC-MS Data 122](#_Toc177743071)

[5. Workflow Errors, Safety Considerations, and Workflow Autonomy 126](#_Toc177743072)

[5.1 Workflow Errors 126](#_Toc177743073)

[5.2 Safety Considerations 126](#_Toc177743074)

[5.3 Workflow Autonomy 128](#_Toc177743075)

[5.3.1 Use of a Single Mobile Robot 128](#_Toc177743076)

[5.3.2 Human Involvement and Autonomy 128](#_Toc177743077)

[6. Crystallographic data 132](#_Toc177743078)

[7. Laboratory layout 133](#_Toc177743079)

[8. References 135](#_Toc177743080)

# Autonomous Synthesis Experiments

## General Notes

For the parallel organic synthesis and supramolecular discovery workflows, stock solutions were prepared on the Chemspeed. The volumes of solvents required to make set concentrations from these stock solutions were inputted as a CSV file that was read by the Chemspeed program.

For the offline-photochemistry experiments, the only variation between samples was the choice of photocatalyst. On our Chemspeed platform, we used acetonitrile as the system solvent, and used single solvent aspirations for all transfers.

## Encoding Information from Decision-Maker to Chemspeed

In the CSV file used for inputting volumes of stock solutions, our decision-maker populates a specified ‘Reaction Binary’ column with either 1 or 0 from the combined pass/fail grading of the NMR and/or LC-MS measurements for each sample the previous stage.

For example, if (thio)ureas **6**, **7**, **8**, **9**, and **10** was judged as successful, while thiourea **11** was unsuccessful in screening stage (Figure 2a), the column in the CSV file would be encoded as [1,1,1,1,1,0] by our decision-maker so that only **6**-**10** are synthesized in the next scale-up stage, while **11** is not.

After the first stage of our Chemspeed program, the values of all volumetric transfers were multiplied by the values of this ‘Reaction Binary’ column. Reactions judged as ‘failed’ were read as 0 volume (and therefore not dispensed) by the Chemspeed Autosuite software, and unsuccessful reactions from the previous stage were not taken forward to the next stage, either for dispensing reagents for the relevant reaction or reformatting for analysis.

# Parallel Organic Synthesis for Structural Diversity


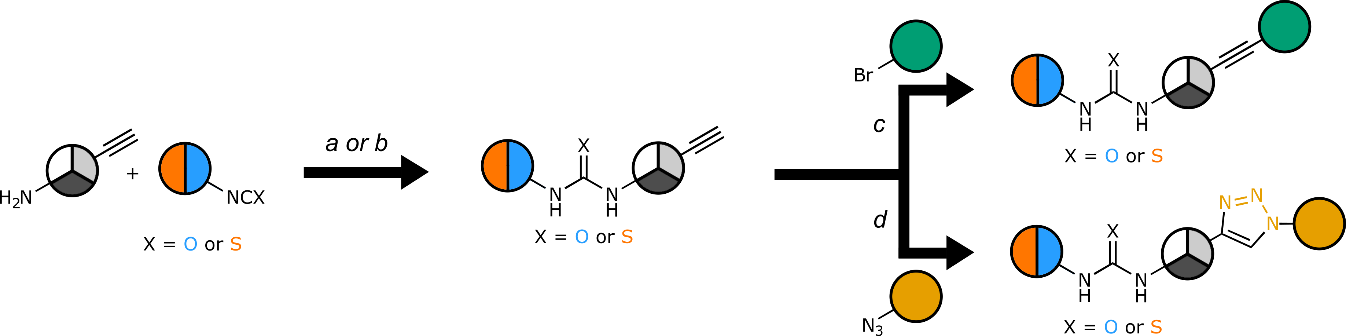


**Scheme S 1:** General overview of automated divergent medicinal chemistry synthesis route. (a) 3,5-bis(trifluoromethyl)-phenyl isocyanate (1 eq.), CH_2_Cl_2_, r.t., 12 h. (b) (a) 4-Fluorophenyl isothiocyanate(1 eq.), CH_2_Cl_2_, r.t., 12 h. (c) 2-Bromopyridine (1 eq.), K_2_CO_3_ (1.6 eq.), NaPdCl_4_ (10 mol%)_,_ cataCXium Fsulf (10 mol%), H_2_O:IPA (1:1), r.t., 14 h. (d) Zidovudine (1 eq.), CuSO_4_ (10 mol%), ascorbic acid (20 mol%)_,_ CH_2_Cl_2_:H_2_O:IPA (2:1:1), 60 °C, 14 h.

## (Thio)urea Screening Experiments

The ISynth Chemspeed platform was charged with 20 mL ISynth vials containing pre-weighed amounts of the 3 selected amines and anilines (4-ethynylaniline, prop-2-yn-1-amine, but-3-yn-1-amine) and the two iso(thio)cyanates (1-isocyanato-3,5-bis(trifluoromethyl)benzene and 1-fluoro-4-isothiocyanato-benzene), as well as stock bottles of CH_2_Cl_2_ (400 mL). CH_2_Cl_2_was transferred to each of the vials and the vials were shaken to prepare stock solutions (200 mM) of each of the reagents. Combinations of one iso(thio)cyanate and one amine/aniline reagent were prepared by transferring aliquots (2 mL each) from the stock solutions to separate 20 mL ISynth vials. The vials were shaken overnight for 12 hours. A further portion of CH_2_Cl_2_ (6 mL) was added to the reaction vials, and the vials were further shaken to ensure complete dissolution of the urea products for analysis.

Aliquots (0.7 mL) of the diluted reaction mixture was transferred to NMR tubes. A further aliquot (0.1 mL) of the reaction mixture was then transferred to LC-MS vials, and each LC-MS vial was then diluted with acetonitrile (1.9 mL). The LC-MS vial tray and the NMR rack were then transported robotically to the UPLC machine and benchtop NMR machine for analysis.

For further offline manual analysis by high-resolution mass spectrometry and high-field NMR, CH_2_Cl_2_was evaporated, and the products were further characterized on high-field NMR.

## (Thio)urea Screening Results

### Screening (6)

#### Outcome of Automation


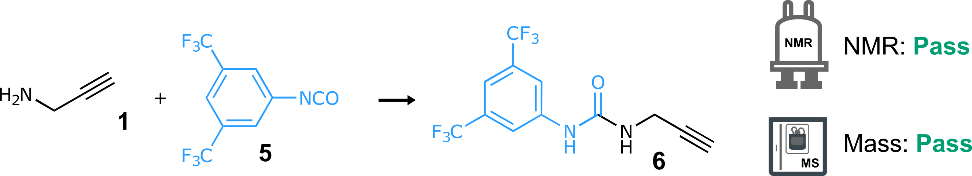


**Scheme S 2:** Synthesis of urea **6** from amine **1** and isocyanate **5**. Reaction conditions: CH_2_Cl_2_, r.t., 12 h.

| *NMR*: Pass | *MS*: Pass |
| --- | --- |
| *DTW distance*: 36.6110 | *retention time*: 2.04 min  *LC area*: 100% |
|  | *ion observed*: [**6**+H]^+^  *m/z expected*: 311.06  *m/z measured*: 310.93  *ion observed*: [**6**+CH_3_CN+H]^+^  *m/z expected*: 352.09 *m/z measured*: 352.09 |

**Table S 1:** Summary of automated decision-maker outcomes for ^1^H NMR spectroscopy and ULPC-MS spectrometry.


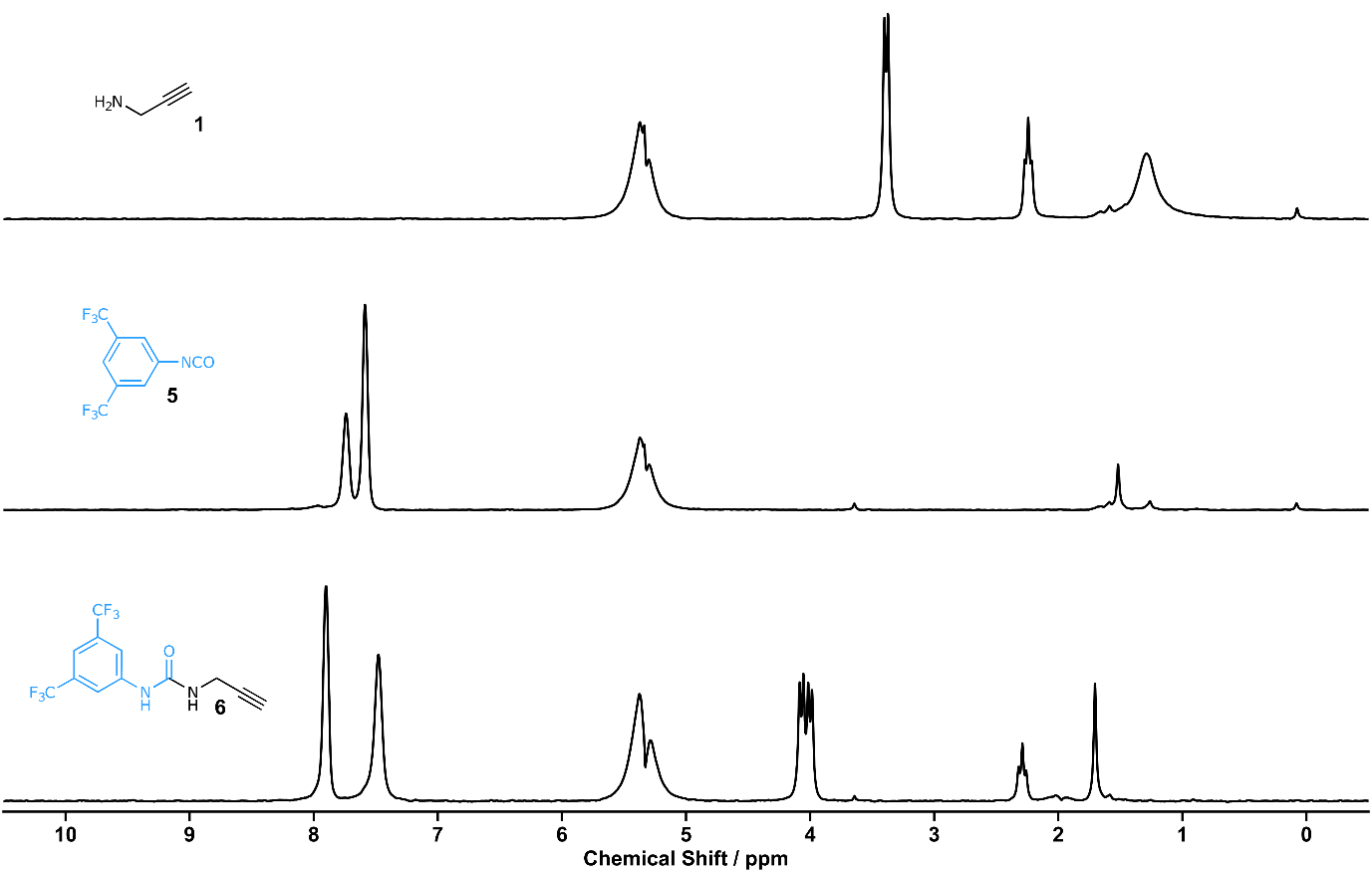


**Figure S 1:** ^1^H NMR spectrum (80 MHz, CH_2_Cl_2_) of amine **1** (top), isocyanate **5** (middle), screening **6** (bottom).


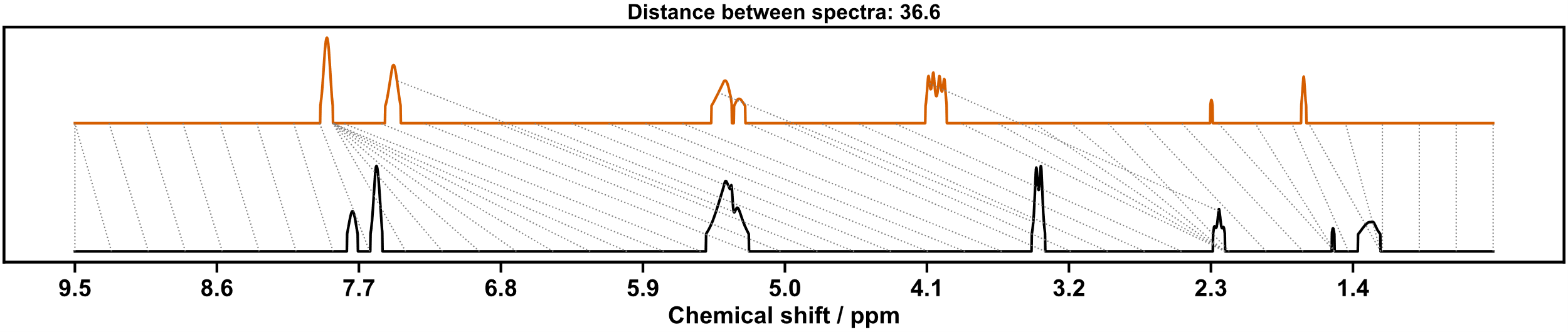


**Figure S 2:** Dynamic time warp comparison of screening **6** (top) with combined ^1^H NMR spectra of amine **1** and isocyanate **5**.


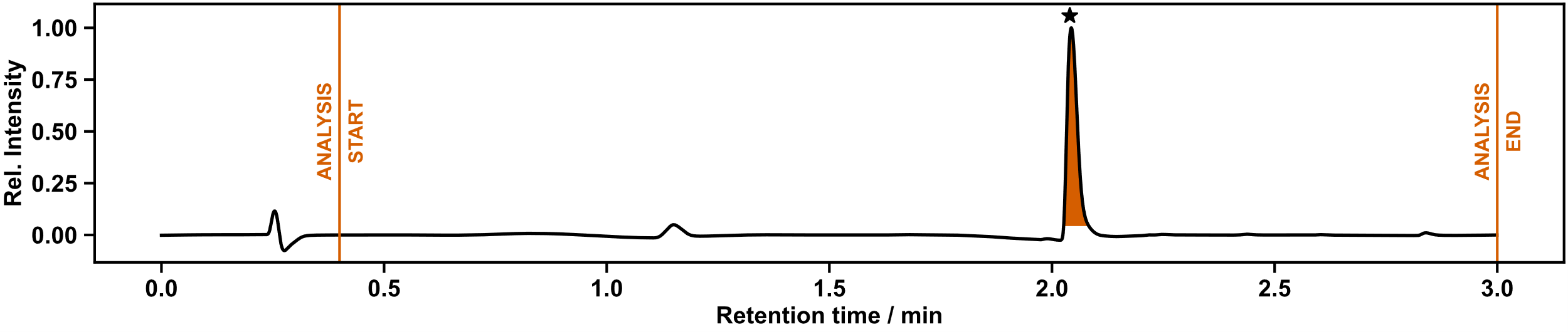


**Figure S 3:** UPLC chromatogram of screening **6.**


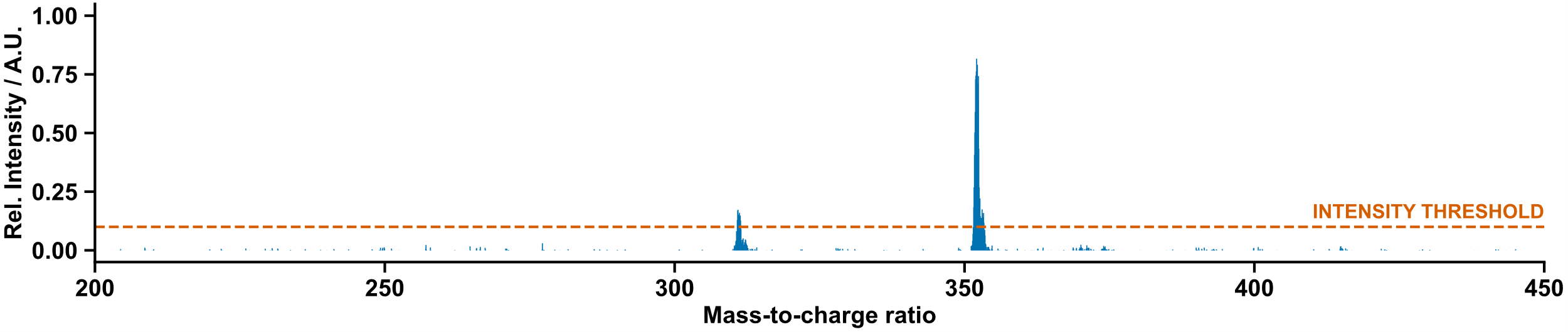


**Figure S 4:** Mass spectrum of screening **6**.

#### Manual Characterization (for comparison with automated analysis)

**^1^H NMR** (400 MHz, CD_3_OD) δ 8.02 – 7.97 (m, 2H, H^5^), 7.49 – 7.44 (m, 1H, H^4^), 4.00 (d, *J* = 2.5 Hz, 2H, H^8^), 2.58 (t, *J* = 2.5 Hz, 1H, H^10^).

**^13^C NMR** (101 MHz, CD_3_OD) δ 156.87 (C^7^), 143.14 (C^6^), 133.13 (q, *J* = 33.1 Hz, C^3^), 124.78 (q, *J* = 271.8 Hz, C^2^), 119.31 – 118.83 (m, C^5^), 115.89 – 115.46 (m, C^4^), 81.21 (C^9^), 72.11 (C^10^), 30.16 (C^8^).

**^19^F NMR** (376 MHz, CD_3_OD) δ -64.65 (F^1^).


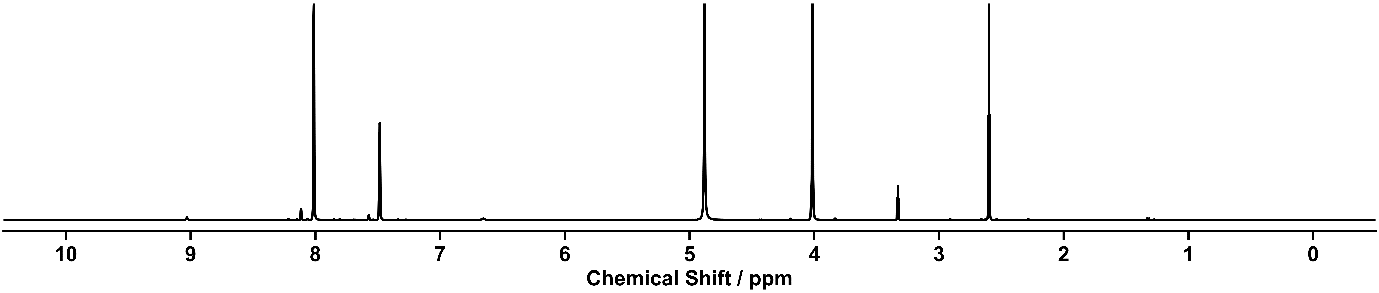
**HRMS** calculated for C_12_H_9_F_6_N_2_O+: 311.0614; found: 311.0631.

**Figure S 5:** ^1^H NMR spectrum (400 MHz, CD_3_OD) of urea **6.**


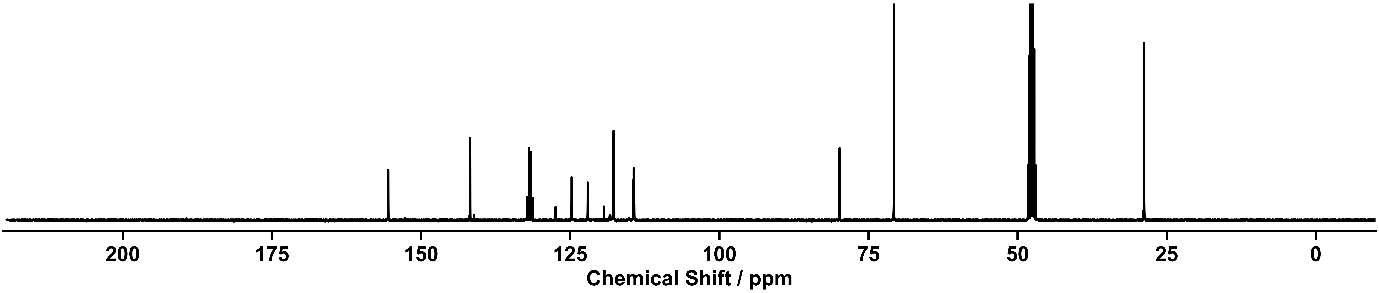
**Figure S 6:** ^13^C NMR spectrum (101 MHz, CD_3_OD) of urea **6.**

### Screening (7)

#### Outcome of Automation


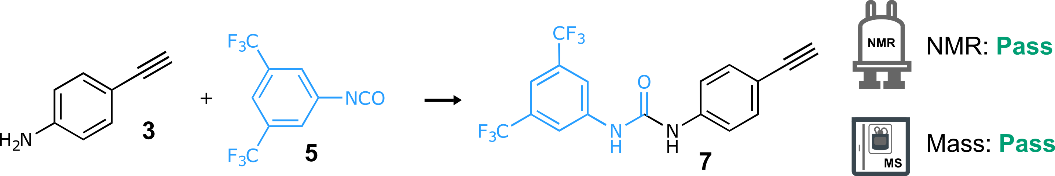


**Scheme S 3:** Synthesis of urea **7** from amine **3** and isocyanate **5**. Reaction conditions: CH_2_Cl_2_, r.t., 12 h.

| *NMR*: Pass | *MS*: Pass |
| --- | --- |
| *DTW distance*: 110.2711 | *retention time*: 2.33 min  *LC area*: 100% |
|  | *ion observed*: [**7**+H]^+^  *m/z expected*: 373.08  *m/z measured*: 373.24  *ion observed*: [**7**+CH_3_CN+H]^+^  *m/z expected*: 414.10  *m/z measured*: 414.22 |

**Table S 2:** Summary of automated decision-maker outcomes for ^1^H NMR spectroscopy and ULPC-MS spectrometry.


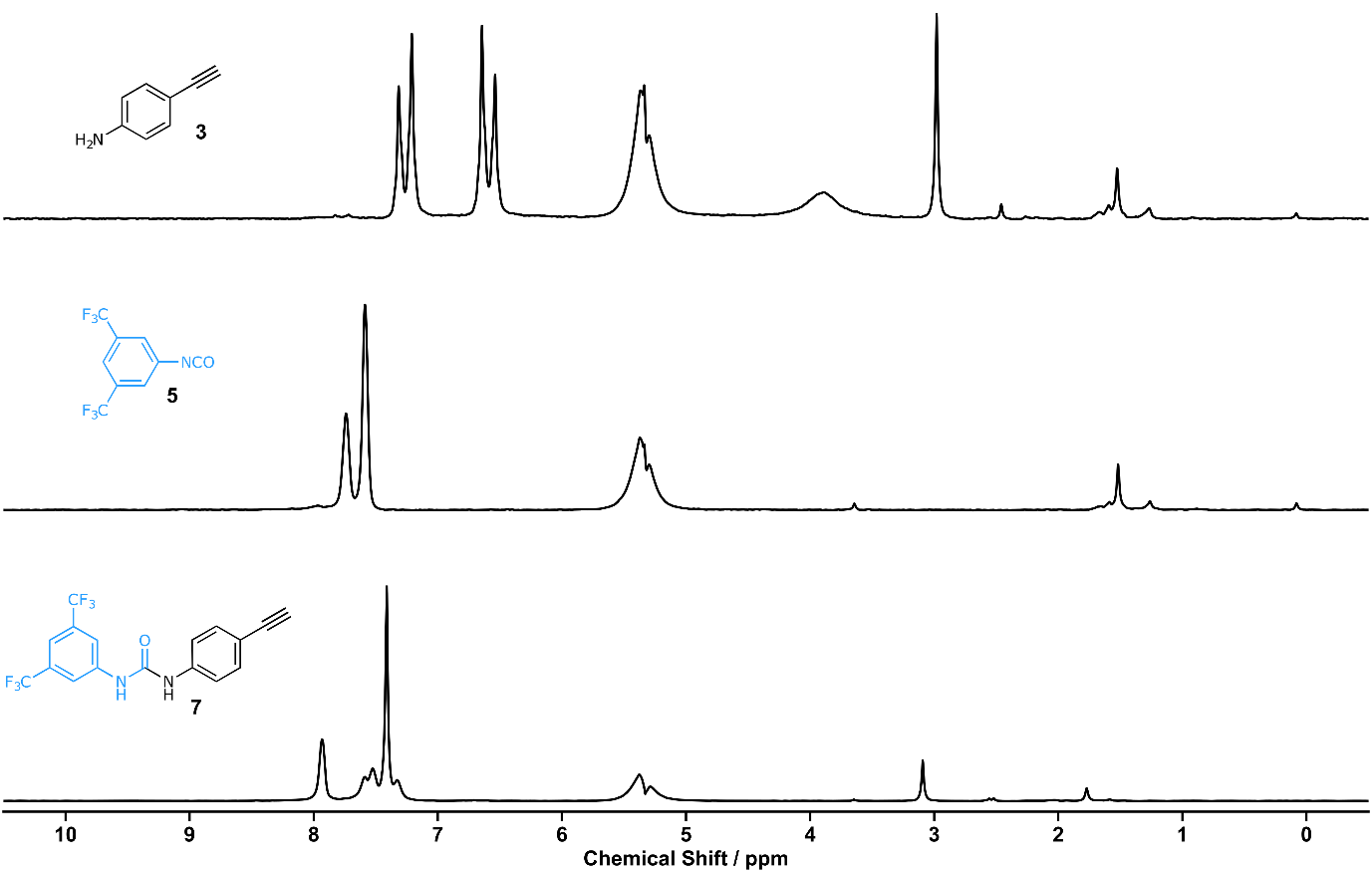


**Figure S 7:** ^1^H NMR (80 MHz, CH_2_Cl_2_) of aniline **3** (top), isocyanate **5** (middle), screening **7** (bottom).


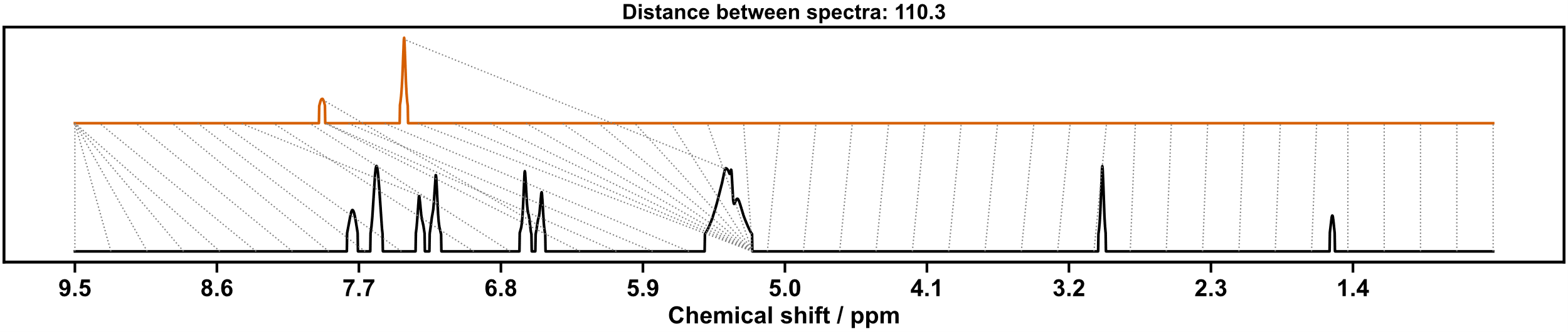


**Figure S 8:** Dynamic time warp comparison of screening **7** (top) with combined ^1^H NMR spectra of aniline **3** and isocyanate **5.**


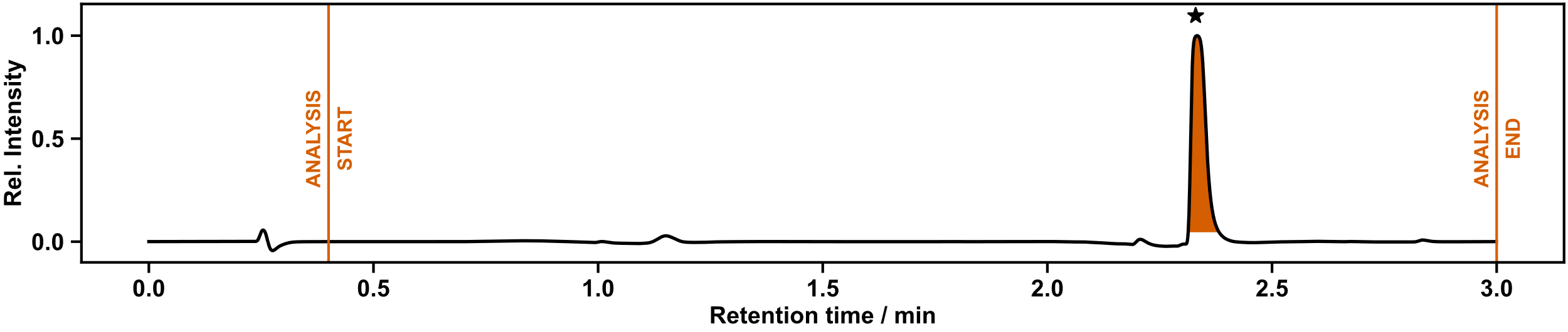


**Figure S 9:**UPLC chromatogram of screening **7**.


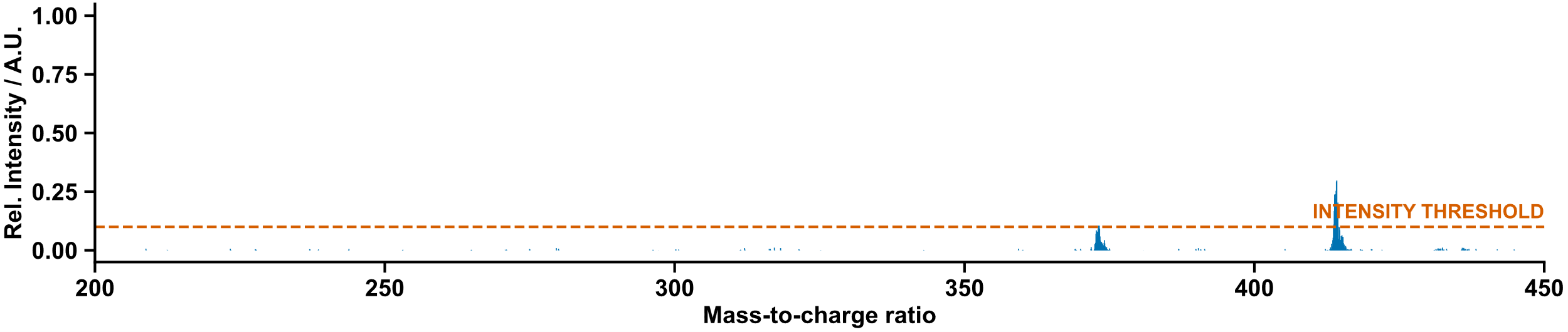


**Figure S 10:** Mass spectrum of screening **7**.

#### Manual Characterization (for comparison with automated analysis)

**^1^H NMR** (400 MHz, CD_3_OD) δ 8.03 (s, 2H), 7.54 – 7.47 (m, 1H), 7.45 – 7.39 (m, 2H), 7.40 – 7.32 (m, 2H), 3.34 (s, 1H).

**^13^C NMR** (101 MHz, CD_3_OD) δ 154.14, 142.71, 140.54, 133.69, 133.14 (q, *J* = 33.0 Hz), 124.75 (q, *J* = 271.9 Hz), 119.91, 119.66 – 119.18 (m), 118.03, 116.27 – 115.91 (m), 84.29, 77.65.

**HRMS** calculated for C_17_H_11_F_6_N_2_O+: 373.0770; found: 373.0748.


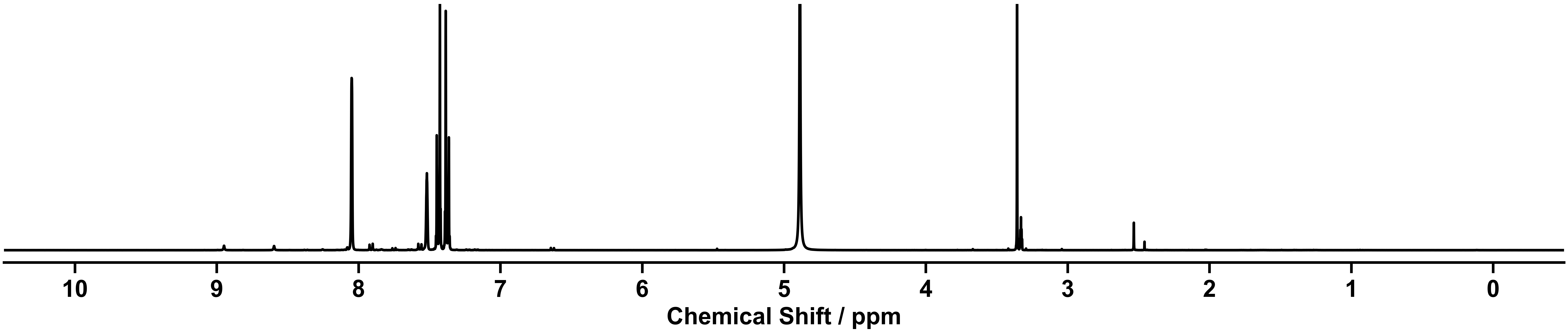
^1^H NMR and ^13^C NMR spectroscopic data were consistent with literature values.^1^

**Figure S 11:** ^1^H spectrum NMR (400 MHz, CD_3_OD) of urea **7.**

**
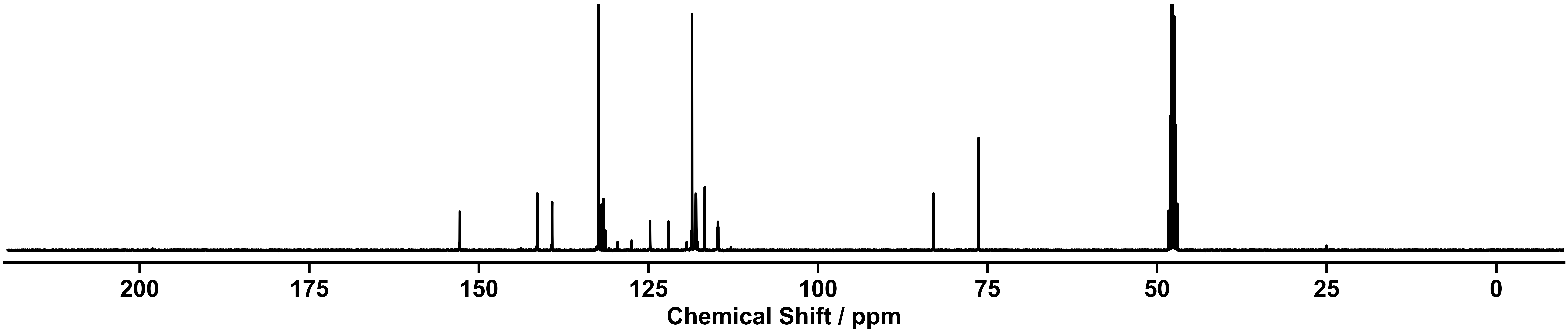
**

**Figure S 12**: ^13^C spectrum NMR (101 MHz, CD_3_OD) of urea **7.**

### Screening (8)

#### Outcome of Automation


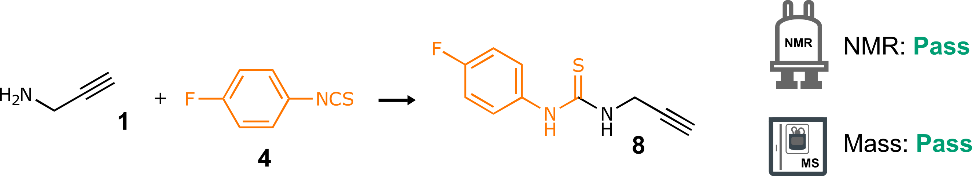


**Scheme S 4:** Synthesis of thiourea **6** from amine **1** and isothiocyanate **4**. Reaction conditions: CH_2_Cl_2_, r.t., 12 h.

| *NMR*: Pass | *MS*: Pass |
| --- | --- |
| *DTW distance*: 46.4864 | *retention time*: 1.14 min  *LC area*: 64% |
|  | *ion observed*: [**8**+H]^+^  *m/z expected*: 209.05  *m/z measured*: 208.99 |

**Table S 3:** Summary of automated decision-maker outcomes for ^1^H NMR spectroscopy and ULPC-MS spectrometry.


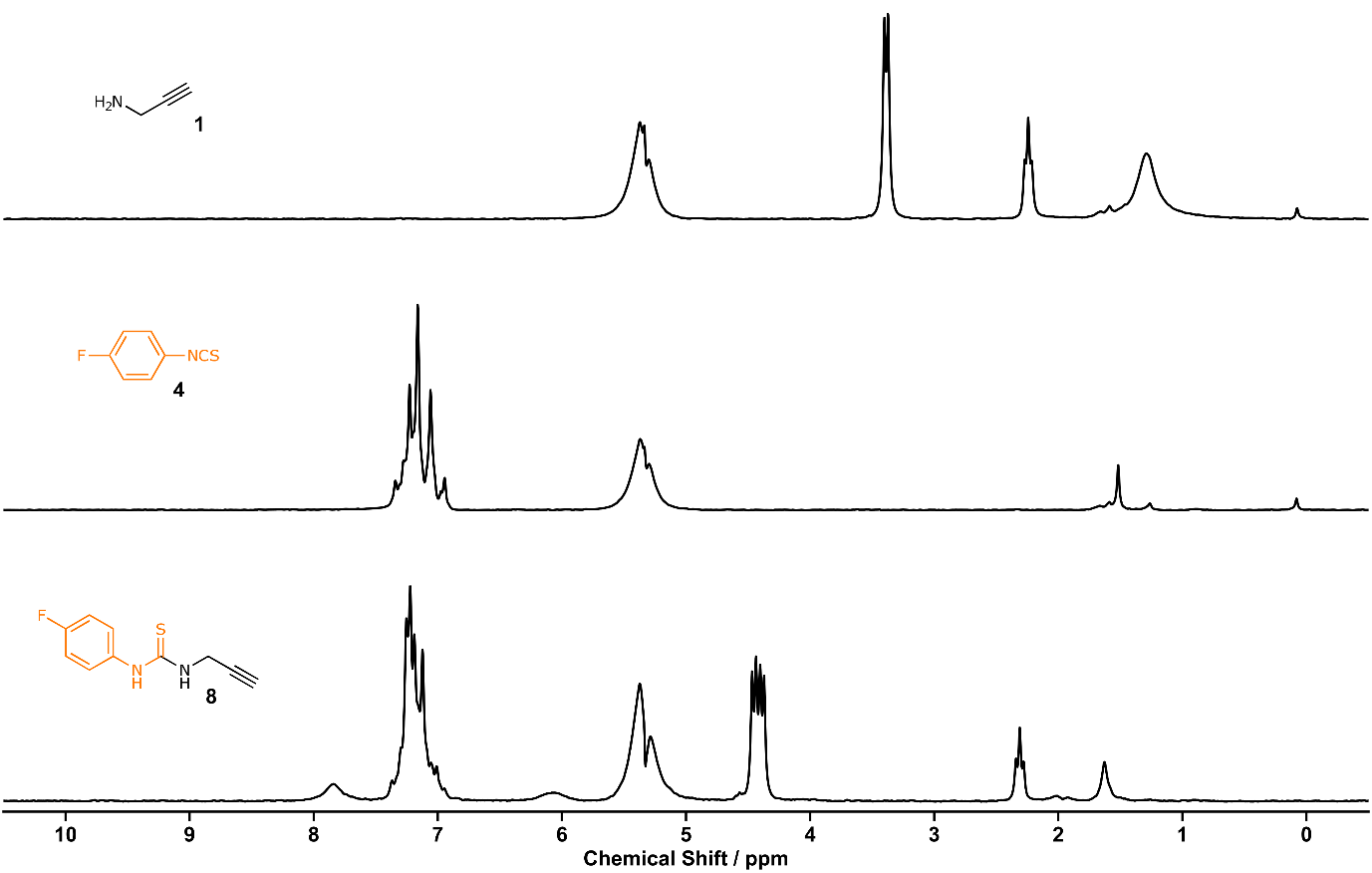


**Figure S 13:** ^1^H NMR (80 MHz, CH_2_Cl_2_) of amine **1** (top), isothiocyanate **4**, screening **8** (bottom).


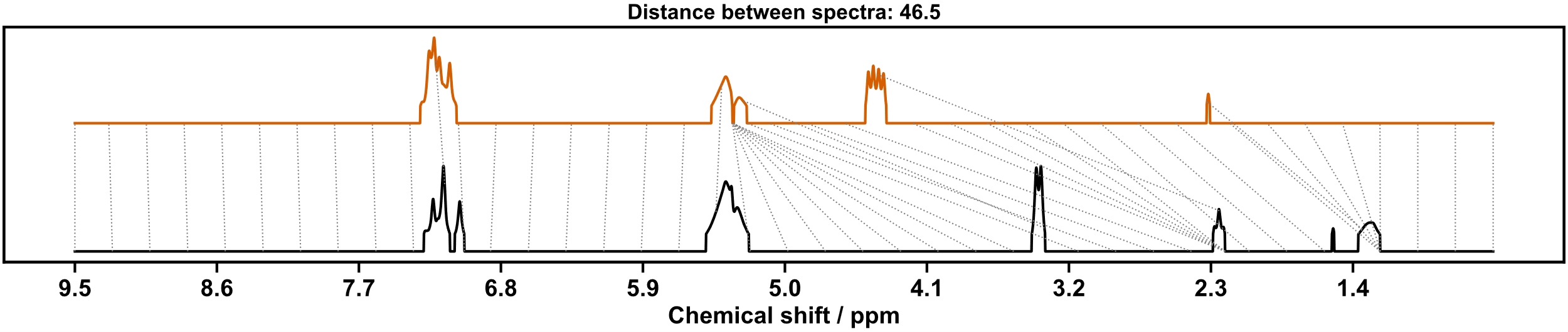


**Figure S 14:** Dynamic time warp comparison of screening **8** (top) with combined ^1^H NMR spectra of amine **1** and isothiocyanate **4**.


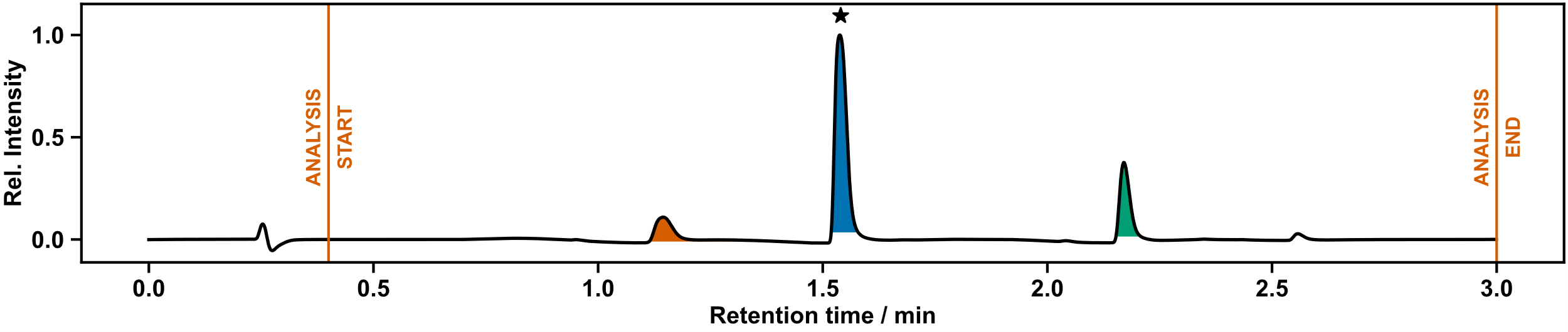


**Figure S 15:**UPLC chromatogram of screening **8**.


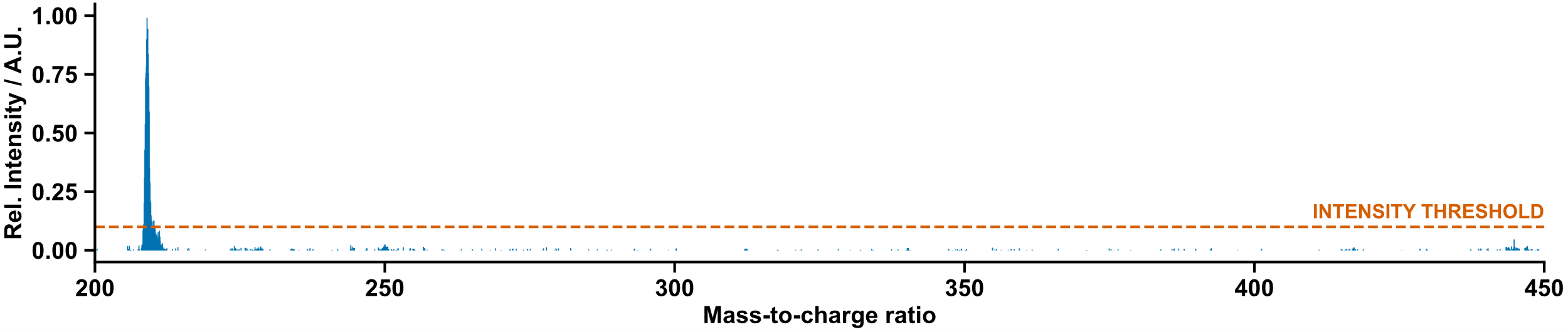


**Figure S 16:** Mass spectrum of screening **8**.

#### Manual Characterization (for comparison with automated analysis)

**^1^H NMR** (400 MHz, CD_2_Cl_2_) δ 8.22 (s, 1H, H^6^), 7.31 – 7.20 (m, 2H, H^4^), 7.20 – 7.02 (m, 2H, H^3^), 6.08 (s, 1H, H^8^), 4.43 (dd, *J* = 5.2, 2.6 Hz, 2H, H^9^), 2.31 (t, *J* = 2.6 Hz, 1H, H^11^).

**^13^C NMR** (101 MHz, CD_2_Cl_2_) δ 181.49 (C^7^), 161.90 (d, *J* = 247.3 Hz, C^2^), 132.44 (d, *J* = 3.2 Hz, C^5^), 128.24 (d, *J* = 8.7 Hz, C^4^), 117.30 (d, *J* = 22.9 Hz, C^3^), 79.22 (C^10^), 72.35 (C^11^), 35.20 (C^9^).

**^19^F NMR** (376 MHz, CD_2_Cl_2_) δ -114.14 (F^1^).


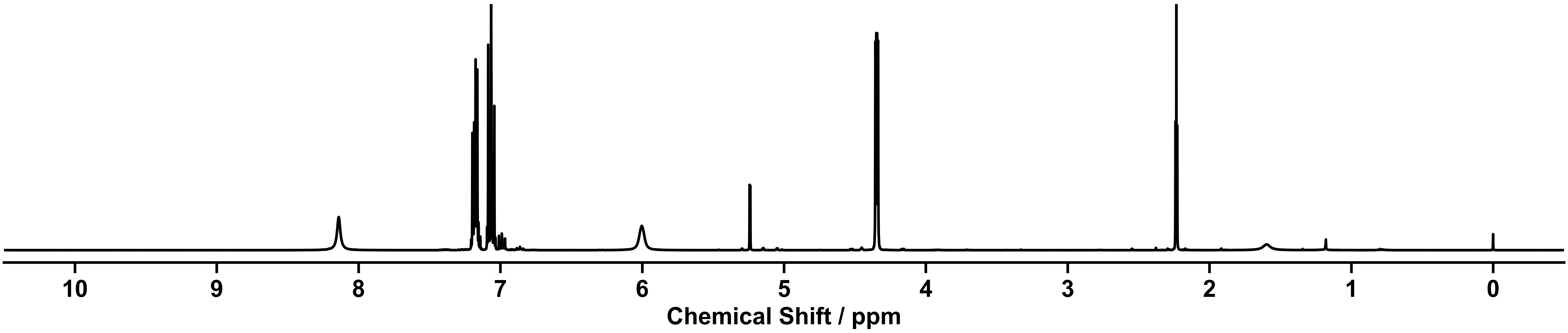
**HRMS** calculated for C_10_H_10_FN_2_S+: 209.0543; found: 209.0540.


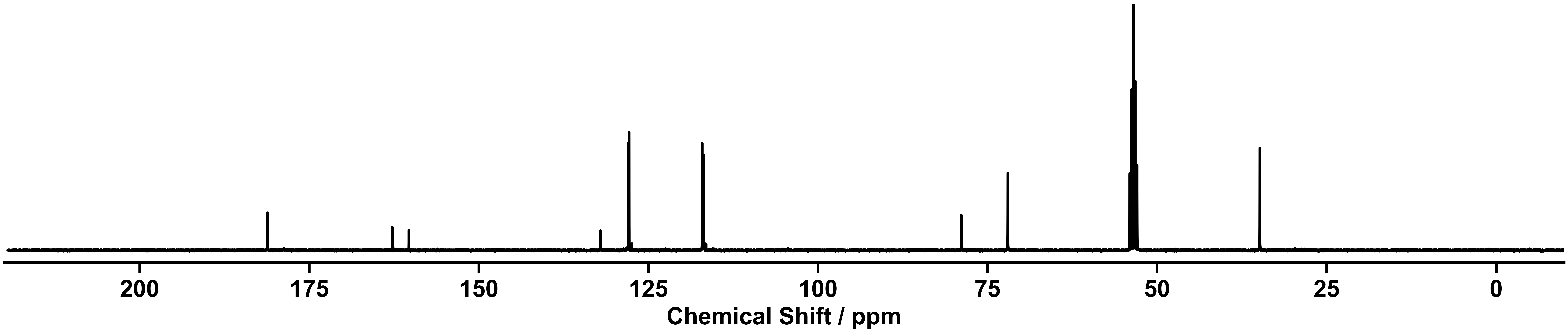
**Figure S 17:** ^1^H NMR spectrum (400 MHz, CD_2_Cl_2_) of thiourea **8.**

**Figure S 18:** ^13^C NMR spectrum (101 MHz, CD_2_Cl_2_) of thiourea **8.**

### Screening (9)

#### Outcome of Automation


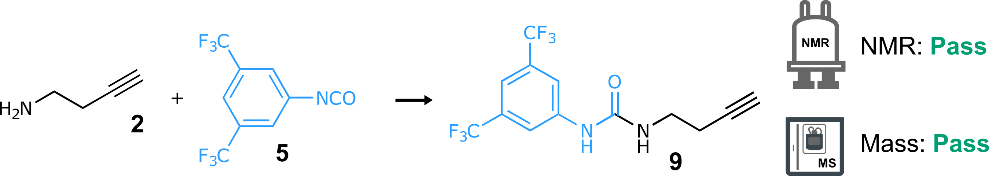


**Scheme S 5:** Synthesis of urea **9** from amine **2** and isocyanate **5**. Reaction conditions: CH_2_Cl_2_, r.t., 12 h.

| *NMR*: Pass | *MS*: Pass |
| --- | --- |
| *DTW distance*: 52.1599 | *retention time*: 2.09 min  *LC area*: 100% |
|  | *ion observed*: [**9**+H]^+^  *m/z expected*: 325.08  *m/z measured*: 325.05  *ion observed*: [**9**+CH_3_CN+H]^+^  *m/z expected*: 366.10  *m/z measured*: 366.09 |

**Table S 4:** Summary of automated decision-maker outcomes for ^1^H NMR spectroscopy and ULPC-MS spectrometry.


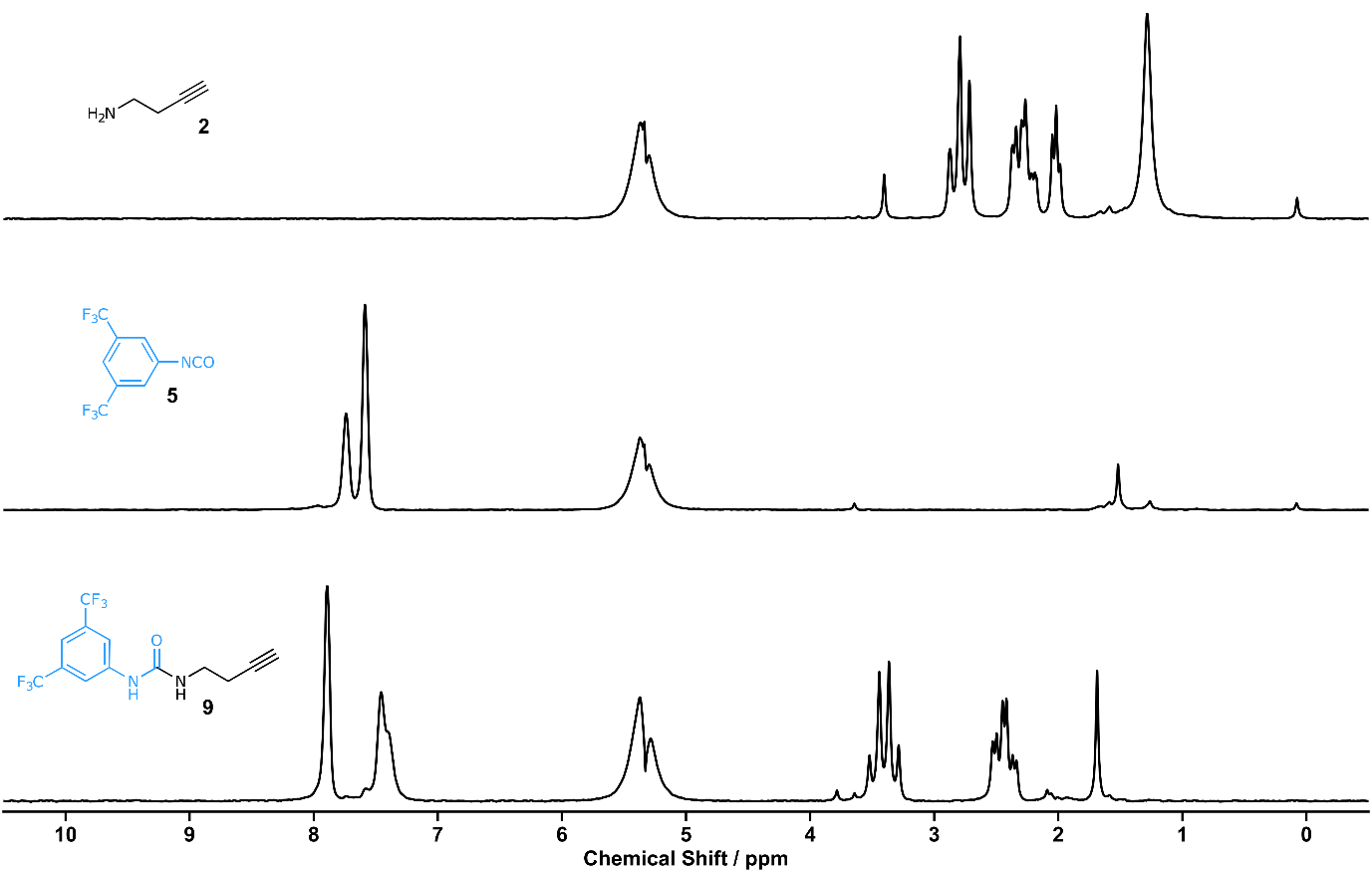


**Figure S 19:** ^1^H NMR spectrum (80 MHz, CH_2_Cl_2_) of amine **2** (top), isocyanate **5** (middle), screening **9** (bottom).


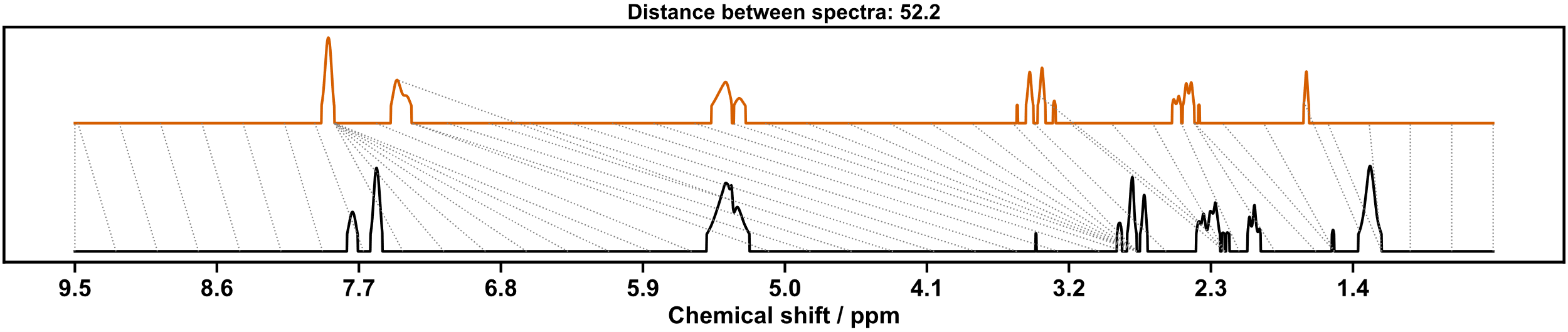


**Figure S 20:** Dynamic time warp comparison of screening **9** (top) with combined ^1^H NMR spectra of amine **2** and isocyanate **5**.

**Figure S 21:**UPLC chromatogram of screening **9**
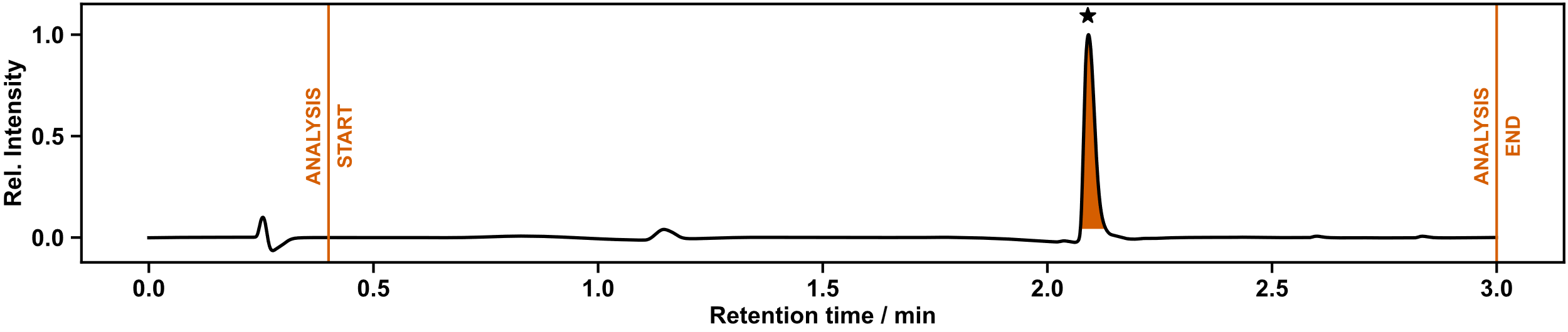
.

**
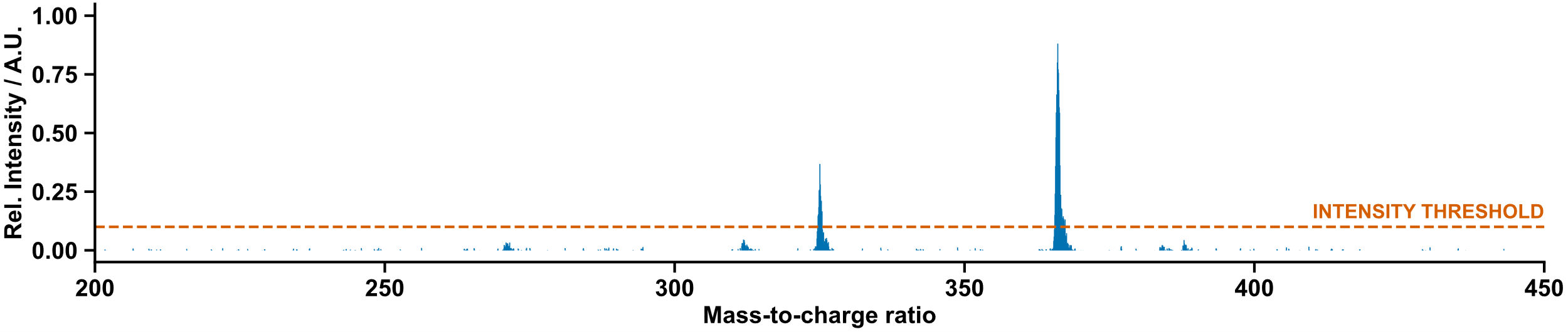
Figure S 22:** Mass spectrum of screening **9**.

#### Manual Characterization (for comparison with automated analysis)

**^1^H NMR** (400 MHz, CD_3_OD) δ 8.01 – 7.96 (m, 2H, H^5^), 7.48 – 7.43 (m, 1H, H^4^), 3.36 (t, *J* = 6.8 Hz, 2H, H^8^), 2.42 (td, *J* = 6.8, 2.7 Hz, 2H, H^9^), 2.31 (t, *J* = 2.7 Hz, 1H, H^11^).

**^13^C NMR** (101 MHz, CD_3_OD) δ 157.25 (C^7^), 143.31(C^6^), 133.13 (q, *J* = 33.1 Hz, C^3^), 124.81 (q, *J* = 271.8 Hz, C^2^), 119.25 – 118.69 (m, C^5^), 115.69 – 115.35 (m, C^4^), 82.30 (C^10^), 70.96 (C^11^), 39.91 (C^8^), 20.52 (C^9^).

**^19^F NMR** (376 MHz, CD_3_OD) δ -64.65 (F^1^).


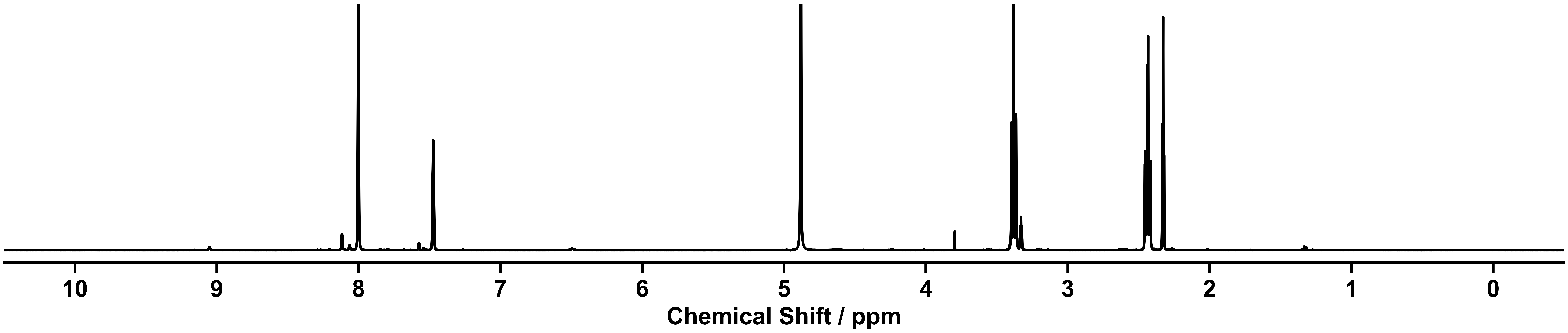
**HRMS** calculated for C_13_H_11_F_6_N_2_O+: 325.0770; found: 325.0744.

**Figure S 23:** ^1^H NMR spectrum (400 MHz, CD_3_OD) of urea **9**.

**
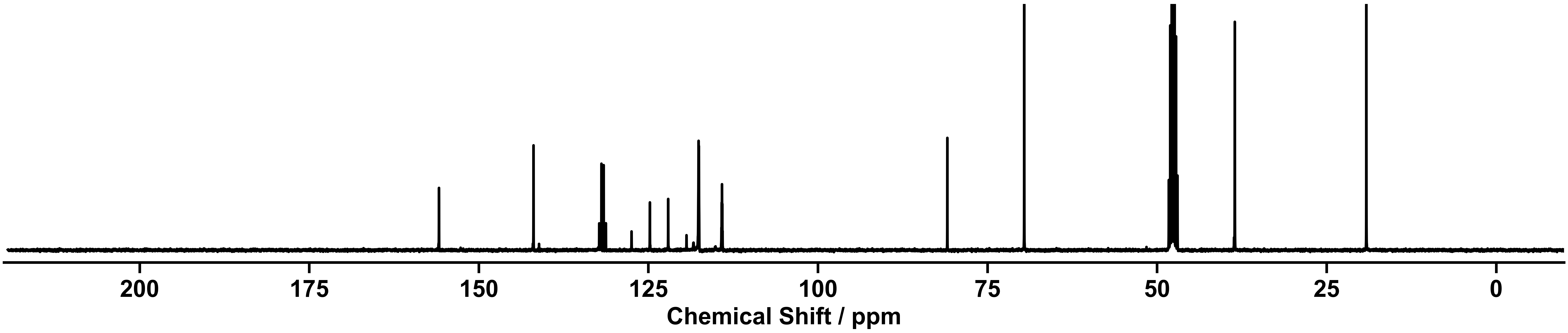
Figure S 24**: ^13^C NMR spectrum (101 MHz, CD_3_OD) of urea **9**.

### Screening (10)

#### Outcome of Automation


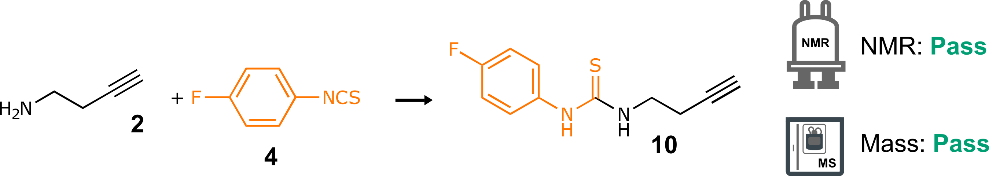


**Scheme S 6:** Synthesis of thiourea **10** from amine **2** and isothiocyanate **4**. Reaction conditions: CH_2_Cl_2_, r.t., 12 h.

| *NMR*: Pass | *MS*: Pass |
| --- | --- |
| *DTW distance*: 35.8181 | *retention time*: 1.64 min  *LC area*: 73% |
|  | *ion observed*: [**10**+H]^+^  *m/z expected*: 223.07  *m/z measured*: 223.04 |

**Table S 5:** Summary of automated decision-maker outcomes for ^1^H NMR spectroscopy and ULPC-MS spectrometry.


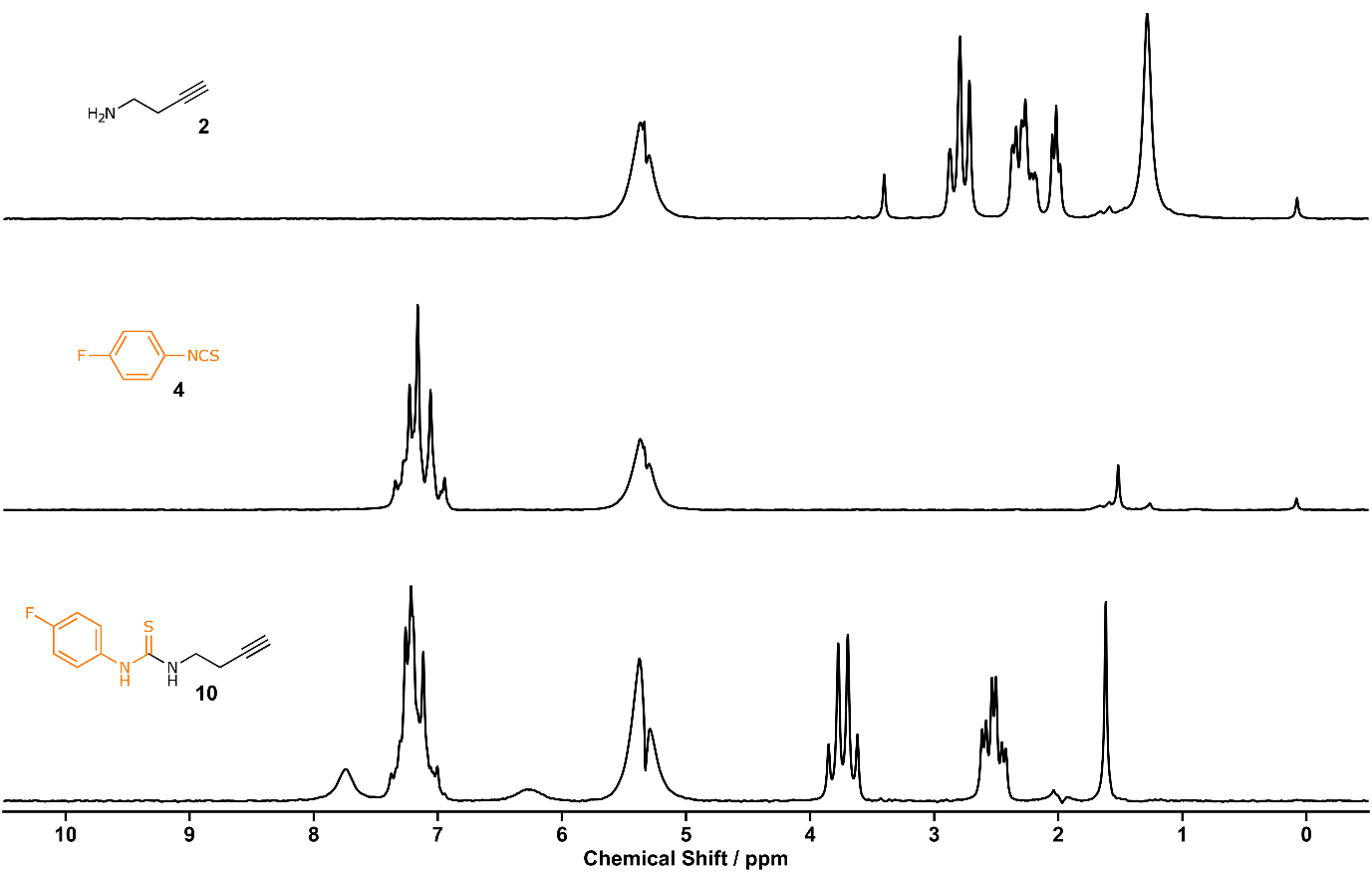


**Figure S 25:** ^1^H NMR spectrum (80 MHz, CH_2_Cl_2_) of amine **2** (top), isothiocyanate **4** (middle), screening **10** (bottom).


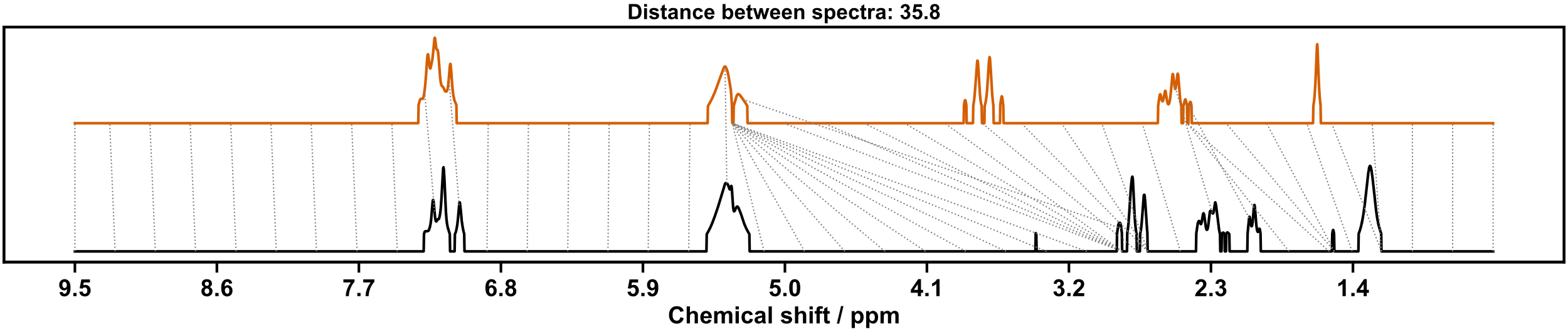


**Figure S 26:** Dynamic time warp comparison of screening **10** (top) with combined ^1^H NMR spectra of amine **2** and isothiocyanate **4.**


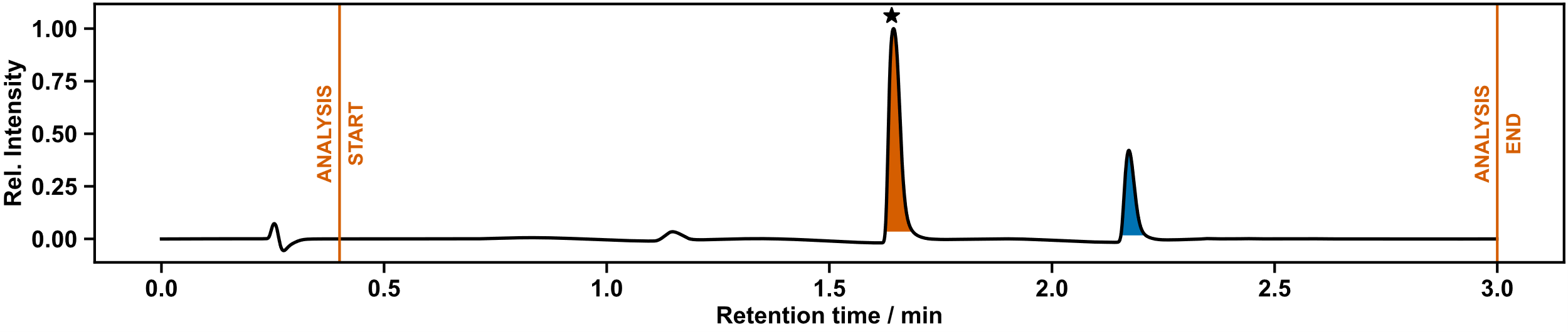


**Figure S 27:** UPLC chromatogram of screening **10**.


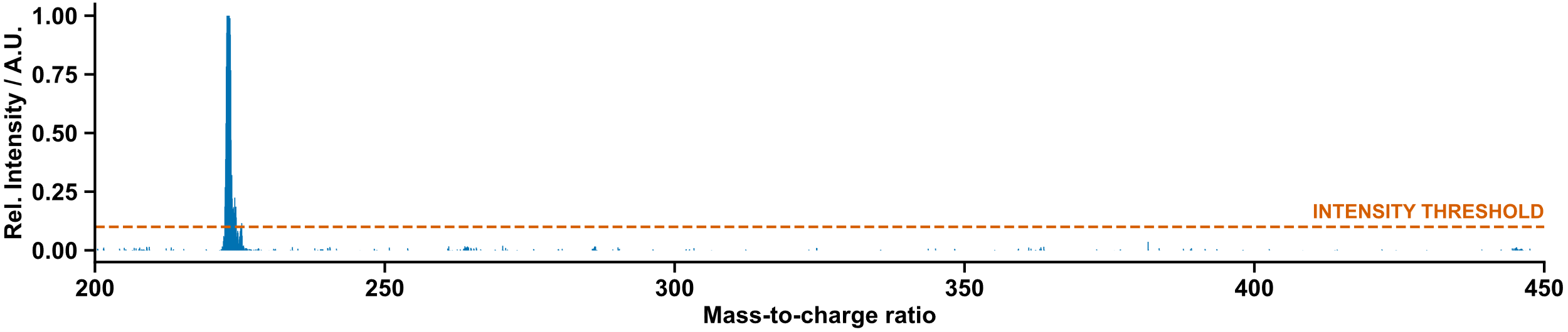


**Figure S 28:** Mass spectrum of screening **10**.

#### Manual Characterization (for comparison with automated analysis)

**^1^H NMR** (400 MHz, CD_3_OD) δ 7.38 – 7.28 (m, 2H, H^4^), 7.15 – 7.04 (m, 2H, H^3^), 3.69 (t, *J* = 6.9 Hz, 2H, H^7^), 2.52 (td, *J* = 6.9, 2.7 Hz, 2H, H^8^), 2.31 (t, *J* = 2.7 Hz, 1H, H^10^).

**^13^C NMR** (101 MHz, CD_3_OD) δ 182.71 (C^6^), 162.03 (d, *J* = 244.0 Hz, C^2^), 135.51 (C^5^), 128.21 (d, *J* = 8.4 Hz, C^4^), 116.81 (d, *J* = 22.9 Hz, C^3^), 82.40 (C^9^), 70.96 (C^10^), 44.40 (C^7^), 19.42 (C^8^).

**^19^F NMR** (376 MHz, CD_3_OD) δ -118.23 (F^1^).

**HRMS** calculated for C_11_H_12_FN_2_S+: 223.0700; found: 223.0691.

**
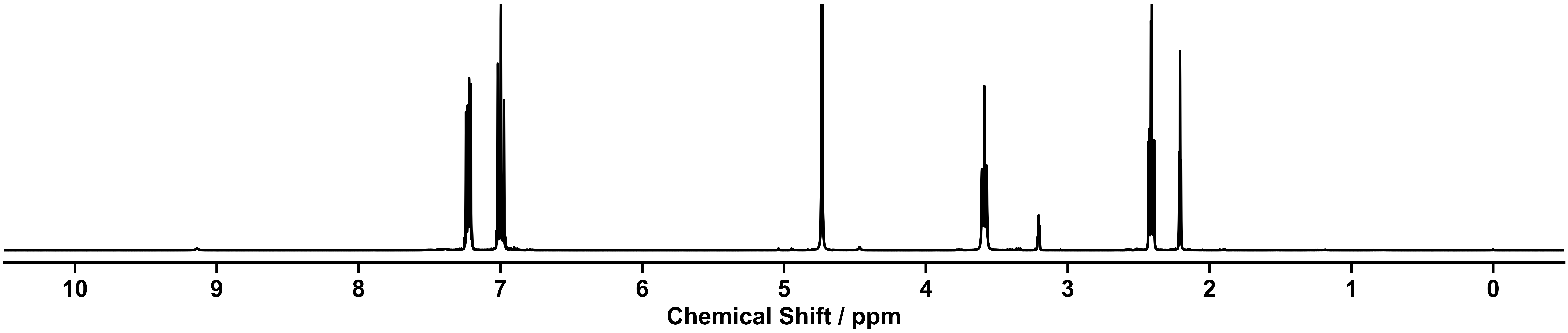
**

**Figure S 29**: ^1^H NMR spectrum (400 MHz, CD_3_OD) of thiourea **10**.


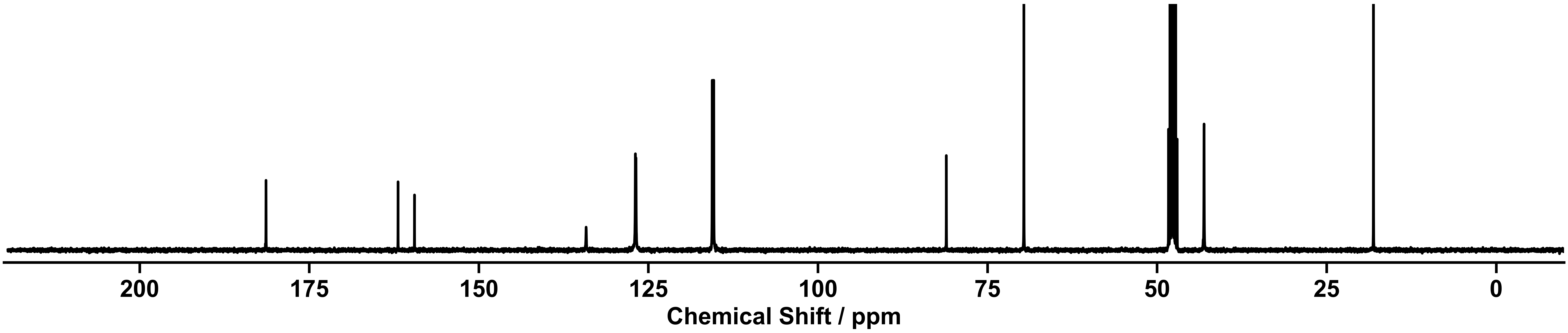


**Figure S 30**: ^13^C NMR spectrum (101 MHz, CD_3_OD) of thiourea **10**.

### Screening (11)


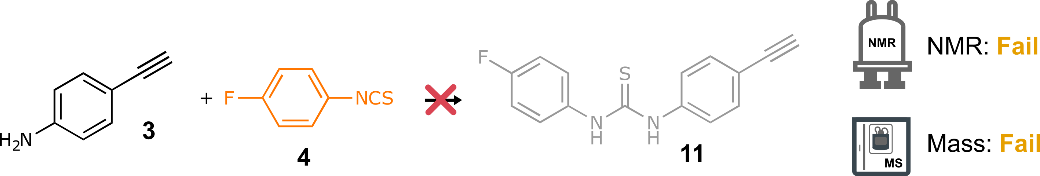


**Scheme S 7:** Attempted synthesis of thiourea **11** from amine **3** and isothiocyanate **4**. Reaction conditions: CH_2_Cl_2_, r.t., 12 h.

| *NMR*: Fail | *MS*: Fail |
| --- | --- |
| *DTW distance*: 23.62613 | *retention time*: 1.12 min  *LC area*: 28%  *retention time*: 2.17 min  *LC area*: 65% |
|  | *No matching ions observed* |

**Table S 6:** Summary of automated decision-maker outcomes for ^1^H NMR spectroscopy and ULPC-MS spectrometry.


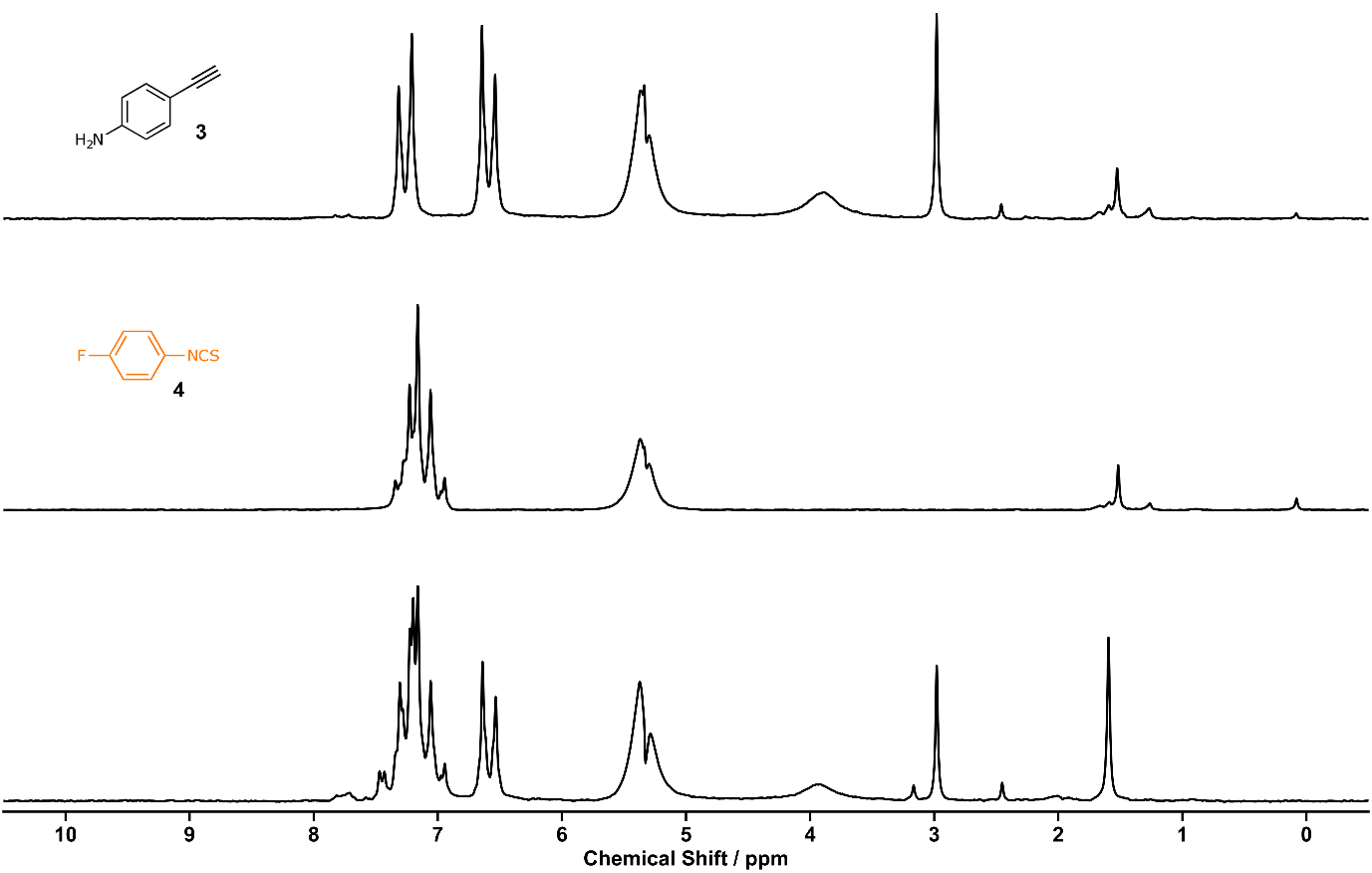


**Figure S 31:** ^1^H NMR spectrum (80 MHz, CH_2_Cl_2_) of amine **3** (top), isothiocyanate **4** (middle), screening **11** (bottom).


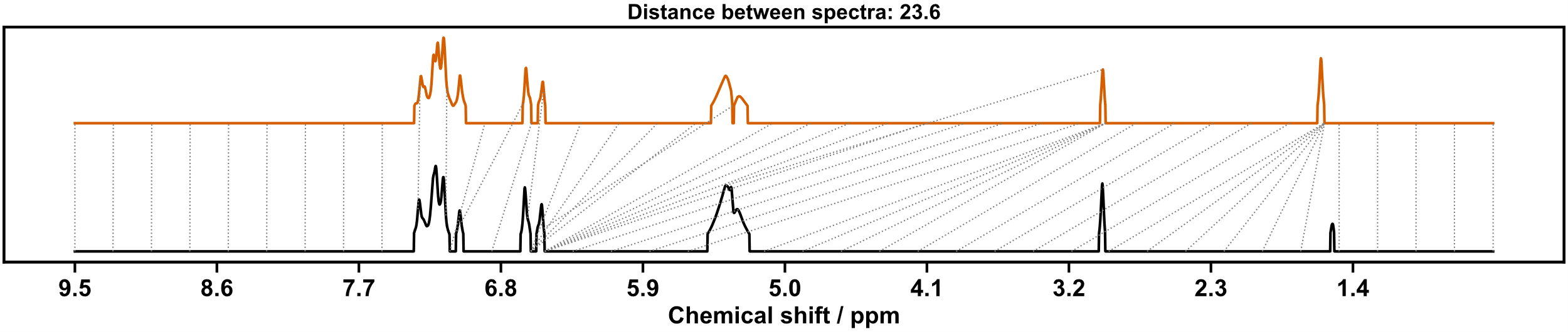


**Figure S 32:** Dynamic time warp comparison of screening **11** (top) with combined ^1^H NMR spectra of aniline **3** and isothiocyanate **4**.


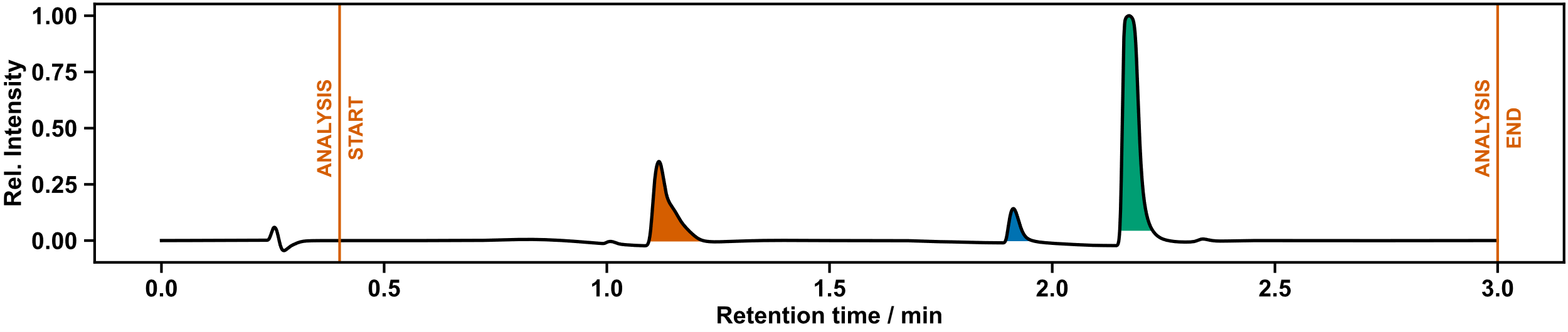
 **Figure S 33**:UPLC chromatogram of screening **10.** Peaks matching required conditions did not correspond with matching m/z values.

## (Thio)rea Scale-up Synthesis

The scale-up was performed in an analogous way to the screening reaction, except larger volumes of the stock solutions were transferred. Following preparation of the stock solutions (200 mM) as in the screening stage, the aliquots (6 mL) were dispensed into 100 mL vials to scale up (thio)ureas **6 – 10**, and shaken overnight (12 h, r.t.). CH_2_Cl_2_ (30 mL) was dispensed to the reactions vials, aliquots were taken for automated analysis identically to the screening stage. Aliquots (10 mL each) of the scaled reactions were dispensed to pre-loaded 20 mL ISynth vials for the next stage of the synthesis for Sonogashira and CuAAc reactions.

The vials for Sonogashira coupling reactions were concentrated using the Chemspeed’s vacuum capabilities to remove CH_2_Cl_2_. The vials used for the CuAAc diversification were pre-loaded with azidothymidine, due to poor solubility in IPA/water which prevented facile automated dispensing.

Spectroscopic data for the scale-up reactions were consistent with the screening experiments. The reaction conditions were adapted from literature procedures.^2^

***N.B.*:** On occasion, we found that due to poor sealing of the reactor block, some residual solvent remained following the evaporation stage, and these samples were manually concentrated in the next stage when reagents for the parallel diversification reactions were being loaded.

## (Thio)urea Scale-up Results

### Scale-up (6)


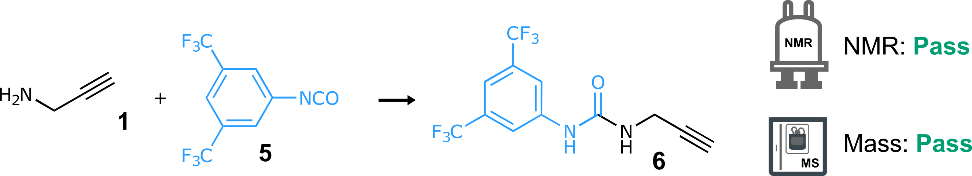


**Scheme S 8**: Synthesis of urea **6** from amine **1** and isocyanate **5**. Reaction conditions: CH_2_Cl_2_, r.t., 12 h.

| *NMR*: Pass | *MS*: Pass |
| --- | --- |
| *Peaks matching reference* | *retention time*: 2.04 min  *LC area*: 100% |
|  | *ion observed*: [**6**+H]^+^  *m/z expected*: 311.06  *m/z measured*: 311.12  *ion observed*: [**6**+CH_3_CN+H]^+^  *m/z expected*: 352.09  *m/z measured*: 352.15 |

**Table S 7:** Summary of automated decision-maker outcomes for ^1^H NMR spectroscopy and ULPC-MS spectrometry.


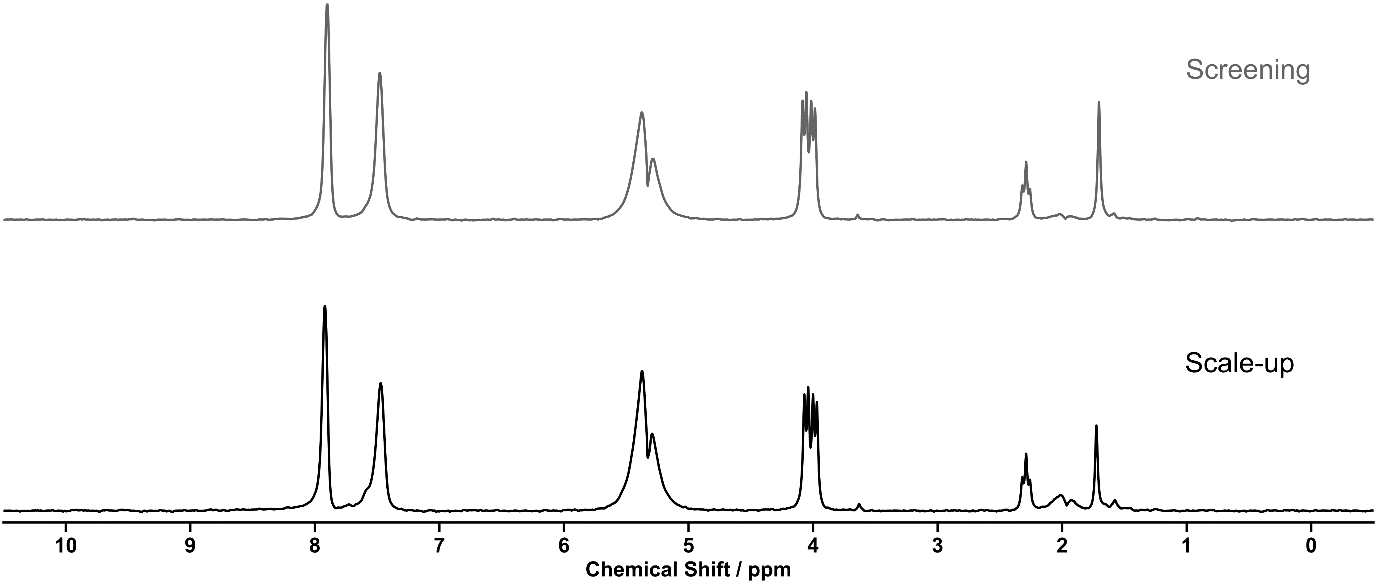


**Figure S 34:** ^1^H NMR spectrum (80 MHz, CH_2_Cl_2_) of screening **6** (top), scale-up **6** (bottom).


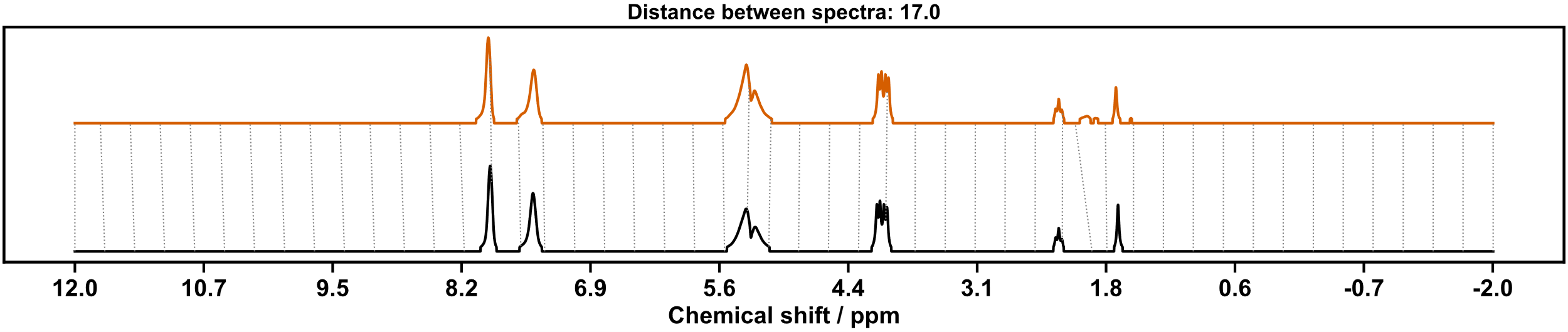


**Figure S 35:** Dynamic time warp comparison of screening **6** (top) with scale-up **6.**


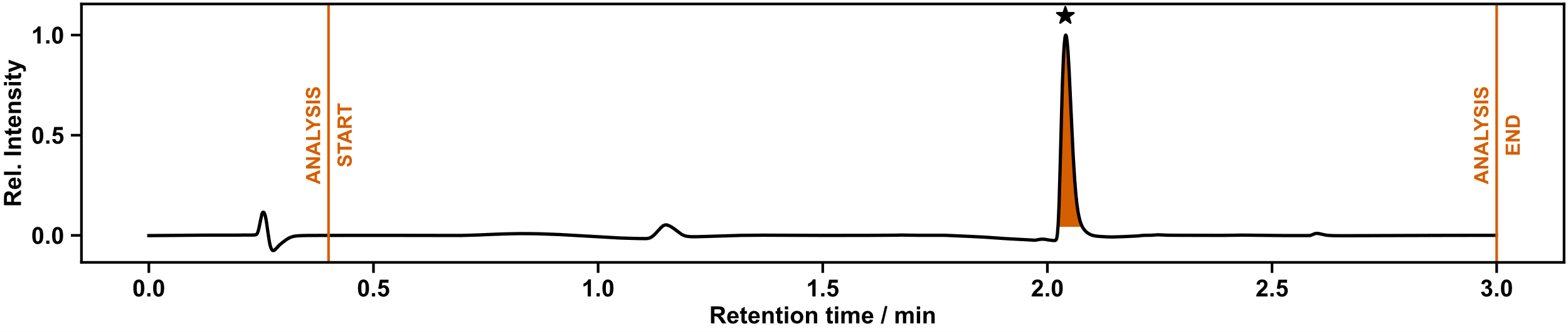


**Figure S 36**: UPLC chromatogram of scale-up **6.**


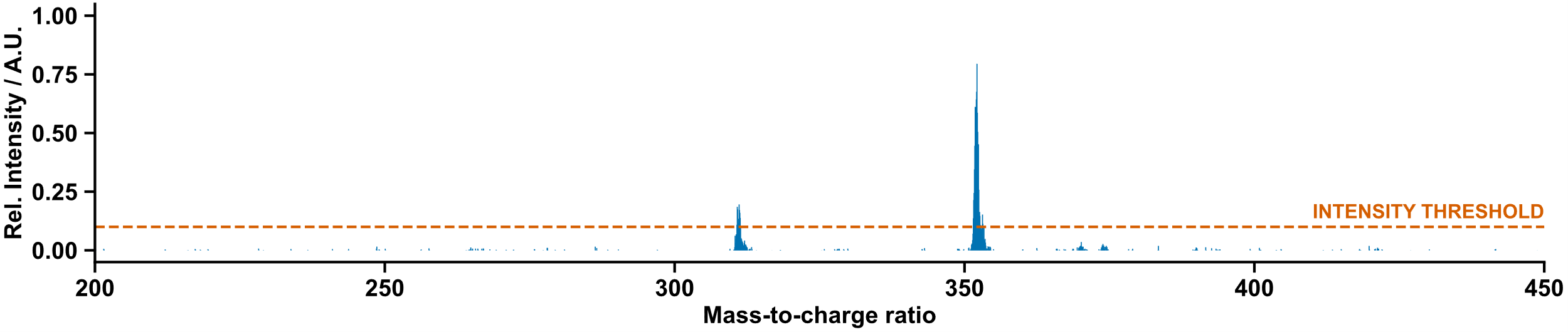


**Figure S 37:** Mass spectrum of screening sample **10**.

### Scale-up (7)


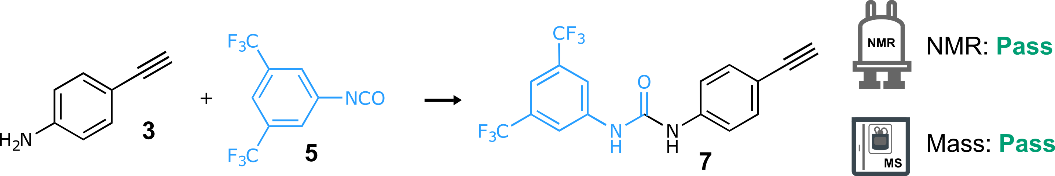


**Scheme S 9:** Synthesis of urea **7** from amine **3** and isocyanate **5**. Reaction conditions: CH_2_Cl_2_, r.t., 12 h.

| *NMR*: Pass | *MS*: Pass |
| --- | --- |
| *Peaks matching reference* | *retention time*: 2.33 min  *LC area*: 100% |
|  | *ion observed*: [**7**+H]^+^  *m/z expected*: 373.08  *m/z measured*: 372.99  *ion observed*: [**7**+CH_3_CN+H]^+^  *m/z expected*: 414.10  *m/z measured*: 414.09 |


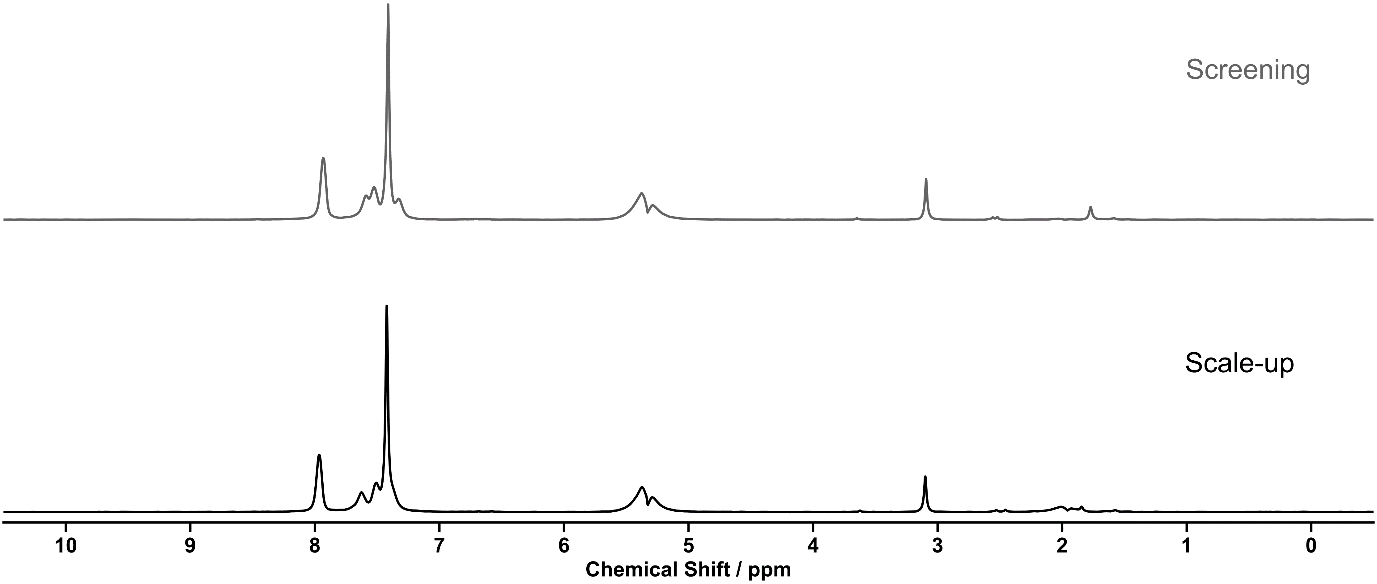
 **Table S 8:** Summary of automated decision-maker outcomes for ^1^H NMR spectroscopy and ULPC-MS spectrometry.


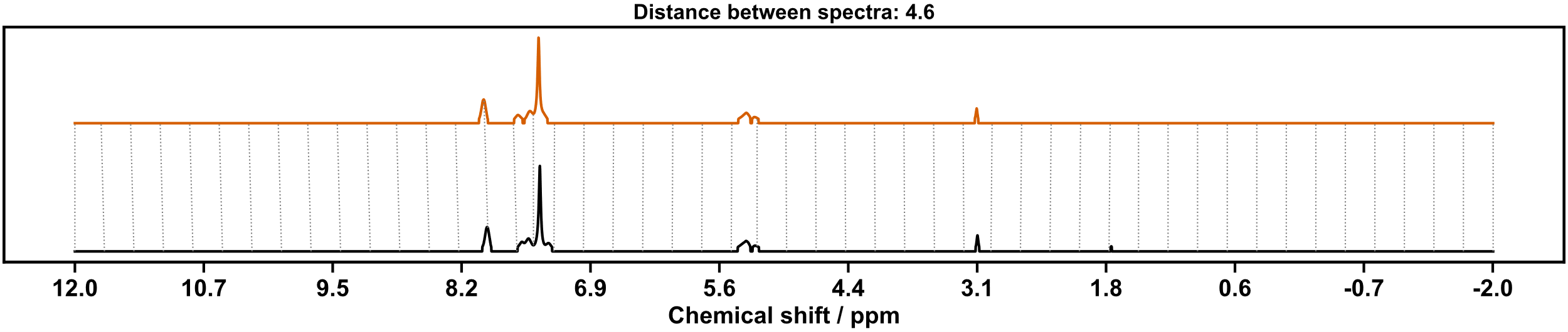
**Figure S 38:** ^1^H NMR spectrum (80 MHz, CH_2_Cl_2_) of screening **7** (top), scale-up **7** (bottom).

**Figure S 39:** Dynamic time warp comparison of screening **7** (top) with scale-up **7.**

**
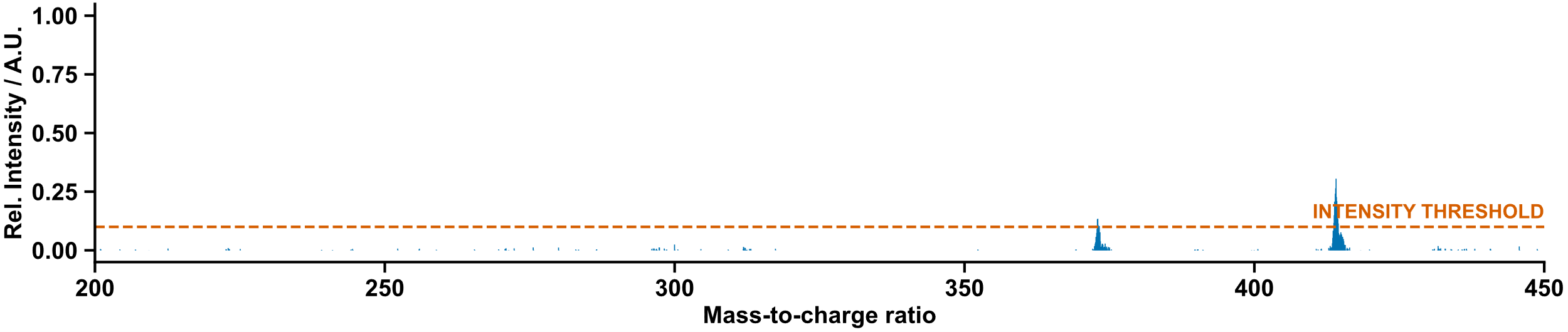
Figure S 40:**UPLC chromatogram of scale-up **7**.


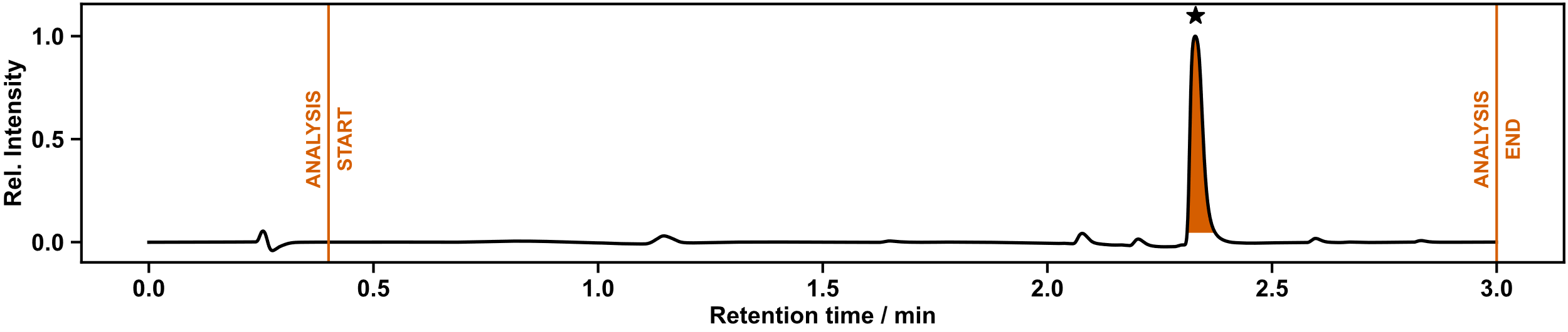


**Figure S 41:** Mass spectrum of screening sample **7**.

### Scale-up (8)


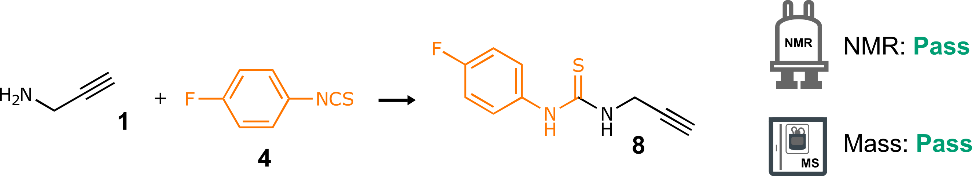


**Scheme S 10:** Synthesis of thiourea **8** from amine **1** and isothiocyanate **4**. Reaction conditions: CH_2_Cl_2_, r.t., 12 h.

| *NMR*: Pass | *MS*: Pass |
| --- | --- |
| *Peaks matching to reference* | *retention time*: 1.54 min  *LC area*: 100% |
|  | *ion observed*: [**8**+H]^+^  *m/z expected*: 209.05  *m/z measured*: 209.05 |

**Table S 9:** Summary of automated decision-maker outcomes for ^1^H NMR spectroscopy and ULPC-MS spectrometry.


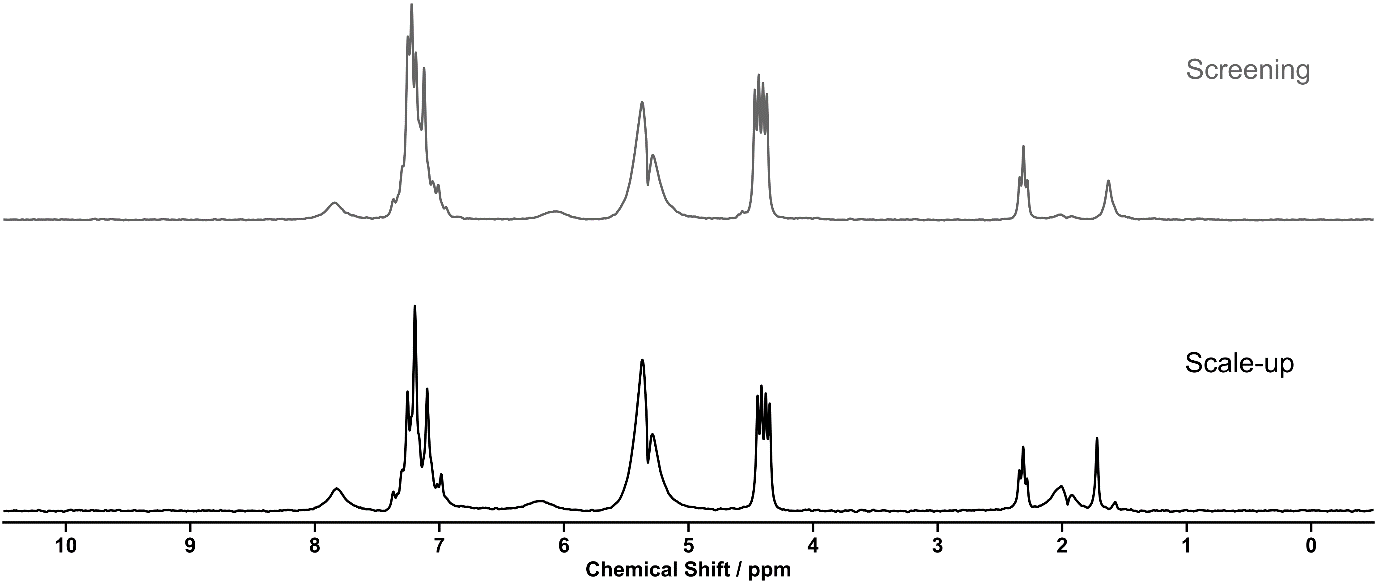


**Figure S 42:** ^1^H NMR spectrum (80 MHz, CH_2_Cl_2_) of screening **8** (top), scale-up **8** (bottom).


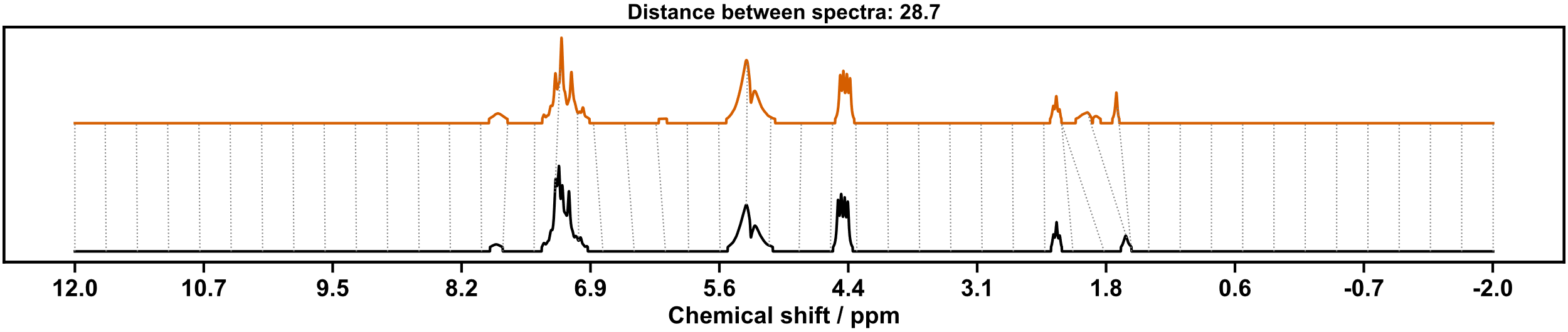


**Figure S 43:** Dynamic time warp comparison of screening **8** (top) with scale-up **8**.


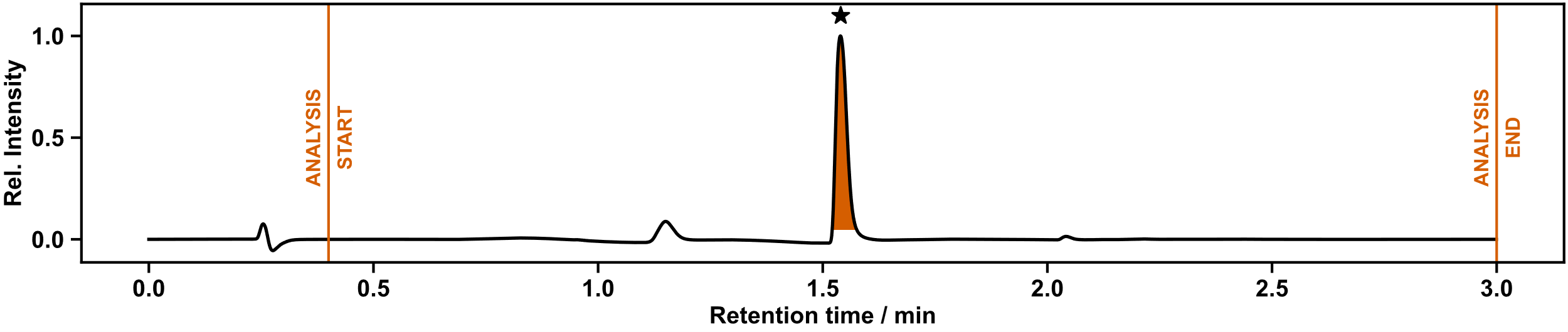


**Figure S 44:** UPLC chromatogram of scale-up **8**.


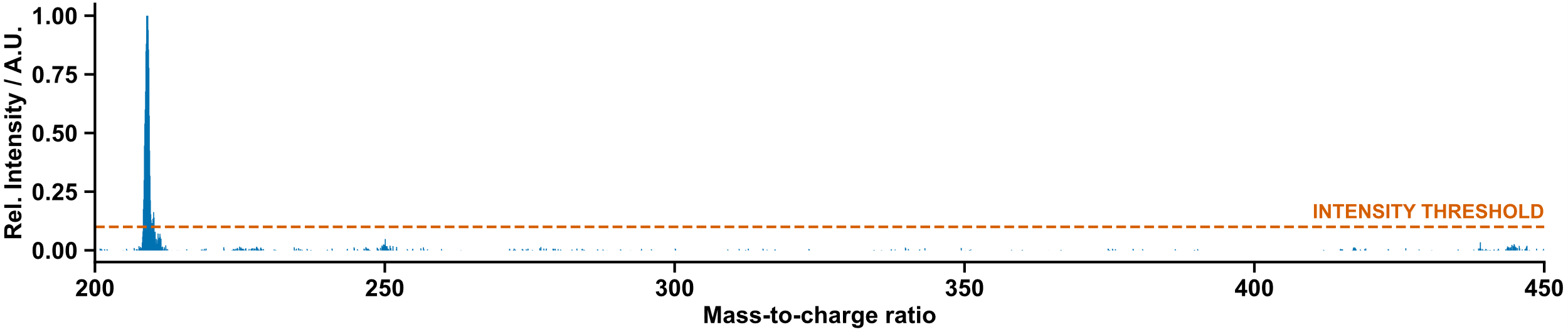


**Figure S 45:** Mass spectrum of screening sample **8**.

### Scale-up (9)


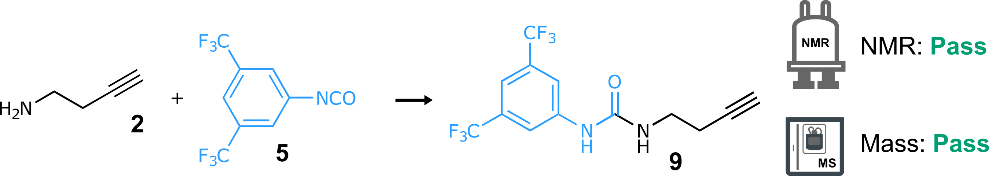


**Scheme S 11:** Synthesis of urea **9** from amine **2** and isocyanate **5**. Reaction conditions: CH_2_Cl_2_, r.t., 12 h.

| *NMR*: Pass | *MS*: Pass |
| --- | --- |
| *Peaks matching reference* | *retention time*: 2.09 min  *LC area*: 100% |
|  | *ion observed*: [**9**+H]^+^  *m/z expected*: 325.08  *m/z measured*: 325.12  *ion observed*: [**9**+CH_3_CN+H]^+^  *m/z expected*: 365.96  *m/z measured*: 366.09 |

**Table S 10:** Summary of automated decision-maker outcomes for ^1^H NMR spectroscopy and ULPC-MS spectrometry.
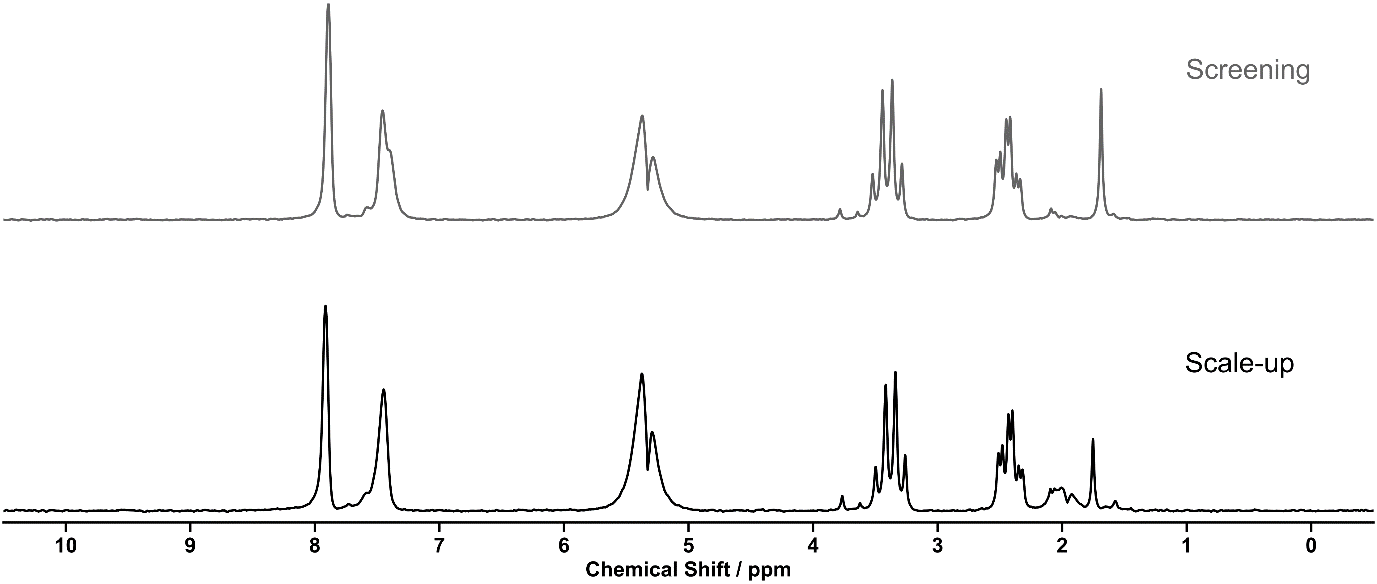


**Figure S 46:** ^1^H NMR spectrum (80 MHz, CH_2_Cl_2_) of screening **9** (top), scale-up **9** (bottom).


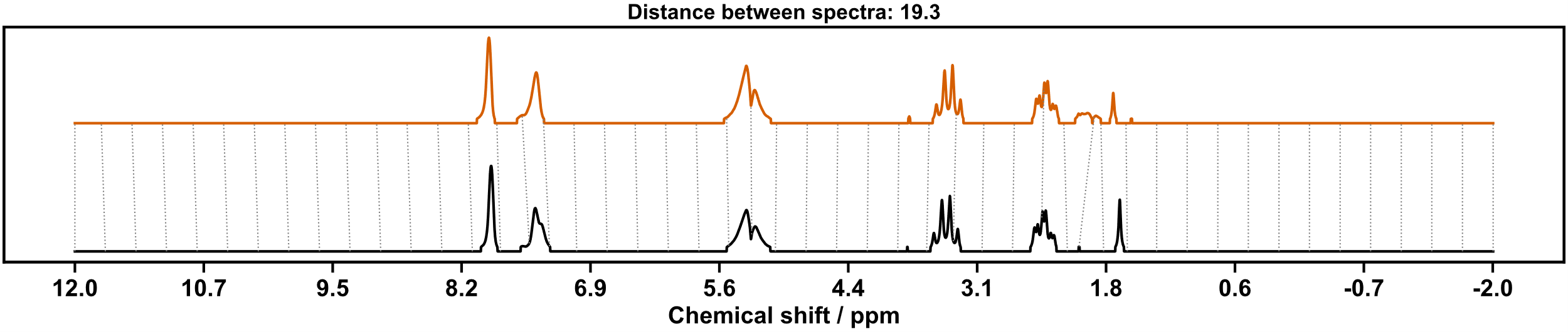


**Figure S 47:** Dynamic time warp comparison of screening **9** (top) with scale-up **9**.


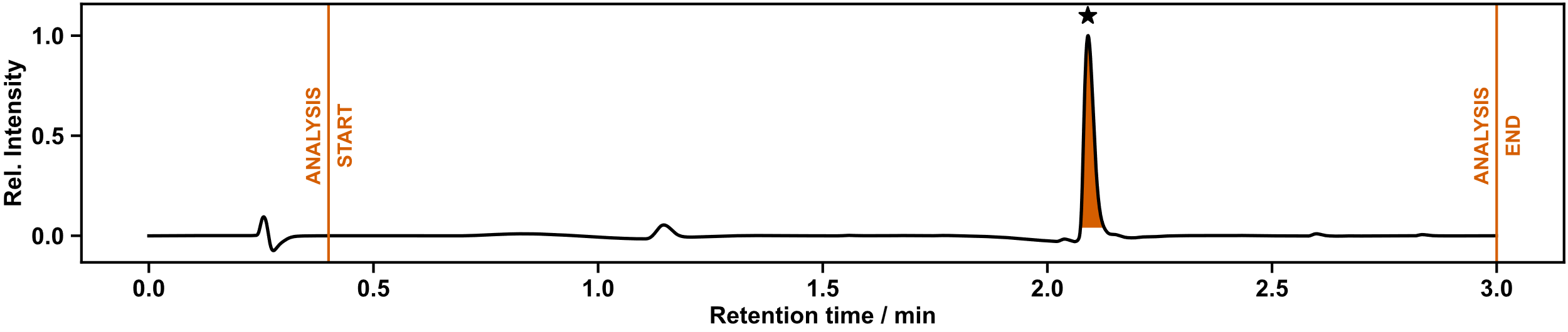


**Figure S 48:** UPLC chromatogram of scale-up **9**.


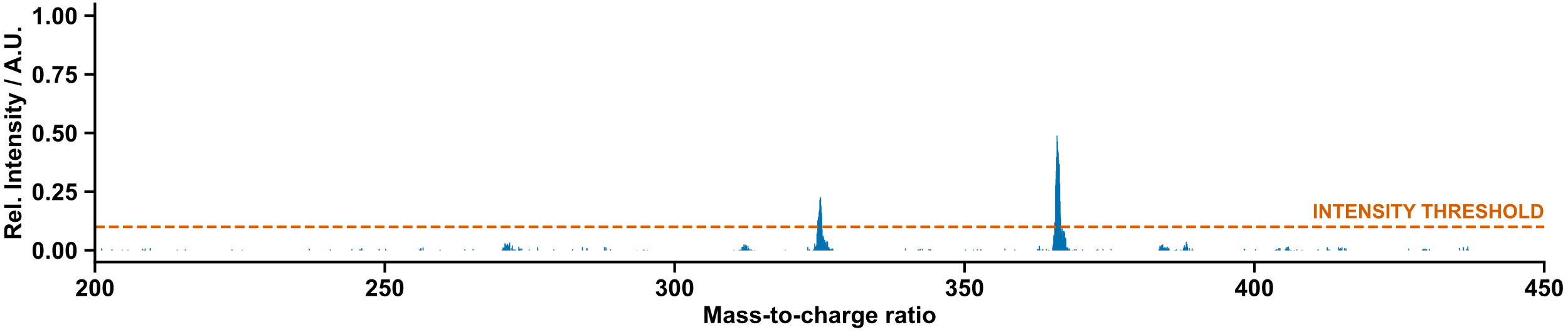


**Figure S 49:** Mass spectrum of screening sample **9**.

### Scale-up (10)


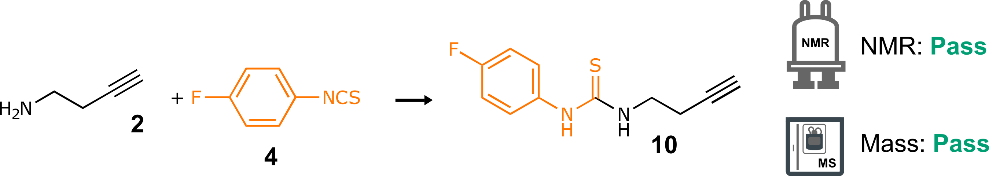


**Scheme S 12:** Synthesis of thiourea **10** from amine **2** and isothiocyanate **4**. Reaction conditions: CH_2_Cl_2_, r.t., 12 h.

| *NMR*: Pass | *MS*: Pass |
| --- | --- |
| *Peaks matching reference* | *retention time*: 1.64 min  *LC area*: 81% |
|  | *ion observed*: [**10**+H]^+^  *m/z expected*: 223.07  *m/z measured*: 222.98 |

**Table S 11:** Summary of automated decision-maker outcomes for ^1^H NMR spectroscopy and ULPC-MS spectrometry.


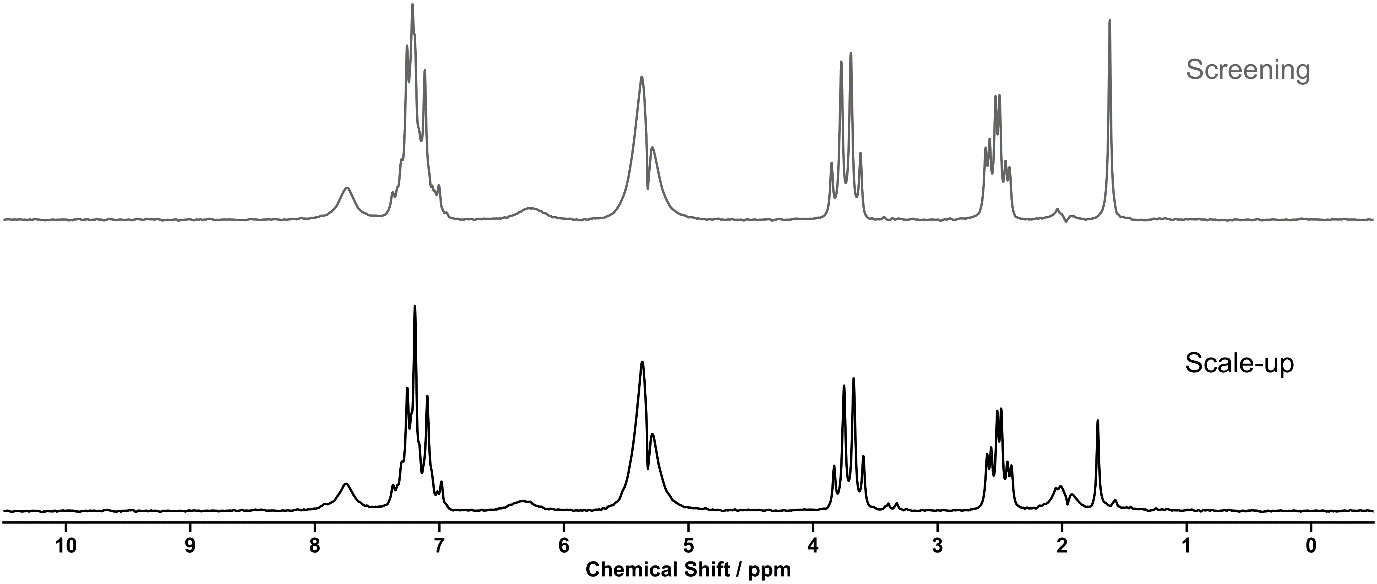


**Figure S 50:** ^1^H NMR spectrum (80 MHz, CH_2_Cl_2_) of screening **10** (top), scale-up **10** (bottom).


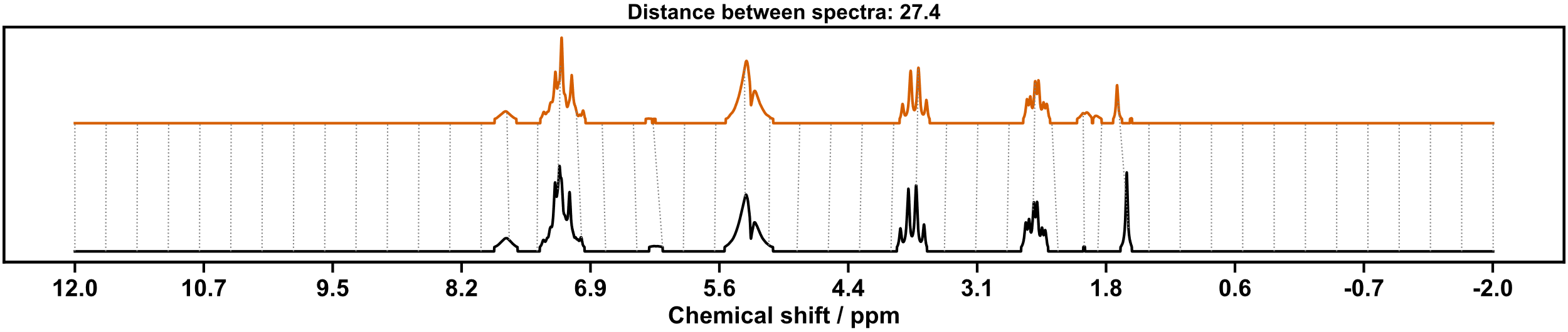


**Figure S 51:** Dynamic time warp comparison of screening **10** (top) with scale-up **10**.


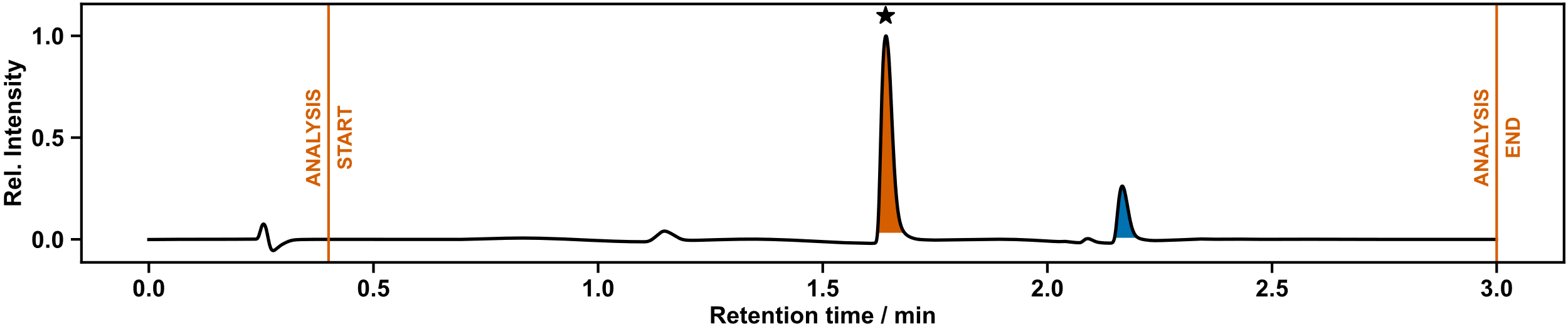


**Figure S 52:** UPLC chromatogram of scale-up **10**.


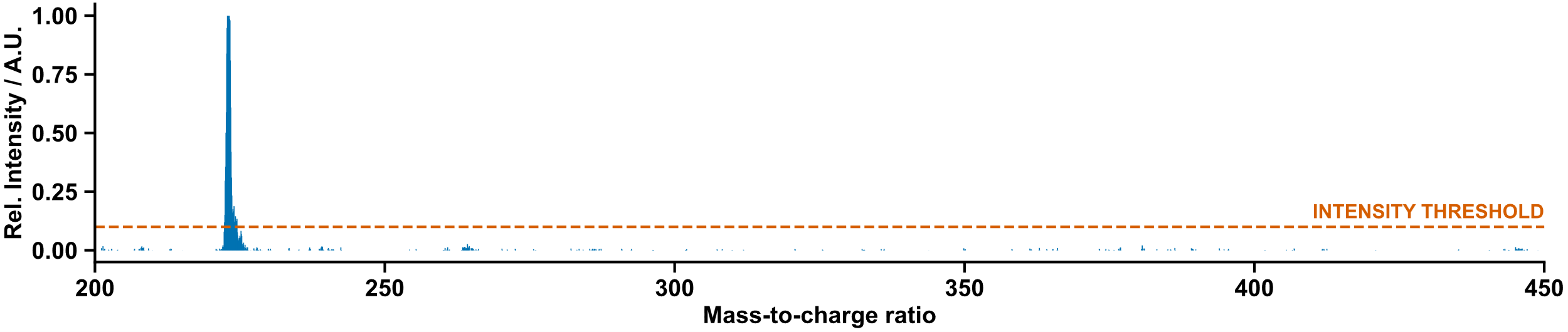


**Figure S 53:** Mass spectrum of screening sample **10**.

## Sonogashira Cross-Coupling

20 mL ISynth vials were charged with pre-weighed quantities of NaPdCl_4_, cataCXium Fsulf ligand, K_2_CO_3_, and 2-bromopyridine and placed inside the Chemspeed platform, along with stock bottles of degassed water and IPA. Water was dispensed to prepare stock solutions of K_2_CO_3_ (190 mM). IPA was dispensed to prepare a stock solution of 2-bromopyridine (209 mM). Water and IPA (1:1) were added to prepare a solution of cataCXium Fsulf (19 mM). The vials were shaken to ensure dissolution. The stock solution of cataCXium Fsulf was transferred to the vial containing NaPdCl_4_ to generate a soluble palladium pre-catalyst, and the vials were shaken again.

Aliquots of the palladium pre-catalyst (1.5 mL), K_2_CO_3_ (1.5 mL), and 2-bromopyridine (1.5 mL) stock solutions were dispensed to vials containing thioureas prepared in scale-up step. Once the reagents were loaded IPA (3 mL) and water (1.5 mL) were also added to each reaction vial. The reactions were shaken at room-temperature for 14 hours and allow to cool to ambient temperature. CH_2_Cl_2_ (6 mL) was added to each of the samples, shaken, and allowed to settle for 30 minutes to allow aqueous and organic layers to separate. Aliquots of the organic layer, which was selectively sampled by selecting needle height, were taken for UPLC and NMR measurements.

Following the completion of the UPLC and NMR measurements, successful reaction mixtures were removed from the Chemspeed, an aliquot of these samples was extracted with CH_2_Cl_2_ (~10 mL x 3), and this aliquot was purified by automated flash column chromatography (Biotage Isolera, prepacked silica normal phase HC-D cartridges, UV-triggered fraction collection) for characterization. Solvents used for purification are listed along with each product.

## (Thio)urea Sonogashira Diversification Results

### Diversification (13)

#### Outcome of Automation

**
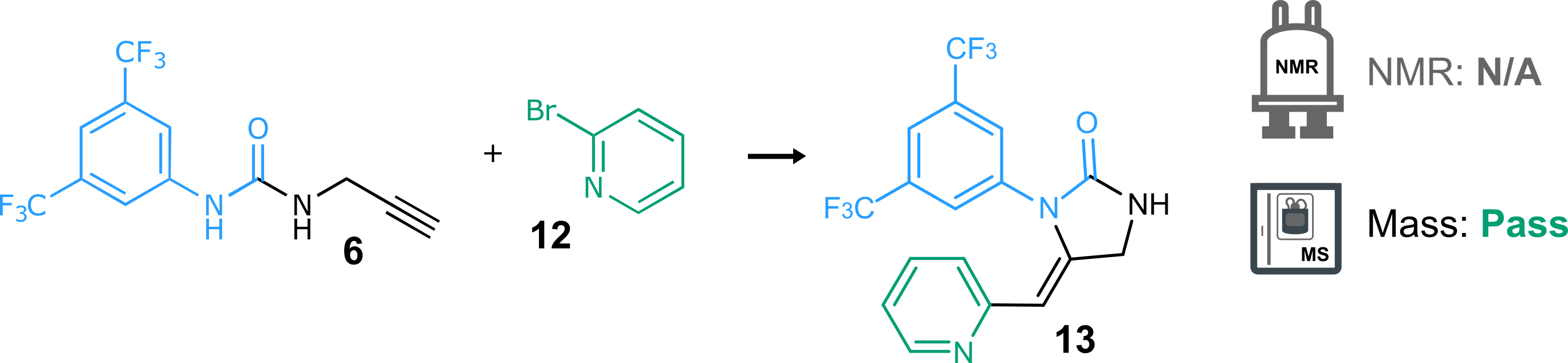
**

**Scheme S 13:** Synthesis of **13** from urea **6** and pyridine **12**. Reaction conditions: K_2_CO_3_ (1.6 eq.), NaPdCl_4_ (10 mol%)_,_ cataCXium Fsulf (10 mol%), H_2_O:IPA (1:1), r.t., 14 h.

| *NMR*: N/A | *MS*: Pass |
| --- | --- |
| *Recorded solely for reference* | *retention time*: 1.45 min  *LC area*: 9% |
|  | *ion observed*: [**13**+H]^+^  *m/z expected*: 388.09  *m/z measured*: 388.00 |

**Table S 12:** Summary of automated decision-maker outcome for ULPC-MS spectrometry.


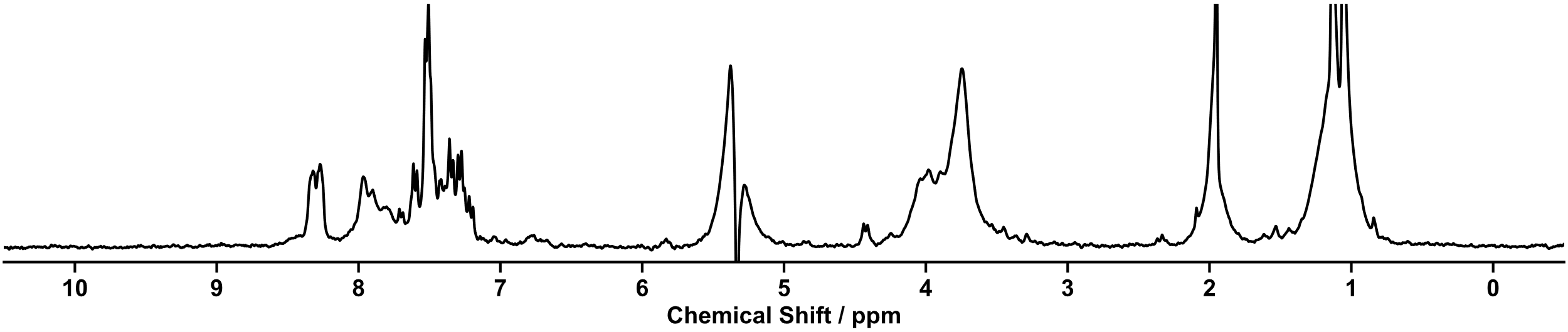


**Figure S 54:** ^1^H NMR spectrum (80 MHz) spectrum of diversification **13**.

**
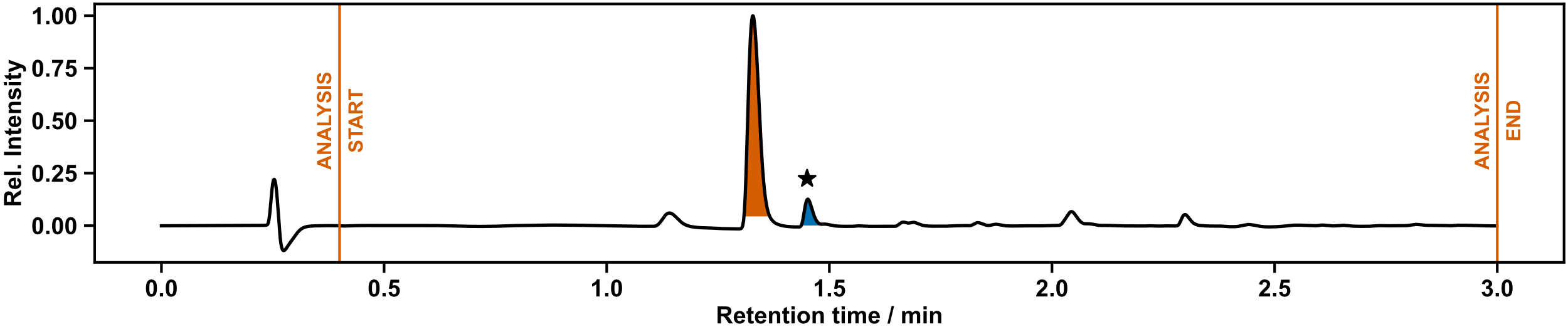
**

**Figure S 55:** UPLC chromatogram of diversification **13**.

**
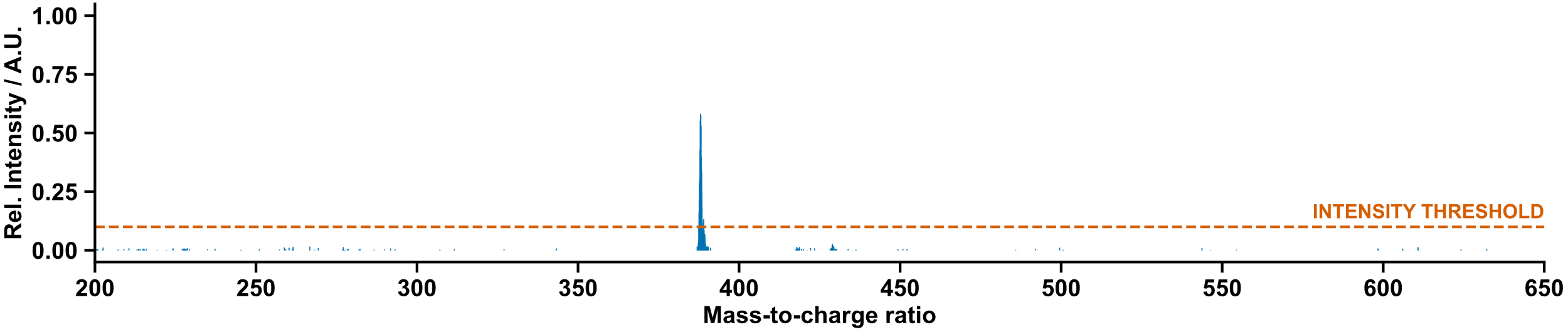
**

**Figure S 56:** Mass spectrum of diversification **13**.

#### Manual Characterization

Purified by flash column chromatography (CH_2_Cl_2_/MeOH gradient) to yield the product as a yellow oil. Some co-eluting impurities were inseparable by chromatography.

**^1^H NMR** (400 MHz, Acetone-*d_6_*) δ 7.85 – 7.79 (m, 1H, H^15^), 7.67 – 7.59 (m, 3H, H^4+5^), 7.38 (td, *J* = 7.7, 1.9 Hz, 1H, H^13^), 7.00 – 6.95 (m, 1H, H^14^), 6.79 (ddd, *J* = 7.6, 4.8, 1.1 Hz, 1H, H^12^), 5.92 (t, *J* = 2.2 Hz, 1H, H^10^), 4.55 – 4.49 (m, 2H, H^8^).

**^13^C NMR** (101 MHz, Acetone-*d_6_*) δ 159.13 (C^7^), 154.28 (C^11^), 148.62 (C^15^), 140.72 (C^6^), 138.63 (C^9^), 135.98 (C^13^), 131.27 (q, *J* = 33.4 Hz, C^3^), 126.71 – 126.54 (m, C^5^), 124.25 (C^14^), 124.17 (q, *J* = 272.1 Hz, C^2^), 120.53 (C^12^), 119.06 – 118.94 (m, C^4^), 101.22 (C^10^), 46.01 (C^11^).

**^19^F NMR** (376 MHz, Acetone-*d_6_*) δ -63.42 (F^1^).

**HRMS** calculated for C_17_H_12_F_6_N_3_O+: 388.0879; found: 388.0884.

***N.B.*:** Due to the very low yield of this reaction, and difficulty in purification – we manually resynthesized compound **13** with heating at 60 °C using the same conditions to improve the yield of the reaction for unambiguous characterization. Spectroscopic data of compound **13** from the heated reaction was consistent with the values obtained from the on-Chemspeed run. ^13^C NMR data has been reported for the resynthesized sample.


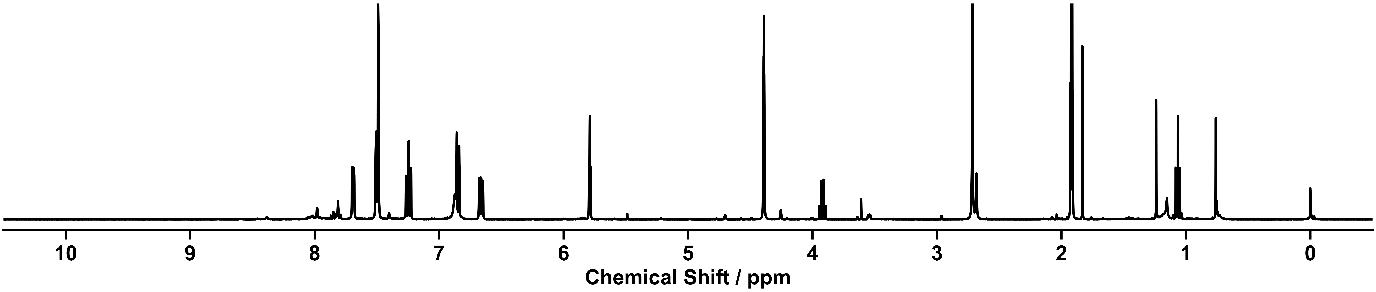


**Figure S 57:** ^1^H NMR spectrum (400 MHz, Acetone-*d_6_*) of diversification **13**.


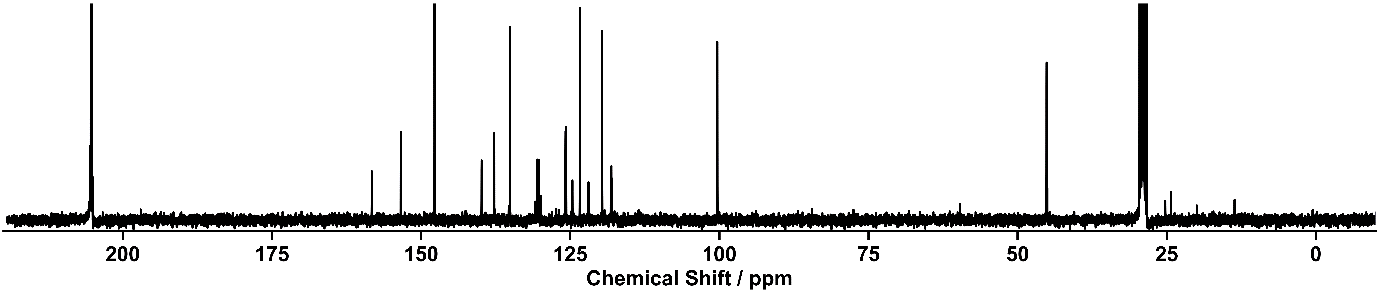


**Figure S 58**: 13C NMR spectrum (101 MHz, Acetone-*d_6_*) of diversification **13**.

### Diversification (14)

#### Outcome of Automation


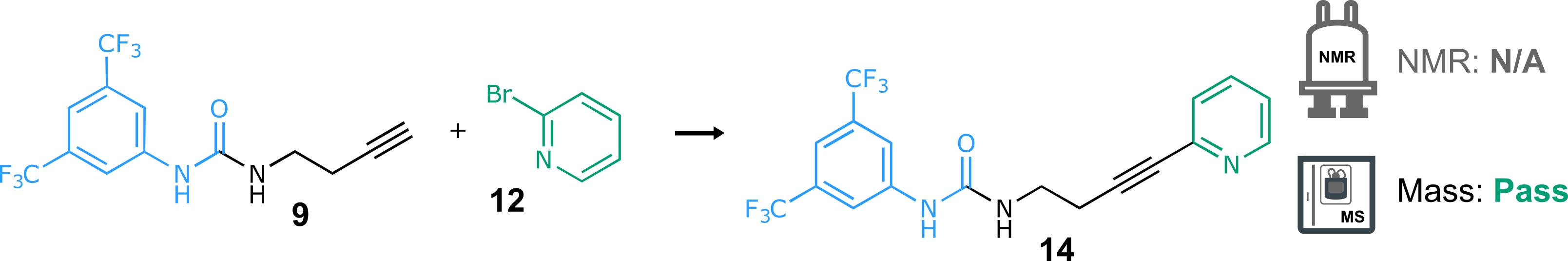


**Scheme S 14:** Synthesis of **14** from urea **6** and pyridine **12**. Reaction conditions: K_2_CO_3_ (1.6 eq.), NaPdCl_4_ (10 mol%)_,_ cataCXium Fsulf (10 mol%), H_2_O:IPA (1:1), r.t., 14 h.

| *NMR*: N/A | *MS*: Pass |
| --- | --- |
| *Recorded solely for reference* | *retention time*: 1.33 min  *LC area*: 36% |
|  | *ion observed*: [**14**+H]^+^  *m/z expected*: 402.10  *m/z measured*: 402.12 |

**Table S 13:** Summary of automated decision-maker outcome for ULPC-MS spectrometry.


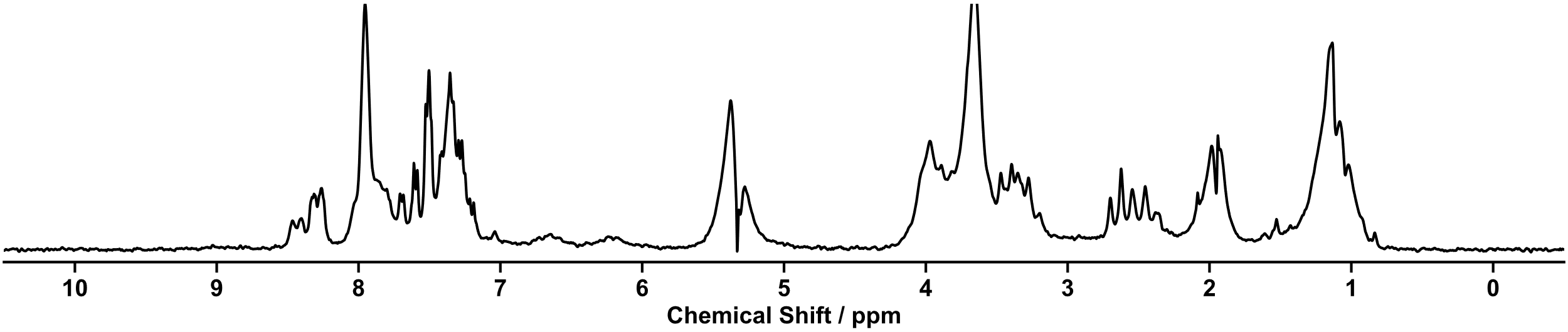


**Figure S 59:** ^1^H NMR spectrum (80 MHz) spectrum of diversification **14**.


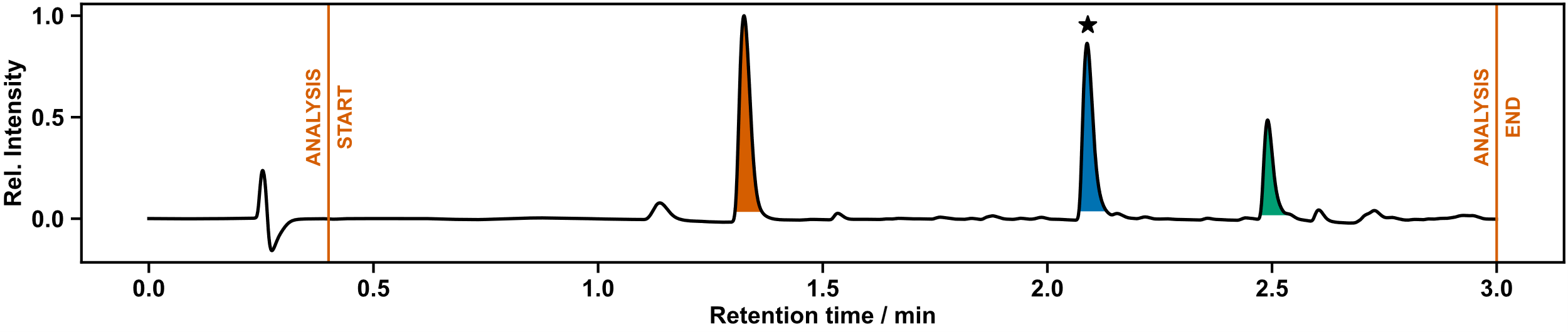


**Figure S 60:** UPLC chromatogram of diversification **14**.


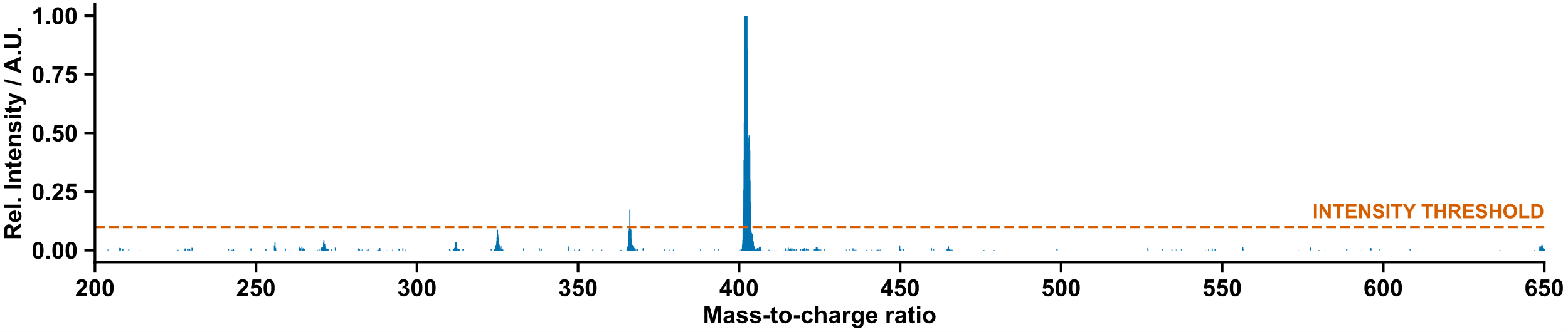


**Figure S 61:** Mass spectrum of diversification **14**.

#### Manual Characterization

Purified by flash column chromatography (CH_2_Cl_2_/MeOH gradient) to yield the product as a yellow oil.

**^1^H NMR** (400 MHz, Acetone-*d_6_*) δ 8.77 (s, 1H, H^7^), 8.56 – 8.50 (m, 1H, H^18^), 8.16 (s, 2H, H^5^), 7.74 (td, *J* = 7.7, 1.8 Hz, 1H, H^16^), 7.54 – 7.51 (m, 1H, H^4^), 7.44 (dt, *J* = 7.8, 1.1 Hz, 1H, H^15^), 7.31 (ddd, *J* = 7.6, 4.9, 1.2 Hz, 1H, H^17^), 6.48 – 6.43 (m, 1H, H^9^), 3.56 – 3.46 (m, 2H, H^10^), 2.71 (t, *J* = 6.7 Hz, 2H, H^11^).

**^13^C NMR** (101 MHz, Acetone-*d_6_*) δ 155.65 (C^8^), 150.69 (C^18^), 144.47 (C^14^), 143.56 (C^6^), 137.11 (C^16^), 132.37 (q, *J* = 32.9 Hz, C^3^), 127.89 (C^15^), 124.52 (q, *J* = 271.9 Hz, C^2^), 123.64 (C^17^), 118.76 – 118.24 (m, C^5^), 115.06 – 114.53 (m, C^4^), 88.36 (C^12^), 82.48 (C^13^), 39.56 (C^10^), 21.16 (C^11^).

**^19^F NMR** (376 MHz, Acetone-*d_6_*) δ -63.65 (F^1^).

**HRMS** calculated for C_18_H_14_F_6_N_3_O+: 402.1036; found: 402.1040.


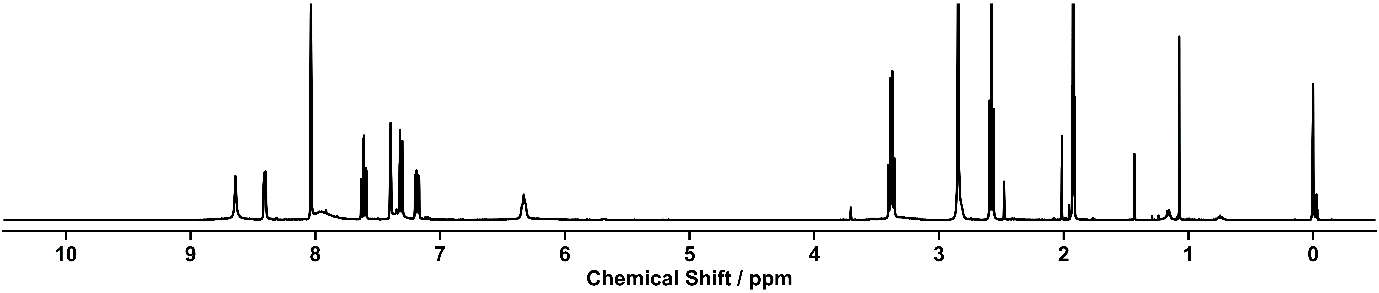


**Figure S 62:** ^1^H NMR spectrum (400 MHz, Acetone-*d_6_*) of diversification **14**.


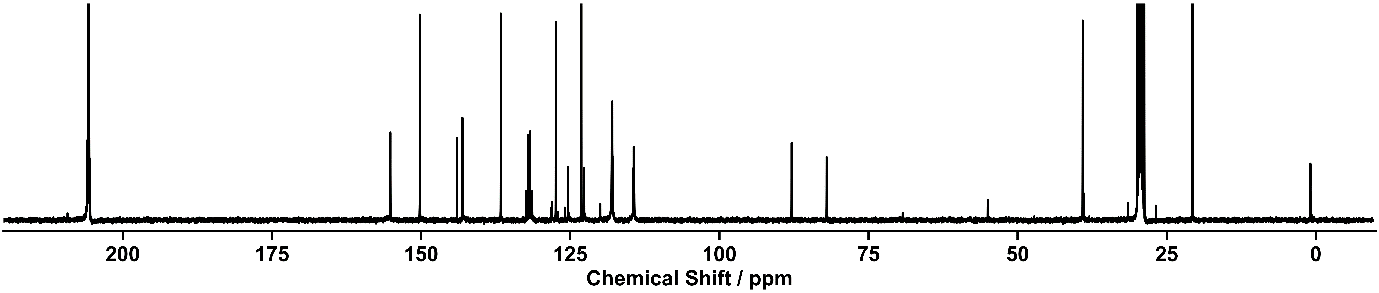


**Figure S 63**: 13C NMR spectrum (101 MHz, Acetone-*d_6_*) of diversification **14**.

### Diversification (15)

#### Outcome of Automation


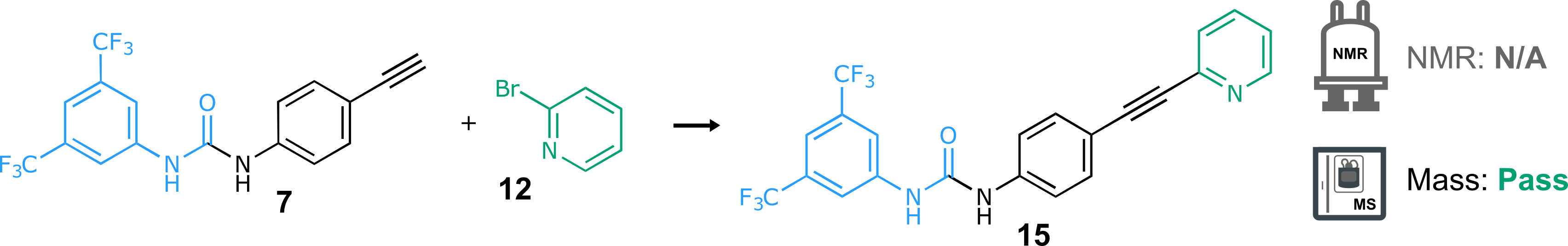


**Scheme S 15:** Synthesis of **15** from urea **6** and pyridine **12**. Reaction conditions: K_2_CO_3_ (1.6 eq.), NaPdCl_4_ (10 mol%)_,_ cataCXium Fsulf (10 mol%), H_2_O:IPA (1:1), r.t., 14 h.

| *NMR*: N/A | *MS*: Pass |
| --- | --- |
| *Recorded solely for reference* | *retention time*: 2.30 min  *LC area*: 50% |
|  | *ion observed*: [**15**+H]^+^  *m/z expected*: 450.10  *m/z measured*: 450.06  *ion observed*: [**15**+CH_3_CN+H]^+^  *m/z expected*: 491.12  *m/z measured*: 490.91 |

**Table S 14:** Summary of automated decision-maker outcome for ULPC-MS spectrometry.


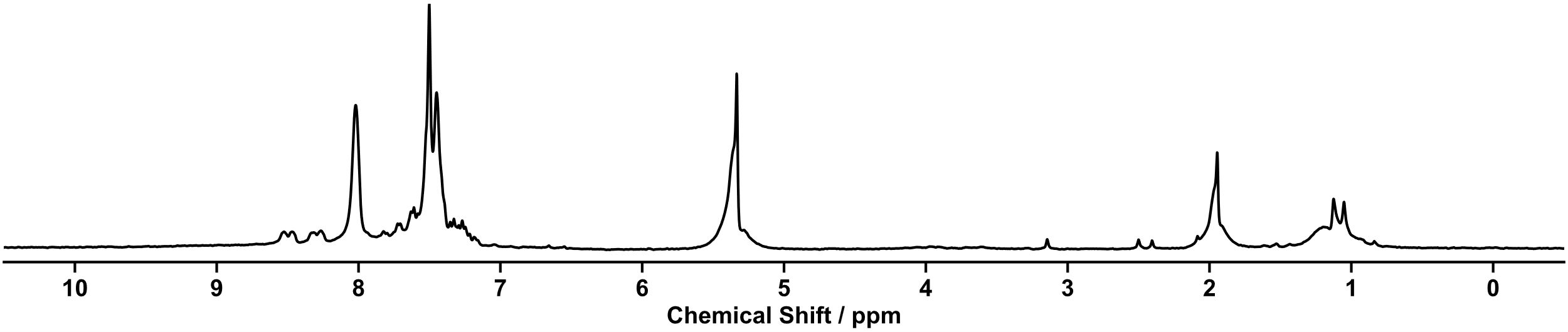


**Figure S 64:** ^1^H NMR spectrum (80 MHz) spectrum of screening **15**.


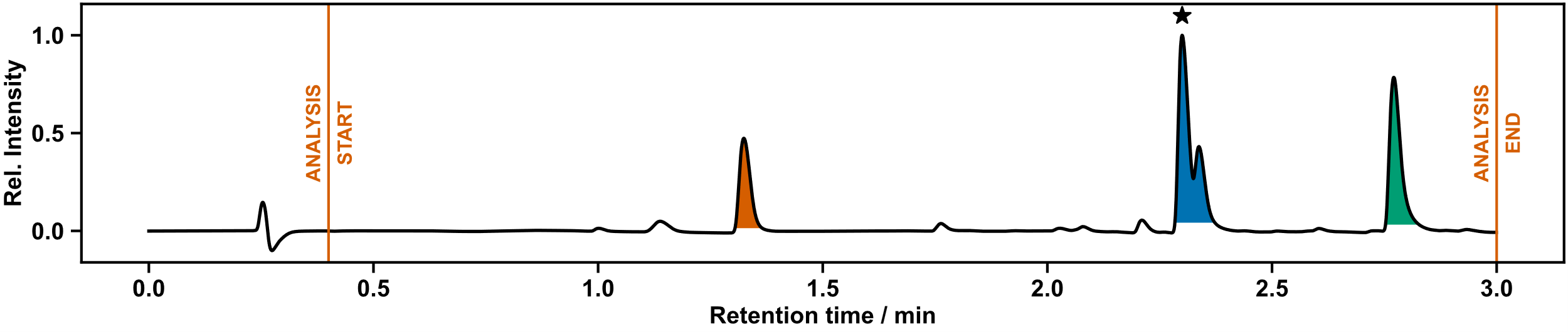


**Figure S 65:** UPLC chromatogram of diversification **15**.


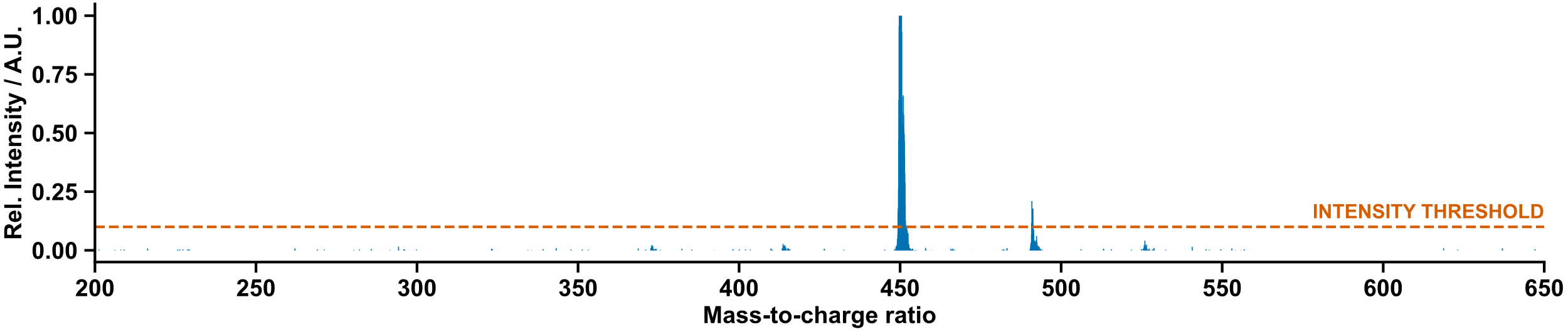


**Figure S 66:** Mass spectrum of diversification **15**.

#### Manual characterization

Purified by column chromatography (CH_2_Cl_2_/Acetone gradient) to yield the product as a brown solid.

**^1^H NMR** (400 MHz, DMSO-*d*_6_) δ 9.50 (s, 1H, H^7^), 9.29 (s, 1H, H^8^), 8.61 – 8.57 (m, 1H, H^20^), 8.14 (s, 2H, H^5^), 7.83 (td, *J* = 7.7, 1.8 Hz, 1H, H^18^), 7.65 (s, 1H, H^4^), 7.63 – 7.51 (m, 5H, H^11+12+17^), 7.42 – 7.36 (m, 1H, H^19^)

**^13^C NMR** (101 MHz, DMSO-*d*_6_) δ 152.70 (C^8^), 150.57 (C^20^), 143.04 (C^16^), 142.10 (C^6^), 140.72 (C^10^), 137.21 (C^17^), 133.01 (C^11 or 12^), 131.20 (q, *J* = 32.6 Hz, C^3^), 127.56 (C^17^), 123.77 (q, *J* = 272.7 Hz, C^2^), 123.71 (C^19^), 119.15 (C^11 or 12^), 118.63 (C^5^), 115.28 (C^13^), 115.17 – 114.97 (m, C^4^), 89.22 (C^15^), 88.71 (C^14^).

**HRMS** calculated for C_22_H_14_F_6_N_3_O+: 450.1036; found: 450.1043.


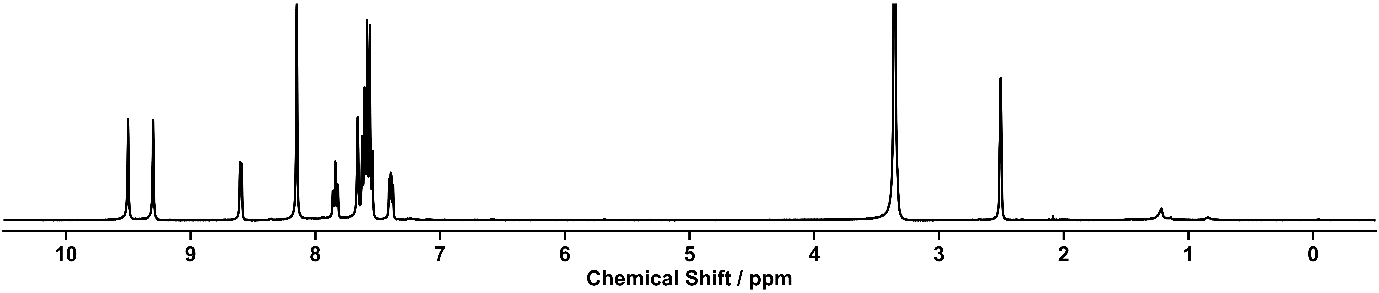


**Figure S 67:** ^1^H NMR spectrum (400 MHz, DMSO-*d*_6_) of diversification **15**.


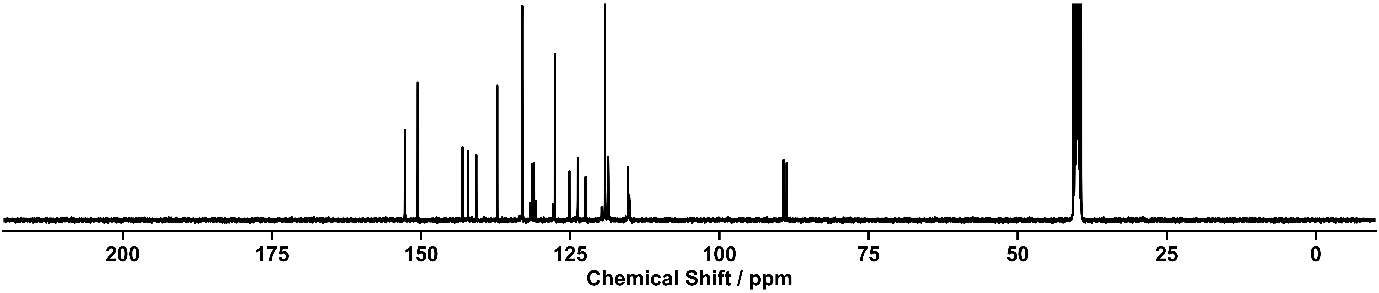


**Figure S 68**: ^13^C NMR spectrum (101 MHz, DMSO-*d*_6_) of diversification **15**.

### Diversification (16)


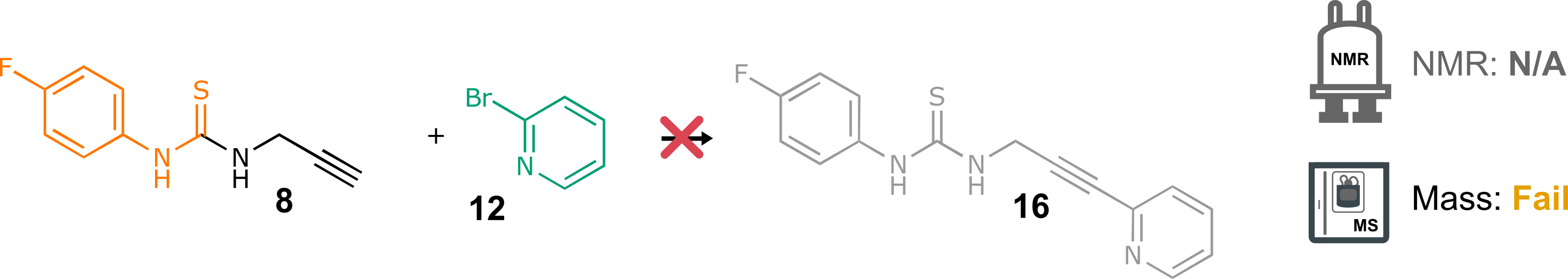


**Scheme S 16:** Attempted synthesis of **16** from thiourea **8** and pyridine **12**. Reaction conditions: K_2_CO_3_ (1.6 eq.), NaPdCl_4_ (10 mol%)_,_ cataCXium Fsulf (10 mol%), H_2_O:IPA (1:1), r.t., 14 h.

| *NMR*: N/A | *MS*: Fail |
| --- | --- |
| *Recorded solely for reference* | *retention time*: 1.11 min  *LC area*: 62%  *retention time*: 1.32 min  *LC area*: 33% |
|  | *No matching ions observed* |

**Table S 15:** Summary of automated decision-maker outcome for ULPC-MS spectrometry.


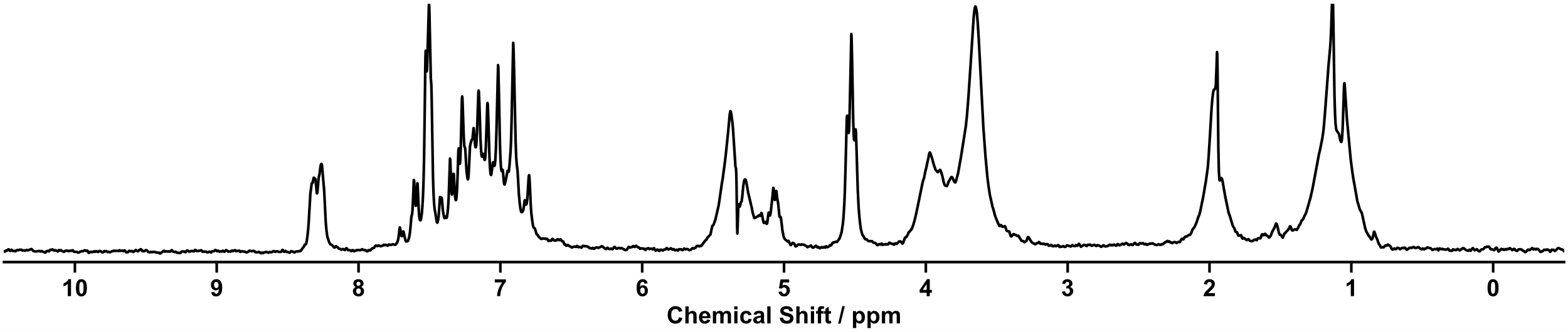


**Figure S 69:** ^1^H NMR spectrum (80 MHz) of screening **16**.


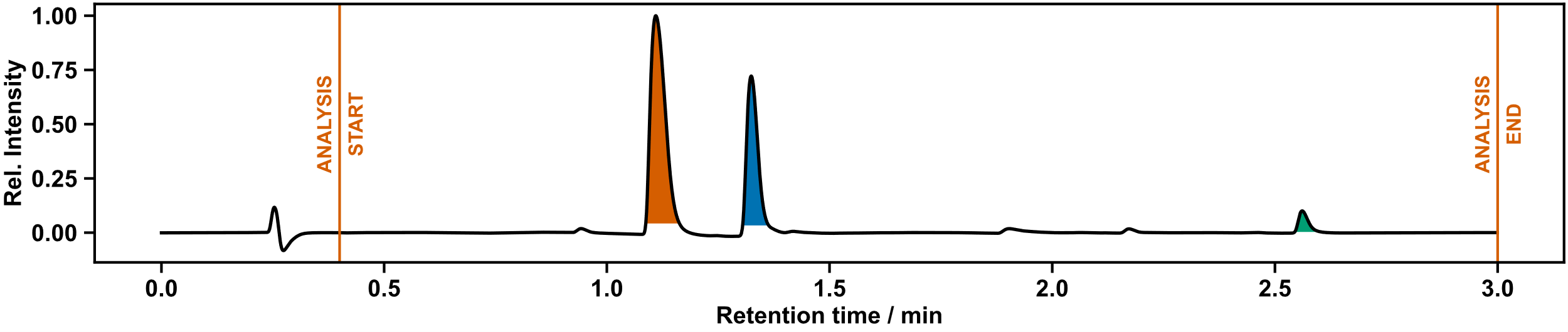


**Figure S 70:** UPLC chromatogram of diversification **16**.

### Diversification (17)


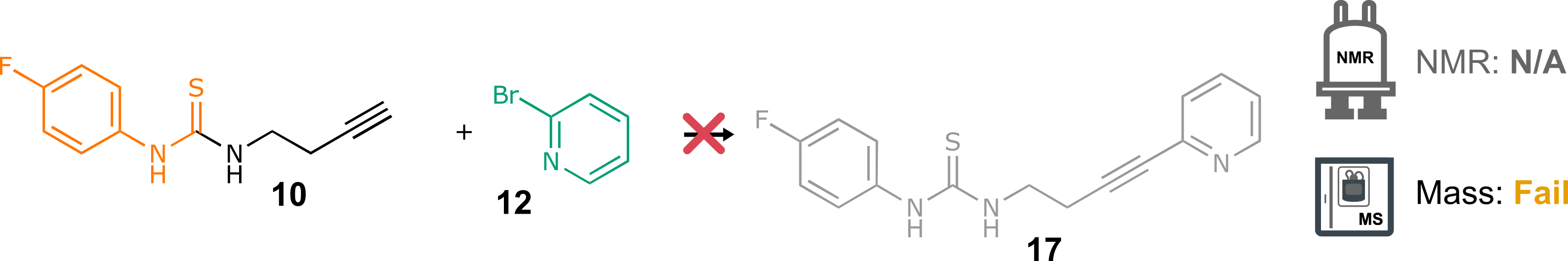


**Scheme S 17:** Attempted synthesis of **17** from thiourea **10** and pyridine **12**. Reaction conditions: K_2_CO_3_ (1.6 eq.), NaPdCl_4_ (10 mol%)_,_ cataCXium Fsulf (10 mol%), H_2_O:IPA (1:1), r.t., 14 h.

| *NMR*: N/A | *MS*: Fail |
| --- | --- |
| *Recorded solely for reference* | *retention time*: 1.32 min  *LC area*: 40%  *retention time*: 1.64 min  *LC area*: 54% |
|  | *No matching ions observed* |

**Table S 16:** Summary of automated decision-maker outcome for ULPC-MS spectrometry.


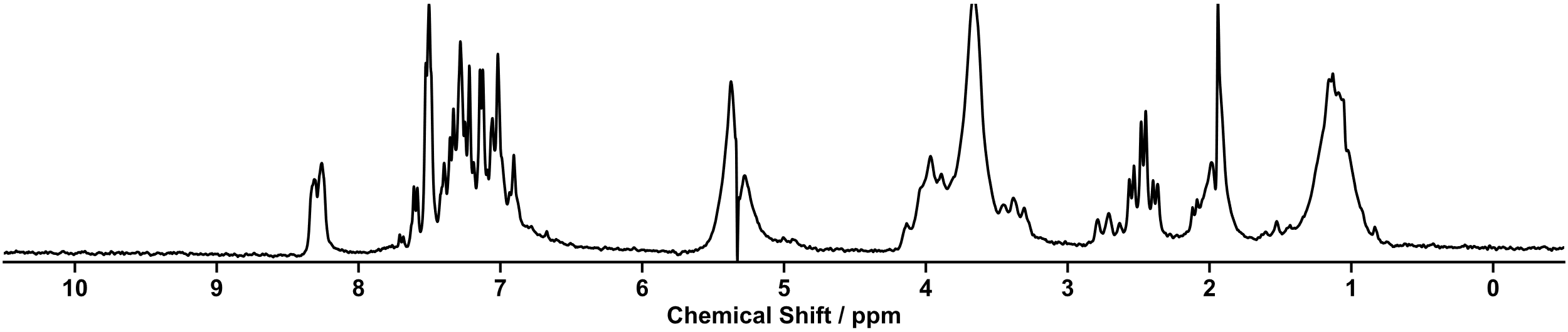


**Figure S 71:** ^1^H NMR spectrum (80 MHz) of screening **17**.


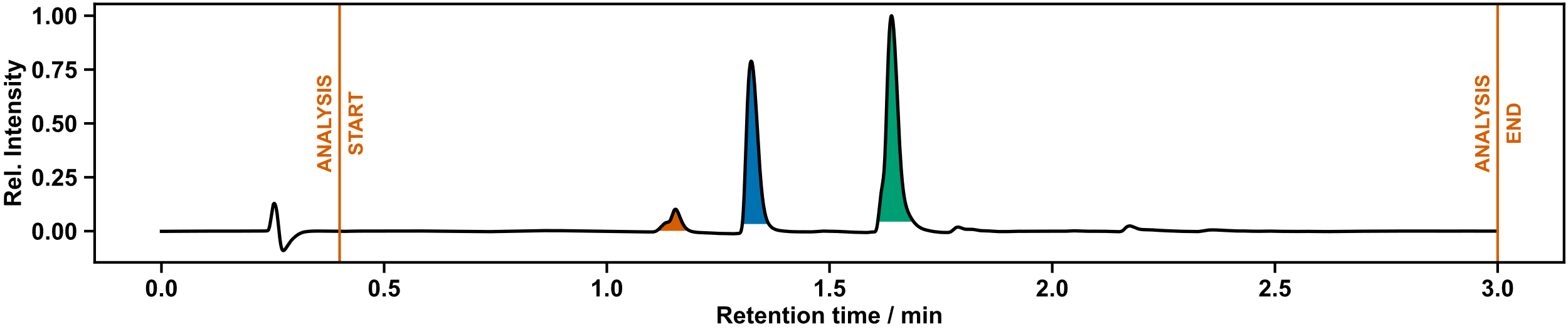


**Figure S 72:** UPLC chromatogram of diversification **17**.

## CuAAc Reactions

20 mL ISynth vials were charged with solid copper sulphate and sodium ascorbate. Water was dispensed volumetrically to the ISynth vials containing copper sulphate and sodium ascorbate to prepare stock solutions (19 mM and 38 mM, respectively). The vials were shaken using the ISynth reactor block to ensure dissolution, and aliquots (1.5 mL) of both stock solutions were dispensed to the reaction vials. Each reaction vial was diluted with IPA (4.5 mL) and water (1.5 mL). The vials were heated for 14 hours at 60 °C, allowed to cool, and aliquots are taken for LC-MS and NMR as in the screening stage, followed by robotic transport by UPLC-Agent and NMR-Agent respectively.

Following the completion of the UPLC and NMR measurements, aliquots of successful reaction mixtures were removed from the Chemspeed, and directly concentrated under vacuum. The residue was redissolved in acetone (~ 30 mL), sonicated, filtered, and purified by automated flash column chromatography (Biotage Isolera, prepacked silica normal phase HC-D cartridges, UV-triggered fraction collection) for characterization. Solvents used for purification are listed along with each product.

The synthetic conditions were adapted from reported literature procedures.^3–5^

## (Thio)urea CuAAC Diversification Results

### Diversification (19)

#### Outcome of Automation

**
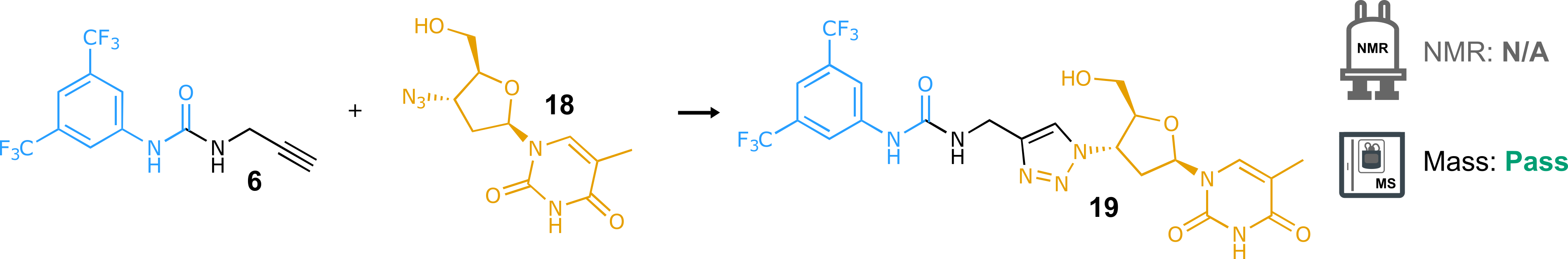
**

**Scheme S 18:** Synthesis of **19** from urea **6** and Zidovudine **18**. Reaction conditions: CuSO_4_ (10 mol%), ascorbic acid (20 mol%)_,_ CH_2_Cl_2_:H_2_O:IPA (2:1:1), 60 °C, 14 h.

| *NMR*: N/A | *MS*: Pass |
| --- | --- |
| *Recorded solely for reference* | *retention time*: 1.78 min  *LC area*: 81% |
|  | *ion observed*: [**19**+H]^+^  *m/z expected*: 578.16  *m/z measured*: 578.07 |

**Table S 17:** Summary of automated decision-maker outcome for ULPC-MS spectrometry.


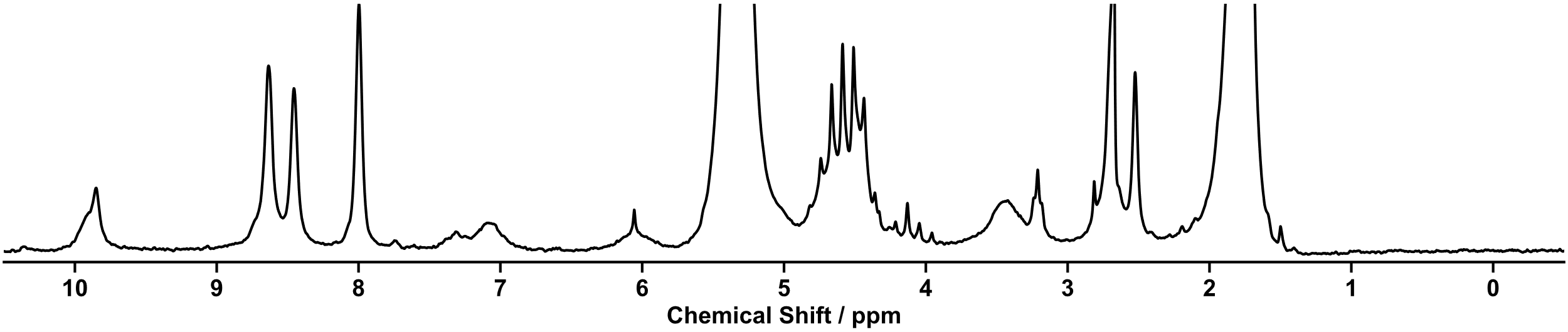


**Figure S 73**: ^1^H NMR spectrum (80 MHz) of screening **19**.

**
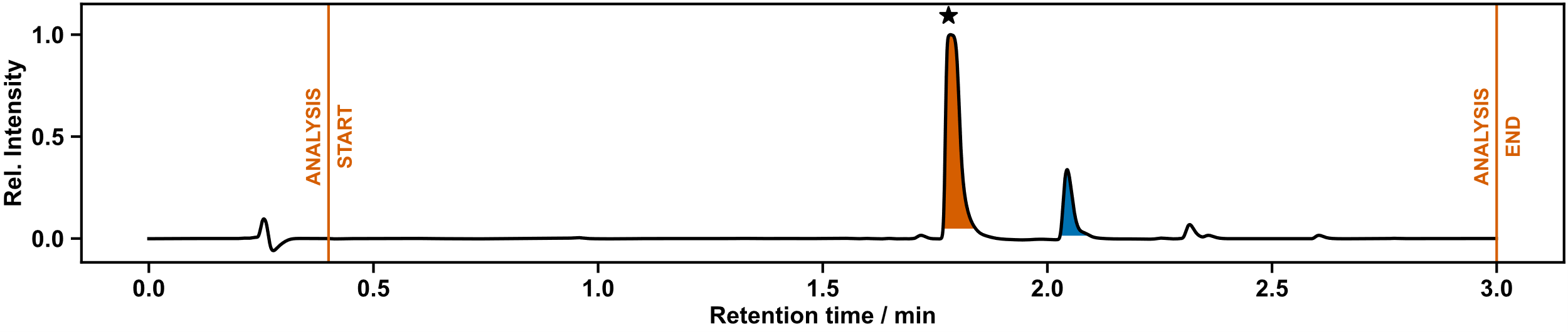
**

**Figure S 74:** UPLC chromatogram of diversification **19**.


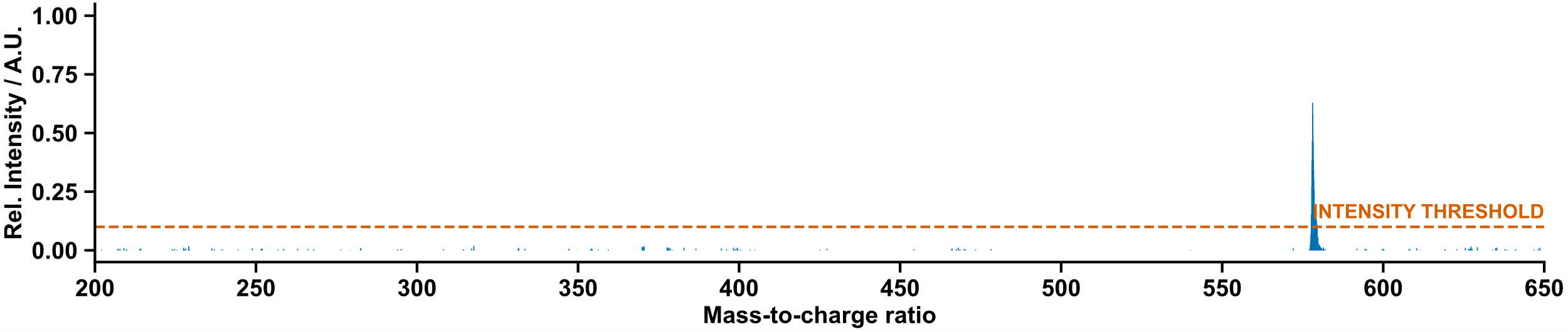


**Figure S 75:** Mass spectrum of diversification **19**.

#### Manual Characterization

Purified by flash column chromatography (CH_2_Cl_2_/Methanol gradient) to yield the product as a white solid.

**^1^H NMR** (400 MHz, CD_3_OD) δ 7.96 (s, 1H, H^10^), 7.92 (s, 2H, H^5^), 7.80 (q, *J* = 1.2 Hz, 1H, H^16^), 7.38 (s, 1H, H^4^), 6.38 (t, *J* = 6.5 Hz, 1H, H^13^), 5.31 (dt, *J* = 8.5, 5.4 Hz, 1H, H^11^), 4.39 (s, 2H, H^8^), 4.26 (dt, *J* = 5.7, 3.1 Hz, 1H, H^12^), 3.79 (dd, *J* = 12.3, 3.0 Hz, 1H, H^14^), 3.66 (dd, *J* = 12.2, 3.2 Hz, 1H, H^14^), 2.85 – 2.75 (m, 1H, H^15^), 2.67 – 2.56 (m, 1H, H^15^), 1.80 (d, *J* = 1.2 Hz, 3H, H^18^).

**^13^C NMR** (126 MHz, CD_3_OD) δ 165.00 (C^19^), 155.82 (C^7^), 150.91 (C^20^), 145.86 (C^9^), 141.92 (C^6^), 136.85 (C^16^), 131.72 (q, *J* = 33.0 Hz, C^3^), 123.43 (q, *J* = 271.9 Hz, C^2^), 122.57 (C^10^), 118.94 – 116.45 (m, C^5^), 115.50 – 112.95 (m, C^4^), 110.30 (C^17^), 85.30 (C^13^), 84.97 (C^12^), 60.75 (C^14^), 59.68 (C^11^), 37.66 (C^15^), 34.73 (C^8^), 11.07 (C^18^).

**^19^F NMR** (376 MHz, CD_3_OD) δ -64.63 (F^1^).


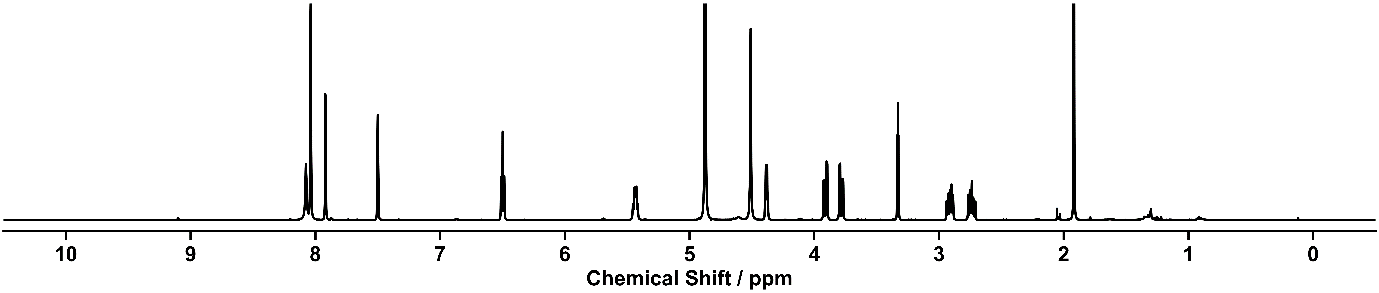
**HRMS** calculated for C_22_H_22_F_6_N_7_O_5_+: 578.1581; found: 578.1593.

**Figure S 76:** ^1^H NMR spectrum (400 MHz, CD_3_OD) of diversification **19**.


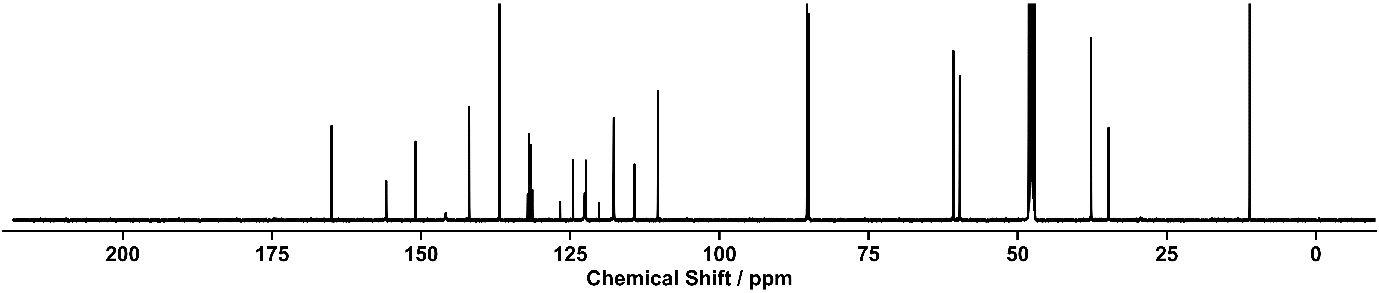


**Figure S 77**: ^13^C NMR spectrum (126 MHz, CD_3_OD) of diversification **19**.

### Diversification (20)

#### Outcome of Automation


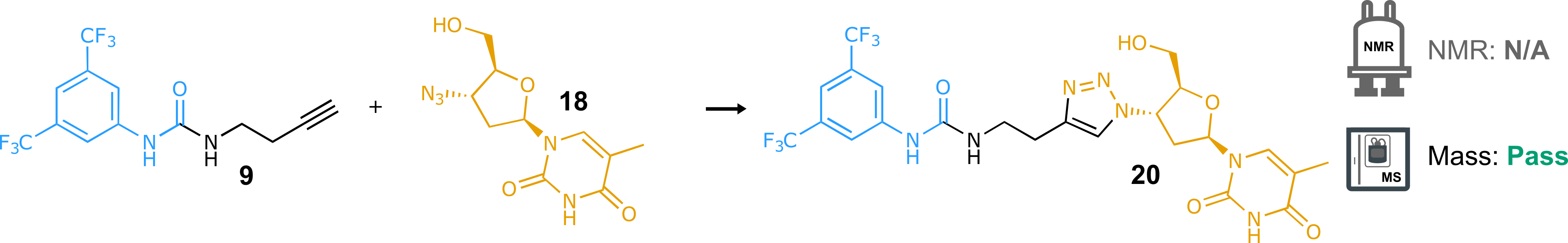


**Scheme S 19:** Synthesis of **20** from urea **9** and Zidovudine **18**. Reaction conditions: CuSO_4_ (10 mol%), ascorbic acid (20 mol%)_,_ CH_2_Cl_2_:H_2_O:IPA (2:1:1), 60 °C, 14 h.

| *NMR*: N/A | *MS*: Pass |
| --- | --- |
| *Recorded solely for reference* | *retention time*: 1.79 min  *LC area*: 83% |
|  | *ion observed*: [**20**+H]^+^  *m/z expected*: 592.18  *m/z measured*: 592.20 |

**Table S 18:** Summary of automated decision-maker outcome for ULPC-MS spectrometry.


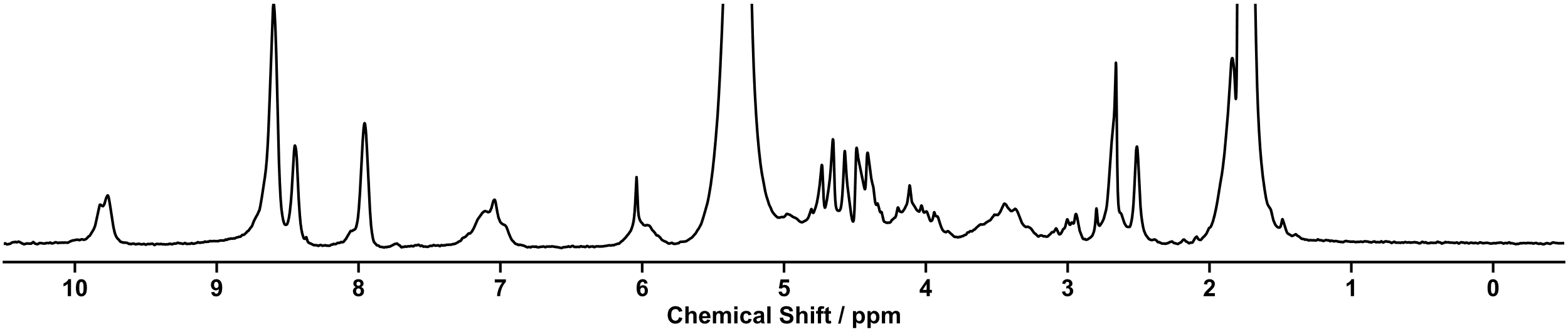


**Figure S 78:** ^1^H NMR spectrum (80 MHz) of screening **20**.


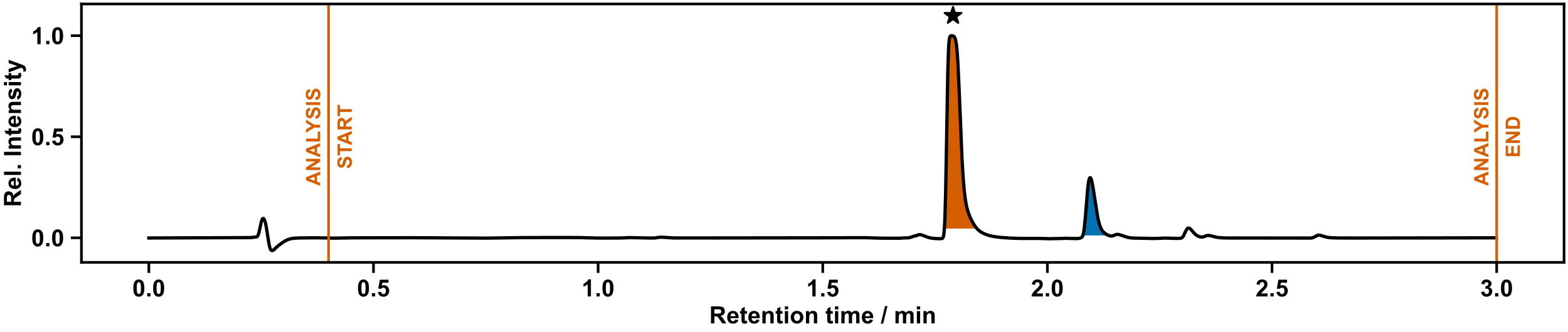


**Figure S 79:** UPLC chromatogram of diversification **20**.


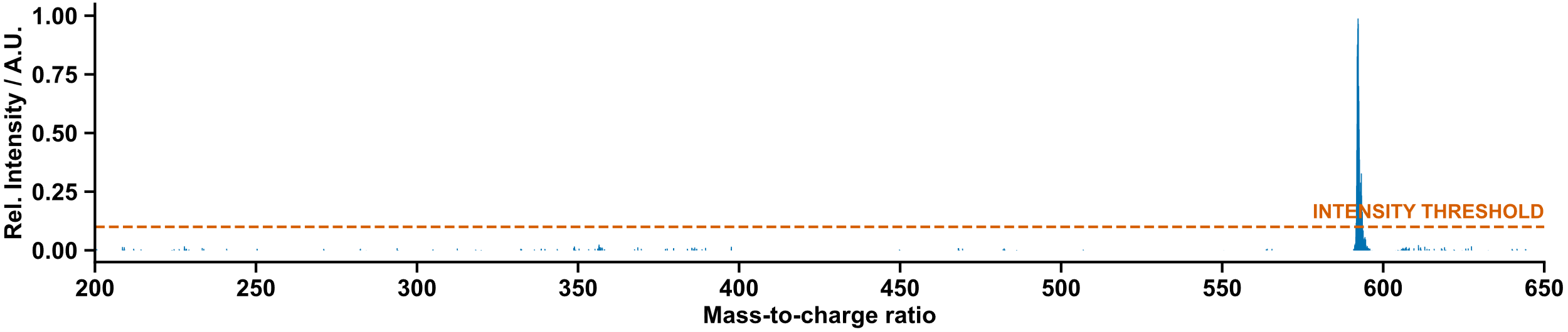


Figure S 80: Mass spectrum of diversification 20.

#### Manual Characterization

Purified by flash column chromatography (CH_2_Cl_2_/Methanol gradient) to yield the product as a white solid.

**^1^H NMR** (400 MHz, DMSO-*d*_6_) δ 11.35 (s, 1H, H^24^), 9.35 (s, 1H, H^7^), 8.12 (s, 1H, H^13^), 8.08 (s, 2H, H^5^), 7.81 (d, *J* = 1.4 Hz, 1H, H^20^), 7.53 (s, 1H, H^4^), 6.60 (t, *J* = 5.7 Hz, 1H, H^9^), 6.42 (t, *J* = 6.6 Hz, 1H, H^15^), 5.33 (dt, *J* = 8.5, 5.3 Hz, 1H, H^14^), 5.28 (t, *J* = 5.2 Hz, 1H, H^18^), 4.19 (dt, *J* = 5.3, 3.5 Hz, 1H, H^16^), 3.74 – 3.65 (m, 1H, H^17^), 3.64 – 3.56 (m, 1H, H^17^), 3.42 (dt, *J* = 7.0, 6.1 Hz, 2H, H^10^), 2.84 (t, *J* = 7.0 Hz, 2H, H^11^), 2.77 – 2.67 (m, 1H, H^19^), 2.67 – 2.57 (m, 1H, H^19^), 1.81 (s, 3H, H^22^).

**^13^C NMR** (101 MHz, DMSO-*d*_6_) δ 163.75 (C^23^), 154.79 (C^8^), 150.47 (C^25^), 144.75 (C^12^), 142.61 (C^6^), 136.22 (C^20^), 130.61 (q, *J* = 32.5 Hz, C^3^), 123.39 (q, *J* = 272.7 Hz, C^2^), 122.10 (C^13^), 117.37 – 117.00 (m, C^5^), 113.69 – 113.27 (m, C^4^), 109.64 (C^21^), 84.54 (C^16^), 83.92 (C^15^), 60.74 (C^17^), 59.07 (C^14^), 38.96 (C^10^), 37.13 (C^19^), 25.94 (C^11^), 12.27 (C^6^).

**^19^F NMR** (376 MHz, DMSO-*d*_6_) δ -61.75 (F^1^).

**HRMS** calculated for C_23_H_24_F_6_N_7_O_5_+: 592.1738; found: 592.1749.

***N.B.*:** C^10^ signal overlaps with DMSO residual solvent signal – assigned by HSQC.


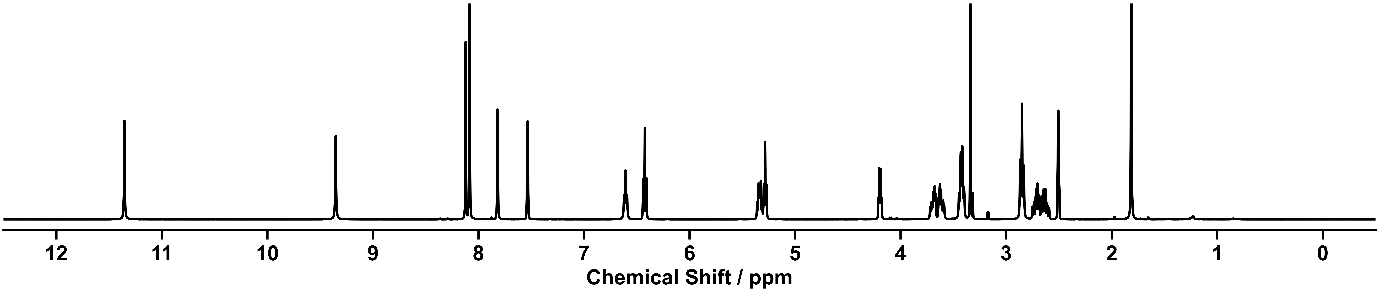


**Figure S 81:** ^1^H NMR spectrum (400 MHz, DMSO-*d*_6_) of diversification **20**.

**Figure S 82**: ^13^C NMR spectrum (101 MHz, DMSO-*d*_6_) of diversification **20**.

### Diversification (21)

#### Outcome of Automation

**Scheme S 20:** Synthesis of **21** from urea **7** and Zidovudine **18**. Reaction conditions: CuSO_4_ (10 mol%), ascorbic acid (20 mol%)_,_ CH_2_Cl_2_:H_2_O:IPA (2:1:1), 60 °C, 14 h.

| *NMR*: N/A | *MS*: Pass |
| --- | --- |
| *Recorded solely for reference* | *retention time*: 1.99 min  *LC area*: 21% |
|  | *ion observed*: [**21**+H]^+^  *m/z expected*: 640.18  *m/z measured*: 640.15 |

**Table S 19:** Summary of automated decision-maker outcome for ULPC-MS spectrometry.

**Figure S 83:** ^1^H NMR spectrum (80 MHz) of screening **21**.

**Figure S 84:** UPLC chromatogram of diversification **21.**

**Figure S 85:** Mass spectrum of diversification **21**.

#### Manual Characterization

Purified by column chromatography (CH_2_Cl_2_/MeOH gradient) to yield the product as a yellow solid.

**^1^H NMR** (400 MHz, CD_3_OD) δ 8.41 (s, 1H, H^13^), 8.12 – 8.06 (m, 2H, H^5^), 7.93 (q, *J* = 1.2 Hz, 1H, H^19^), 7.82 – 7.74 (m, 2H, H^10^), 7.60 – 7.51 (m, 3H, H^9+4^), 6.52 (t, *J* = 6.5 Hz, 1H, H^16^), 5.47 (dt, *J* = 8.6, 5.4 Hz, 1H, H^14^), 4.43 (dt, *J* = 5.8, 3.0 Hz, 1H, H^15^), 3.94 (dd, *J* = 12.3, 3.0 Hz, 1H, H^17^), 3.82 (dd, *J* = 12.3, 3.2 Hz, 1H, H^17^), 3.02 – 2.91 (m, 1H, H^18^), 2.84 – 2.71 (m, 1H, H^18^), 1.92 (d, *J* = 1.2 Hz, 3H, H^21^).

**^13^C NMR** (101 MHz, CD_3_OD) δ 166.41 (C^22^), 154.45 (C^7^), 152.32 (C^23^), 148.77 (C^12^), 142.92 (C^6^), 140.41 (C^11^), 138.27 (C^19^), 133.19 (q, *J* = 33.1 Hz, C^3^), 127.38 (C^10^), 126.20 (C^8^), 124.79 (q, *J* = 272.3 Hz, C^2^), 121.49 (C^13^), 120.74 (C^9^), 119.53 – 119.28 (m, C^5^), 116.17 – 115.89 (m, C^4^), 111.72 (C^20^), 86.74 (C^16^), 86.39 (C^15^), 62.19 (C^17^), 61.20 (C^14^), 39.03 (C^18^), 12.49 (C^21^).

**^19^F NMR** (376 MHz, CD_3_OD) δ -64.60 (F^1^).

**HRMS** calculated for C_27_H_24_F_6_N_7_O_5_+: 640.1738; found: 640.1747.

***N.B.***: C^2^ quartet overlaps with C^8/9^ signals.

**Figure S 86:** ^1^H NMR spectrum (400 MHz, CD_3_OD) of diversification **21**.

**Figure S 87**: ^13^C NMR spectrum (101 MHz, CD_3_OD) of diversification **21**.

### Diversification (22)

#### Outcome of Automation

**Scheme S 21:** Synthesis of **22** from thiourea **10** and Zidovudine **18**. Reaction conditions: CuSO_4_ (10 mol%), ascorbic acid (20 mol%)_,_ CH_2_Cl_2_:H_2_O:IPA (2:1:1), 60 °C, 14 h.

| *NMR*: N/A | *MS*: Pass |
| --- | --- |
| *Recorded solely for reference* | *retention time*: 1.37 min  *LC area*: 21% |
|  | *ion observed*: [**22**+H]^+^  *m/z expected*: 490.17  *m/z measured*: 490.15 |

**Table S 20:** Summary of automated decision-maker outcome for ULPC-MS spectrometry.

**Figure S 88:** ^1^H NMR spectrum (80 MHz) of screening **22**.

**Figure S 89:** UPLC chromatogram of diversification **22**.

**Figure S 90:** Mass spectrum of diversification **22.**

#### Manual Characterization

Purified by column chromatography (CH_2_Cl_2_/MeOH gradient) to yield the product as a white solid.

**^1^H NMR** (400 MHz, CD_3_OD) δ 7.96 (s, 1H, H^10^), 7.91 (q, *J* = 1.2 Hz, 1H, H^16^), 7.32 – 7.23 (m, 2H, H^4^), 7.14 – 7.04 (m, 2H, H^3^), 6.48 (t, *J* = 6.5 Hz, 1H, H^13^), 5.40 (dt, *J* = 8.6, 5.4 Hz, 1H, H^11^), 4.36 (dt, *J* = 5.4, 3.1 Hz, 1H, H^12^), 3.98 – 3.84 (m, 3H, H^7+14^), 3.77 (dd, *J* = 12.3, 3.2 Hz, 1H, H^14^), 3.03 (t, *J* = 6.9 Hz, 2H, H^8^), 2.95 – 2.84 (m, 1H, H^15^), 2.78 – 2.66 (m, 1H, H^15^), 1.91 (d, *J* = 1.2 Hz, 3H, H^18^).

**^13^C NMR** (101 MHz, CD_3_OD) δ 182.81 (C^6^), 166.41 (C^19^), 162.09 (d, *J* = 244.1 Hz, C^2^), 152.31 (C^20^), 146.61 (C^9^), 138.25 (C^16^), 135.57 (C^5^), 128.35 (d, *J* = 7.5 Hz, C^4^), 123.59 (C^10^), 116.82 (d, *J* = 23.0 Hz, C^3^), 111.69 (C^17^), 86.69 (C^13^), 86.39 (C^12^), 62.15 (C^14^), 60.95 (C^11^), 45.01 (C^7^), 39.04 (C^15^), 26.19 (C^8^), 12.48 (C^18^).

**^19^F NMR** (376 MHz, CD_3_OD) δ -118.26 (F^1^).

**HRMS** calculated for C_21_H_25_FN_7_O_4_S+: 490.1667; found: 490.1674.

**Figure S 91:** ^1^H NMR spectrum (400 MHz, CD_3_OD) of diversification **22**.

**Figure S 92**: ^13^C NMR spectrum (101 MHz, CD_3_OD) of diversification **22**.

### Diversification (23)

**Scheme S 22:** Attempted synthesis of **23** from thiourea **8** and Zidovudine **18**. Reaction conditions: CuSO_4_ (10 mol%), ascorbic acid (20 mol%)_,_ CH_2_Cl_2_:H_2_O:IPA (2:1:1), 60 °C, 14 h.

| *NMR*: N/A | *MS*: Fail |
| --- | --- |
| *Recorded solely for reference* | *retention time*: 1.09 min  *LC area*: 90% |
|  | *No matching ions observed* |

**Table S 21:** Summary of automated decision-maker outcome for ULPC-MS spectrometry.

**Figure S 93:** ^1^H NMR spectrum (80 MHz) of screening **23**.

**Figure S 94:** UPLC chromatogram of diversification **23**.

# Autonomous Discovery of Supramolecular Host-Guest Assemblies

## Screening for Discovery of Supramolecular Complexes

**Scheme S 23:** General overview of automated screening for discovery of supramolecular architectures.

20 mL ISynth vials containing pre-weighed quantities of reagents **24 – 29** along with zinc di[bis(trifluoromethylsulfonyl)imide] and tetrakis(acetonitrile)copper(I) tetrafluoroborate were placed inside the Chemspeed, and MeCN was dispensed to prepare stock solutions. Reaction solutions were prepared by dispensing aliquots (1.5 mL) of stock solutions of the relevant amine, aldehyde/ketone, and metal. A further portion of MeCN (3 mL) was added to each of the 18 vials. The vials were heated at 60 °C for 16 hours, and then cooled to ambient temperature for 1.5 hours. Aliquots (0.1 mL) of reactions were reformatted into LC-MS vials and the LC-MS were then diluted with MeCN (1.9 mL). Aliquots (0.7 mL) of the reaction mixture were then reformatted into NMR tubes. UPLC-Agent then transferred the LC-MS vials for analysis by MS, while NMR-Agent transferred the NMR tubes to the benchtop NMR for analysis.

Stock Solution Concentrations:

**24, 25, 26** – 40 mM

**29,** Tetrakis(acetonitrile)copper(I) tetrafluoroborate – 20 mM

**27**, **28**, zinc di[bis(trifluoromethylsulfonyl)imide] – 13.3 mM

## Screening Results

### Supramolecular Screening One

#### Outcome of Automation

**Scheme S 24**: Self-assembly of components **24** and **28** with Zn(NTf_2_)_2_ in a 3:1:1 molar ratio in CH_3_CN at 60 °C for 16 h.

| *NMR*: Pass | *MS*: Pass |
| --- | --- |
| *Single set of peaks different from the starting materials*  *Total number of peaks within range* | *ion observed*: [Zn_4_(**24**_3_,**28**)_4_](NTf_2_)_2_  *charge*: 6+  *m/z expected*: 549.26  *m/z measured*: 549.59  *ion observed*: [Zn_4_(**24**_3_,**28**)_4_](NTf_2_)_3_  *charge*: 5+  *m/z expected*: 715.10  *m/z measured*: 715.21 |

**Table S 22:** Summary of automated decision-maker outcomes for ^1^H NMR spectroscopy and ULPC-MS spectrometry.

**Figure S 95:** ^1^H NMR spectra (80 MHz, MeCN) of aldehyde **24** (top), amine **28** (middle), screening one (bottom).

**Figure S 96:** Mass spectrum of screening one.

#### Manual Experiment and Chemist Comment

In agreement with the autonomous decision-maker, the complex [Zn_4_(**24**_3_, **29**)_4_](NTf_2_)_8_ was formed as reported previously in the literature.^6^

**Figure S 97:** ^1^H NMR spectra (400 MHz, CD_3_CN) of the self-assembly of components **24** and **28** with Zn(NTf_2_)_2_ in a 3:1:1 molar ratio in CH_3_CN at 60 °C for 16 h executed manually.

### Supramolecular Screening Two

#### Outcome of Automation

**Scheme S 25**: Self-assembly of components **25** and **28** with Zn(NTf_2_)_2_ in a 3:1:1 molar ratio in CH_3_CN at 60 °C for 16 h.

| *NMR*: Fail | *MS*: Fail |
| --- | --- |
| *Not enough chemical shift* | *No matching ions observed* |

**Table S 23:** Summary of automated decision-maker outcomes for ^1^H NMR spectroscopy and ULPC-MS spectrometry.

**Figure S 98:** ^1^H NMR spectra (80 MHz, MeCN) of ketone **25** (top), amine **28** (middle), screening two (bottom).

**Figure S 99:** Mass spectrum of screening two**.**

#### Manual Experiment and Chemist Comment

In agreement with the decision-maker, no signs of a clear supramolecular architecture were observed.

**Figure S 100:** ^1^H NMR spectra (400 MHz, CD_3_CN) of the self-assembly of components **25** and **28** with Zn(NTf_2_)_2_ in a 3:1:1 molar ratio in CH_3_CN at 60 °C for 16 h executed manually.

### Supramolecular Screening Three

#### Outcome of Automation

**Scheme S 26**: Self-assembly of components **26** and **28** with Zn(NTf_2_)_2_ in a 3:1:1 molar ratio in CH_3_CN at 60 °C for 16 h.

| *NMR*: Fail | *MS*: Fail |
| --- | --- |
| *Too many peaks* | *No matching ions observed* |

**Table S 24:** Summary of automated decision-maker outcomes for ^1^H NMR spectroscopy and ULPC-MS spectrometry.

**Figure S 101:** ^1^H NMR spectra (80 MHz, MeCN) of aldehyde **26** (top), amine **28** (middle), screening three (bottom).

**Figure S 102:** Mass spectrum of screening three.

#### Manual Experiment and Chemist Comment

In agreement with the decision-maker, no signs of a clear supramolecular architecture were observed.

**Figure S 103:** ^1^H NMR spectra (400 MHz, CD_3_CN) of the self-assembly of components **26** and **28** with Zn(NTf_2_)_2_ in a 3:1:1 molar ratio in CH_3_CN at 60 °C for 16 h executed manually.

### Supramolecular Screening Four

#### Outcome of Automation

**Scheme S 27**: Self-assembly of components **24** and **27** with Zn(NTf_2_)_2_ in a 3:1:1 molar ratio in CH_3_CN at 60 °C for 16 h.

| *NMR*: Fail | *MS*: Fail |
| --- | --- |
| *Too many peaks* | *No matching ions observed* |

**Table S 25:** Summary of automated decision-maker outcomes for ^1^H NMR spectroscopy and ULPC-MS spectrometry.

**Figure S 104:** ^1^H NMR spectra (80 MHz, MeCN) of aldehyde **24** (top), amine **27** (middle), screening four (bottom)

**Figure S 105:** Mass spectrum of screening four.

#### Manual Experiment and Chemist Comment

The complex [Zn(**24**_3_, **27**)](NTf_2_)_2_ was formed as previously reported in the literature.^7^ During the automated experimentation, this complex was not selected for scale-up due to the imprecise ^1^H NMR peak picking at 80 MHz, resulting in too many peaks being selected for the complex when compared to the starting materials.

**Figure S 106:** ^1^H NMR spectra (400 MHz, CD_3_CN) of the self-assembly of components **24** and **27** with Zn(NTf_2_)_2_ in a 3:1:1 molar ratio in CH_3_CN at 60 °C for 16 h executed manually.

### Supramolecular Screening Five

#### Outcome of Automation

**Scheme S 28**: Self-assembly of components **25** and **27** with Zn(NTf_2_)_2_ in a 3:1:1 molar ratio in CH_3_CN at 60 °C for 16 h.

| *NMR*: Fail | *MS*: Fail |
| --- | --- |
| *Not enough chemical shift* | *No matching ions observed* |

**Table S** 26**:** Summary of automated decision-maker outcomes for ^1^H NMR spectroscopy and ULPC-MS spectrometry.

**Figure S 107:** ^1^H NMR spectra (80 MHz, MeCN) of ketone **25** (top), amine **27** (middle), screening five (bottom).

**Figure S 108:** Mass spectrum of screening five.

#### Manual Experiment and Chemist Comment

In agreement with the decision-maker, no clear signs of a single, dominant supramolecular architecture were observed.

**Figure S 109:** ^1^H NMR spectra (400 MHz, CD_3_CN) of the self-assembly of components **25** and **27** with Zn(NTf_2_)_2_ in a 3:1:1 molar ratio in CH_3_CN at 60 °C for 16 h executed manually.

### Supramolecular Screening Six

#### Outcome of Automation

**Scheme S 29**: Self-assembly of components **26** and **27** with Zn(NTf_2_)_2_ in a 3:1:1 molar ratio in CH_3_CN at 60 °C for 16 h.

| *NMR*: Fail | *MS*: Fail |
| --- | --- |
| *Too many peaks* | *No matching ions observed* |

**Table S 27:** Summary of automated decision-maker outcomes for ^1^H NMR spectroscopy and ULPC-MS spectrometry.

**Figure S 110:** ^1^H NMR spectra (80 MHz, MeCN) of aldehyde **26** (top), amine **27** (middle), screening six (bottom).

**Figure S 111:** Mass spectrum of screening six.

#### Manual Experiment and Chemist Comment

The spectra contains a mixture of the complex [Zn(**26**_3_, **27**)](NTf_2_)_2_ (previously reported in the literature)^8,9^ and unreacted starting material. The presence of a large amount of starting material besides the complex (*i.e.* the low reaction yield) prevented its selection by the autonomous decision maker.

**Figure S 112:** ^1^H NMR spectra (400 MHz, CD_3_CN) of the self-assembly of components **24** and **27** with Zn(NTf_2_)_2_ in a 3:1:1 molar ratio in CH_3_CN at 60 °C for 16 h executed manually.

### Supramolecular Screening Seven

#### Outcome of Automation

**Scheme S 30**: Self-assembly of components **24** and **29** with Zn(NTf_2_)_2_ in a 6:3:2 molar ratio in CH_3_CN at 60 °C for 16 h.

| *NMR*: Pass | *MS*: Pass |
| --- | --- |
| *Single set of peaks different from the starting materials*  *Total number of peaks within range* | *ion observed*: [Zn_2_(**24**_2_,**29**)_3_]^4+^  *charge*: 4+  *m/z expected*: 314.59  *m/z measured*: 314.99*  *ion observed*: [Zn_2_(**24**_2_,**29**)_3_](NTf_2_)  *charge*: 3+  *m/z expected*: 512.76  *m/z measured*: 512.96 |

** Due to the small difference between the mass-to-charge ration expected for [Zn_2_(****24****_2_,****29****)_3_]^4+^ (314.59) and* *[Zn_4_(****24****_2_,****29****)_6_]^8+^ (314.97) the peak in the original summary JSON is attributed to the closer match [Zn_4_(****24****_2_,****29****)_6_]^8+^.*

**Table S 28:** Summary of automated decision-maker outcomes for ^1^H NMR spectroscopy and ULPC-MS spectrometry.

**Figure S 113:** ^1^H NMR spectra (80 MHz, MeCN) of aldehyde **24** (top), amine **29** (middle), screening seven (bottom).

Figure S 114: Mass spectrum of screening seven.

#### Manual Experiment and Chemist Comment

In agreement with the autonomous decision-maker, the complex [Zn_2_(**24**_2_, **29**)_3_](NTf_2_)_4_ was formed as previously reported in the literature.^10^

**Figure S 115:** ^1^H NMR spectra (400 MHz, CD_3_CN) of the self-assembly of components **24** and **29** with Zn(NTf_2_)_2_ in a 6:3:2 molar ratio in CH_3_CN at 60 °C for 16 h executed manually.

### Supramolecular Screening Eight

#### Outcome of Automation

**Scheme S 31**: Self-assembly of components **25** and **29** with Zn(NTf_2_)_2_ in a 6:3:2 molar ratio in CH_3_CN at 60 °C for 16 h.

| *NMR*: Fail | *MS*: Pass |
| --- | --- |
| *Too many peaks* | *ion observed*: [Zn_2_(**25**_2_,**29**)_3_]^4+^  *charge*: 4+  *m/z expected*: 335.82  *m/z measured*: 335.61 |

**Table S 29:** Summary of automated decision-maker outcomes for ^1^H NMR spectroscopy and ULPC-MS spectrometry.

**Figure S 116:** ^1^H NMR spectra (80 MHz, MeCN) of ketone **25** (top), amine **29** (middle), screening eight (bottom).

**Figure S 117:** Mass spectrum of screening eight.

#### Manual Experiment and Chemist Comment

In agreement with the decision-maker, no clear signs of a supramolecular architecture were observed.

**Figure S 118:** ^1^H NMR spectra (400 MHz, CD_3_CN) of the self-assembly of components **25** and **29** with Zn(NTf_2_)_2_ in a 6:3:2 molar ratio in CH_3_CN at 60 °C for 16 h executed manually.

### Supramolecular Screening Nine

#### Outcome of Automation

**Scheme S 32**: Self-assembly of components **26** and **29** with Zn(NTf_2_)_2_ in a 6:3:2 molar ratio in CH_3_CN at 60 °C for 16 h.

| *NMR*: Pass | *MS*: Fail |
| --- | --- |
| *Single set of peaks different from the starting materials*  *Total number of peaks within range* | *No matching ions observed* |

**Table S 30:** Summary of automated decision-maker outcomes for ^1^H NMR spectroscopy and ULPC-MS spectrometry.

**Figure S 119:** ^1^H NMR spectra (80 MHz, MeCN) of aldehyde **26** (top), amine **29** (middle), screening nine (bottom).

**Figure S 120:** Mass spectrum of screening nine.

#### Manual Experiment and Chemist Comment

In agreement with the decision-maker, it appears by ^1^H NMR spectroscopy that a defined supra-molecular architecture was potentially formed (limited number of peaks, hinting at the formation of a highly symmetrical complex). However, the nature of the structure form could not be determined because the complex could not be observed in mass spectrometry, and crystals suitable for x-ray diffraction could not be obtained. This system is the subject of ongoing manual study.

**Figure S 121:** ^1^H NMR spectra (400 MHz, CD_3_CN) of the self-assembly of components **26** and **29** with Zn(NTf_2_)_2_ in a 6:3:2 molar ratio in CH_3_CN at 60 °C for 16 h executed manually.

### Supramolecular Screening Ten

#### Outcome of Automation

**Scheme S 33**: Self-assembly of components **24** and **28** with CuBF_4_ in a 6:2:3 molar ratio in CH_3_CN at 60 °C for 16 h.

| *NMR*: Pass | *MS*: Fail |
| --- | --- |
| *Single set of peaks different from the starting materials*  *Total number of peaks within range* | *No matching ions observed* |

**Table S 31:** Summary of automated decision-maker outcomes for ^1^H NMR spectroscopy and ULPC-MS spectrometry.

**Figure S 122:** ^1^H NMR spectra (80 MHz, MeCN) of aldehyde **24** (top), amine **28** (middle), screening ten (bottom).

**Figure S 123:** Mass spectrum of screening ten.

#### Manual Experiment and Chemist Comment

In agreement with the decision-maker, no clear signs of a supramolecular architecture were observed.

**Figure S 124:** ^1^H NMR spectra (400 MHz, CD_3_CN) of the self-assembly components **24** and **28** with CuBF_4_ in a 6:2:3 molar ratio in CH_3_CN at 60 °C for 16 h executed manually.

### Supramolecular Screening Eleven

#### Outcome of Automation

**Scheme S 34**: Self-assembly of components **25** and **28** with CuBF_4_ in a 6:2:3 molar ratio in CH_3_CN at 60 °C for 16 h.

| *NMR*: Fail | *MS*: Fail |
| --- | --- |
| *Not enough chemical shift* | *No matching ions observed* |

**Table S 32:** Summary of automated decision-maker outcomes for ^1^H NMR spectroscopy and ULPC-MS spectrometry.

**Figure S 125:** ^1^H NMR spectra (80 MHz, MeCN) of ketone **25** (top), amine **28** (middle), screening eleven (bottom).

**Figure S 126:** Mass spectrum of screening **11**.

#### Manual Experiment and Chemist Comment

In agreement with the decision-maker, no clear signs of a supramolecular architecture were observed.

**Figure S 127:** ^1^H NMR spectra (400 MHz, CD_3_CN) of the self-assembly components **25** and **28** with CuBF_4_ in a 6:2:3 molar ratio in CH_3_CN at 60 °C for 16 h executed manually.

### Supramolecular Screening Twelve

#### Outcome of Automation

**Scheme S 35**: Self-assembly of components **26** and **28** with CuBF_4_ in a 6:2:3 molar ratio in CH_3_CN at 60 °C for 16 h.

| *NMR*: Pass | *MS*: Fail |
| --- | --- |
| *Single set of peaks different from the starting materials*  *Total number of peaks within range* | *No matching ions observed* |

**Table S 33:** Summary of automated decision-maker outcomes for ^1^H NMR spectroscopy and ULPC-MS spectrometry.

**Figure S 128:** ^1^H NMR spectra (80 MHz, MeCN) of aldehyde **26** (top), amine **28** (middle), screening twelve (bottom).

**Figure S 129:** Mass spectrum of screening twelve.

#### Manual Experiment and Chemist Comment

In agreement with the decision-maker, no clear signs of a supramolecular architecture were observed.

**Figure S 130:** ^1^H NMR spectra (400 MHz, CD_3_CN) of the self-assembly components **26** and **28** with CuBF_4_ in a 6:2:3 molar ratio in CH_3_CN at 60 °C for 16 h executed manually.

### Supramolecular Screening Thirteen

#### Outcome of Automation

**Scheme S 36**: Self-assembly of components **24** and **27** with CuBF_4_ in a 6:2:3 molar ratio in CH_3_CN at 60 °C for 16 h.

| *NMR*: Fail | *MS*: Fail |
| --- | --- |
| *Not enough peaks* | *No matching ions observed* |

**Table S 34:** Summary of automated decision-maker outcomes for ^1^H NMR spectroscopy and ULPC-MS spectrometry.

**Figure S 131:** ^1^H NMR spectra (80 MHz, MeCN) of aldehyde **24** (top), amine **27** (middle), screening thirteen (bottom).

**Figure S 132:** Mass spectrum of screening thirteen**.**

#### Manual Experiment and Chemist Comment

In agreement with the decision-maker, no signs of a clear supramolecular architecture were observed.

**Figure S 133:** ^1^H NMR spectra (400 MHz, CD_3_CN) of the self-assembly components **24** and **27** with CuBF_4_ in a 6:2:3 molar ratio in CH_3_CN at 60 °C for 16 h executed manually.

### Supramolecular Screening Fourteen

#### Outcome of Automation

**Scheme S 37**: Self-assembly of components **25** and **27** with CuBF_4_ in a 6:2:3 molar ratio in CH_3_CN at 60 °C for 16 h.

| *NMR*: Fail | *MS*: Fail |
| --- | --- |
| *Not enough peaks* | *No matching ions observed* |

**Table S 35:** Summary of automated decision-maker outcomes for ^1^H NMR spectroscopy and ULPC-MS spectrometry.

**Figure S 134:** ^1^H NMR spectra (80 MHz, MeCN) of ketone **25** (top), amine **27** (middle), screening fourteen (bottom).

**Figure S 135:** Mass spectrum of screening fourteen.

#### Manual Experiment and Chemist Comment

In agreement with the decision-maker, no clear signs of a supramolecular architecture were observed.

**Figure S 136:** ^1^H NMR spectra (400 MHz, CD_3_CN) of the self-assembly components **25** and **27** with CuBF_4_ in a 6:2:3 molar ratio in CH_3_CN at 60 °C for 16 h executed manually.

### Supramolecular Screening Fifteen

#### Outcome of Automation

**Scheme S 38**: Self-assembly of components **26** and **27** with CuBF_4_ in a 6:2:3 molar ratio in CH_3_CN at 60 °C for 16 h.

| *NMR*: Pass | *MS*: Fail |
| --- | --- |
| *Single set of peaks different from the starting materials*  *Total number of peaks within range* | *No matching ions observed* |

**Table S 36:** Summary of automated decision-maker outcomes for ^1^H NMR spectroscopy and ULPC-MS spectrometry.

**Figure S 137:** ^1^H NMR spectra (80 MHz, MeCN) of aldehyde **26** (top), amine **27** (middle), screening fifteen (bottom).

**Figure S 138:** Mass spectrum of screening fifteen.

#### Manual Experiment and Chemist Comment

In agreement with the decision-maker, no clear signs of a supramolecular architecture were observed.

**Figure S 139:** ^1^H NMR spectra (400 MHz, CD_3_CN) of the self-assembly components **26** and **27** with CuBF_4_ in a 6:2:3 molar ratio in CH_3_CN at 60 °C for 16 h executed manually.

### Supramolecular Screening Sixteen

#### Outcome of Automation

**Scheme S 39**: Self-assembly of components **24** and **29** with CuBF_4_ in a 2:1:1 molar ratio in CH_3_CN at 60 °C for 16 h.

| *NMR*: Pass | *MS*: Fail |
| --- | --- |
| *Single set of peaks different from the starting materials*  *Total number of peaks within range* | *No matching ions observed* |

**Table S 37:** Summary of automated decision-maker outcomes for ^1^H NMR spectroscopy and ULPC-MS spectrometry.

**Figure S 140:** ^1^H NMR spectra (80 MHz, MeCN) of aldehyde **24** (top), amine **29** (middle), screening sixteen (bottom).

**Figure S 141:** Mass spectrum of screening sixteen.

#### Manual Experiment and Chemist Comment

The complex [Cu_2_(**24**_2_, **29**)_2_](BF_4_)_2_ was formed as previously reported in the literature, in keeping with the ^1^H NMR data.^11^ However, during the automated experimentation, this complex was *not* selected because it decomposed in the mass spectrometer under the analysis conditions used.

**Figure S 142:** ^1^H NMR spectra (400 MHz, CD_3_CN) of the self-assembly components **24** and **29** with CuBF_4_ in a 2:1:1 molar ratio in CH_3_CN at 60 °C for 16 h executed manually.

### Supramolecular Screening Seventeen

#### Outcome of Automation

**Scheme S 40**: Self-assembly of components **25** and **29** with CuBF_4_ in a 2:1:1 molar ratio in CH_3_CN at 60 °C for 16 h.

| *NMR*: Fail | *MS*: Fail |
| --- | --- |
| *Not enough chemical shift* | *No matching ions observed* |

**Table S 38:** Summary of automated decision-maker outcomes for ^1^H NMR spectroscopy and ULPC-MS spectrometry.

**Figure S 143:** ^1^H NMR spectra (80 MHz, MeCN) of ketone **25** (top), amine **29** (middle), screening seventeen (bottom).

**Figure S 144:** Mass spectrum of screening seventeen.

#### Manual Experiment and Chemist Comment

In agreement with the decision-maker, no clear signs of a supramolecular architecture were observed.

**Figure S 145:** ^1^H NMR spectra (400 MHz, CD_3_CN) of the self-assembly components **25** and **29** with CuBF_4_ in a 2:1:1 molar ratio in CH_3_CN at 60 °C for 16 h executed manually.

### Supramolecular Screening Eighteen

#### Outcome of Automation

**Scheme S 41**: Self-assembly of components **26** and 2**9** with CuBF_4_ in a 2:1:1 molar ratio in CH_3_CN at 60 °C for 16 h.

| *NMR*: Fail | *MS*: Pass |
| --- | --- |
| *Too many peaks* | *ion observed*: [Cu_2_(**26**_2_,**29**)_2_]^2+^  *charge*: 2+  *m/z expected*: 467.13  *m/z measured*: 467.31 |

**Table S 39:** Summary of automated decision-maker outcomes for ^1^H NMR spectroscopy and ULPC-MS spectrometry.

**Figure S 146:** ^1^H NMR spectra (80 MHz, MeCN) of aldehyde **26** (top), amine **29** (middle), screening eighteen (bottom).

**Figure S 147:** Mass spectrum of screening eighteen.

#### Manual Experiment and Chemist Comment

The complex [Cu_2_(**26**_2_,**29**)_2_](BF_4_)_2_ was formed (see below) alongside leftover starting material, in keeping with the MS “pass”. The presence of a large amount of starting material besides the complex (*i.e.,* the low reaction yield) prevented its selection by the autonomous decision maker.

**Figure S 148:** ^1^H NMR spectra (400 MHz, CD_3_CN) of the self-assembly components **26** and **29** with CuBF_4_ in a 2:1:1 molar ratio in CH_3_CN at 60 °C for 16 h executed manually.

**^1^H NMR** (400 MHz, CD_3_CN) δ 9.19 (s, 2H, H^7^), 8.07 (t, *J* = 7.7 Hz, 2H, H^4^), 7.90 (d, *J* = 8.0 Hz, 1H, H^5^), 7.62 (d, *J* = 7.8 Hz, 2H, H^3^), 7.36 (d, *J* = 7.9 Hz, 4H, H^9^), 7.17 (d, *J* = 8.1 Hz, 4H, H^10^), 3.85 (s, 2H, H^12^), 2.24 (s, 6H, H^1^).

**^13^C NMR** (101 MHz, CD_3_CN) δ 159.01 (C^2^), 158.01 (C^7^), 151.18 (C^6^), 144.87 (C^11^), 144.08 (C^8^), 139.21 (C^4^), 130.18 (C^10^), 129.04 (C^3^), 126.41 (C^5^), 123.65 (C^9^), 41.12 (C^12^), 24.70 (C^1^).

**HRMS** calculated for [Cu_2_(**26**_2_,**29**)_2_]^2+^ 467.1292; found: 467.1296.

## Replication Experiments

Replication experiments were performed analogously to the screening experiments except larger volumes of the stock solutions were prepared inside the Chemspeed. The supramolecular complexes chosen for replication (cage [Zn_4_(**24**_3_,**28**)_4_]^8+^ and helicate [Zn_2_(**24**_2_,**29**)_3_]^4+^ in this case) were synthesized in parallel 6 times each. Samples for NMR and MS were prepared and transported identically to the screening stage.

## Replication Results

### Replication of Cage [Zn_4_(24_3_,28)_4_]^8+^

**Scheme S 42:** Self-assembly of components **24** and **28** with Zn(NTf_2_)_2_ in a 3:1:1 molar ratio in CH_3_CN at 60 °C for 16 h.

**Figure S 149:** ^1^H NMR spectra (80 MHz, MeCN) of replications of cage [Zn_4_(**24**_3_,**28**)_4_]^8+^_._

#### Replication One

| *NMR*: Pass | *MS*: Pass |
| --- | --- |
| *Matches reference from screening experiment* | *ion observed*: [Zn_4_(**24**_3_,**28**)_4_](NTf_2_)_2_  *charge*: 6+  *m/z expected*: 549.26  *m/z measured*: 549.09  *ion observed*: [Zn_4_(**24**_3_,**28**)_4_](NTf_2_)  *charge*: 7+  *m/z expected*: 430.81  *m/z measured*: 431.01 |

**Table S 40:** Summary of automated decision-maker outcomes for ^1^H NMR spectroscopy and ULPC-MS spectrometry.

**Figure S 150**: Mass spectrum of cage [Zn_4_(**24**_3_,**28**)_4_]^8+^ replicate one**.**

**Figure S 151:** Dynamic time warp comparison of cage [Zn_4_(**24**_3_,**28**)_4_]^8+^ replicate one (top) and screening (below).

#### Replication Two

| *NMR*: Pass | *MS*: Pass |
| --- | --- |
| *Matches reference from screening experiment* | *ion observed*: [Zn_4_(**24**_3_,**28**)_4_](NTf_2_)_2_  *charge*: 6+  *m/z expected*: 549.26  *m/z measured*: 549.34  *ion observed*: [Zn_4_(**24**_3_,**28**)_4_](NTf_2_)  *charge*: 7+  *m/z expected*: 430.81  *m/z measured*: 430.92 |

**Table S 41:** Summary of automated decision-maker outcomes for ^1^H NMR spectroscopy and ULPC-MS spectrometry.

**Figure S 152**: Mass spectrum of cage [Zn_4_(**24**_3_,**28**)_4_]^8+^ replicate two.

**Figure S 153:** Dynamic time warp comparison of cage [Zn_4_(**24**_3_,**28**)_4_]^8+^ replicate two (top) and screening (below).

#### Replication Three

| *NMR*: Pass | *MS*: Pass |
| --- | --- |
| *Matches reference from screening experiment* | *ion observed*: [Zn_4_(**24**_3_,**28**)_4_](NTf_2_)_2_  *charge*: 6+  *m/z expected*: 549.26  *m/z measured*: 548.92  *ion observed*: [Zn_4_(**24**_3_,**28**)_4_](NTf_2_)  *charge*: 7+  *m/z expected*: 430.81  *m/z measured*: 431.01 |

**Table S 42:** Summary of automated decision-maker outcomes for ^1^H NMR spectroscopy and ULPC-MS spectrometry.

**Figure S 154**: Mass spectrum of cage [Zn_4_(**24**_3_,**28**)_4_]^8+^ replicate three.

**Figure S 155:** Dynamic time warp comparison of cage [Zn_4_(**24**_3_,**28**)_4_]^8+^ replicate three (top) and screening (below).

#### Replication Four

| *NMR*: Pass | *MS*: Pass |
| --- | --- |
| *Matches reference from screening experiment* | *ion observed*: [Zn_4_(**24**_3_,**28**)_4_](NTf_2_)_2_  *charge*: 6+  *m/z expected*: 549.26  *m/z measured*: 549.60  *ion observed*: [Zn_4_(**24**_3_,**28**)_4_](NTf_2_)_2_  *charge*: 5+  *m/z expected*: 715.10  *m/z measured*: 715.12 |

**Table S 43:** Summary of automated decision-maker outcomes for ^1^H NMR spectroscopy and ULPC-MS spectrometry.

**Figure S 156**: Mass spectrum of cage [Zn_4_(**24**_3_,**28**)_4_]^8+^ replicate four.

**Figure S 157:** Dynamic time warp comparison of cage [Zn_4_(**24**_3_,**28**)_4_]^8+^ replicate four (top) and screening (below).

#### Replication Five

| *NMR*: Pass | *MS*: Pass |
| --- | --- |
| *Matches reference from screening experiment* | *ion observed*: [Zn_4_(**24**_3_,**28**)_4_](NTf_2_)_2_  *charge*: 6+  *m/z expected*: 549.26  *m/z measured*: 549.43  *ion observed*: [Zn_4_(**24**_3_,**28**)_4_](NTf_2_)  *charge*: 7+  *m/z expected*: 430.81  *m/z measured*: 430.76 |

**Table S 44:** Summary of automated decision-maker outcomes for ^1^H NMR spectroscopy and ULPC-MS spectrometry.

**Figure S 158**: Mass spectrum of cage [Zn_4_(**24**_3_,**28**)_4_]^8+^ replicate five.

**Figure S 159:** Dynamic time warp comparison of cage [Zn_4_(**24**_3_,**28**)_4_]^8+^ replicate five (top) and screening (below).

#### Replication Six

| *NMR*: Pass | *MS*: Pass |
| --- | --- |
| *Matches reference from screening experiment* | *ion observed*: [Zn_4_(**24**_3_,**28**)_4_](NTf_2_)_2_  *charge*: 6+  *m/z expected*: 549.26  *m/z measured*: 549.17  *ion observed*: [Zn_4_(**24**_3_,**28**)_4_](NTf_2_)_2_  *charge*: 5+  *m/z expected*: 715.10  *m/z measured*: 715.29 |

**Table S 45:** Summary of automated decision-maker outcomes for ^1^H NMR spectroscopy and ULPC-MS spectrometry.

**Figure S 160**: Mass spectrum of cage [Zn_4_(**24**_3_,**28**)_4_]^8+^ replicate six.

**Figure S 161:** Dynamic time warp comparison of cage [Zn_4_(**24**_3_,**28**)_4_]^8+^ replicate six (top) and screening (below).

### Replication of Helicate [Zn_2_(24_2_,29)_3_]^4+^

**Scheme S 43:** Self-assembly of components **24** and **29** with Zn(NTf_2_)_2_ in a 6:3:2 molar ratio in CH_3_CN at 60 °C for 16 h.

**Figure S 162:** ^1^H NMR spectra (80 MHz, MeCN) of replications of helicate [Zn_2_(**24**_2_,**29**)_3_]^4+^.

#### Replication One

| *NMR*: Pass | *MS*: Pass |
| --- | --- |
| *Matches reference from screening experiment* | *ion observed*: [Zn_2_(**24**_2_,**29**)_3_]^4+^  *charge*: 4+  *m/z expected*: 314.59  *m/z measured*: 315.07  *ion observed*: [Zn_2_(**24**_2_,**29**)_3_](NTf_2_)  *charge*: 3+  *m/z expected*: 512.76  *m/z measured*: 512.44 |

**Table S 46:** Summary of automated decision-maker outcomes for ^1^H NMR spectroscopy and ULPC-MS spectrometry.

**Figure S 163**: Mass spectrum of helicate[Zn_2_(**24**_2_,**29**)_3_]^4+^ replicate one.

**Figure S 164:** Dynamic time warp comparison of helicate [Zn_2_(**24**_2_,**29**)_3_]^4+^ replicate one (top) and screening (below).

#### Replication Two

| *NMR*: Pass | *MS*: Pass |
| --- | --- |
| *Matches reference from screening experiment* | *ion observed*: [Zn_2_(**24**_2_,**29**)_3_]^4+^  *charge*: 4+  *m/z expected*: 314.59  *m/z measured*: 314.91  *ion observed*: [Zn_2_(**24**_2_,**29**)_3_](NTf_2_)  *charge*: 3+  *m/z expected*: 512.76  *m/z measured*: 512.61 |

**Table S 47:** Summary of automated decision-maker outcomes for ^1^H NMR spectroscopy and ULPC-MS spectrometry.

**Figure S 165**: Mass spectrum of helicate [Zn_2_(**24**_2_,**29**)_3_]^4+^ replicate two.

**Figure S 166:** Dynamic time warp comparison of helicate [Zn_2_(**24**_2_,**29**)_3_]^4+^ replicate two (top) and screening (below).

#### Replication Three

| *NMR*: Pass | *MS*: Pass |
| --- | --- |
| *Matches reference from screening experiment* | *ion observed*: [Zn_2_(**24**_2_,**29**)_3_]^4+^  *charge*: 4+  *m/z expected*: 314.59  *m/z measured*: 314.99* |

** Due to the small difference between the mass-to-charge ration expected for [Zn_2_(****24****_2_,****29****)_3_]^4+^ (314.59) and* *[Zn_4_(****24****_2_,****29****)_6_]^8+^ (314.97) the peak in the original summary JSON is attributed to the closer match [Zn_4_(****24****_2_,****29****)_6_]^8+^.*

**Table S 48:** Summary of automated decision-maker outcomes for ^1^H NMR spectroscopy and ULPC-MS spectrometry.

**Figure S 167**: Mass spectrum of helicate [Zn_2_(**24**_2_,**29**)_3_]^4+^ replicate three.

**Figure S 168:** Dynamic time warp comparison of helicate [Zn_2_(**24**_2_,**29**)_3_]^4+^ replicate three (top) and screening (below).

#### Replication Four

| *NMR*: Pass | *MS*: Pass |
| --- | --- |
| *Matches reference from screening experiment* | *ion observed*: [Zn_2_(**24**_2_,**29**)_3_]^4+^  *charge*: 4+  *m/z expected*: 314.59  *m/z measured*: 314.99*  *ion observed*: [Zn_2_(**24**_2_,**29**)_3_](NTf_2_)  *charge*: 3+  *m/z expected*: 512.76  *m/z measured*: 512.36 |

** Due to the small difference between the mass-to-charge ration expected for [Zn_2_(****24****_2_,****29****)_3_]^4+^ (314.59) and* *[Zn_4_(****24****_2_,****29****)_6_]^8+^ (314.97) the peak in the original summary JSON is attributed to the closer match [Zn_4_(****24****_2_,****29****)_6_]^8+^.*

**Table S 49:** Summary of automated decision-maker outcomes for ^1^H NMR spectroscopy and ULPC-MS spectrometry.

**Figure S 169**: Mass spectrum of helicate [Zn_2_(**24**_2_,**29**)_3_]^4+^ replicate four.

**Figure S 170:** Dynamic time warp comparison of helicate [Zn_2_(**24**_2_,**29**)_3_]^4+^ replicate four (top) and screening (below).

#### Replication Five

| *NMR*: Pass | *MS*: Pass |
| --- | --- |
| *Matches reference from screening experiment* | *ion observed*: [Zn_2_(**24**_2_,**29**)_3_]^4+^  *charge*: 4+  *m/z expected*: 314.59  *m/z measured*: 314.99*  *ion observed*: [Zn_2_(**24**_2_,**29**)_3_](NTf_2_)  *charge*: 3+  *m/z expected*: 512.76  *m/z measured*: 512.36 |

** Due to the small difference between the mass-to-charge ration expected for [Zn_2_(****24****_2_,****29****)_3_]^4+^ (314.59) and* *[Zn_4_(****24****_2_,****29****)_6_]^8+^ (314.97) the peak in the original summary JSON is attributed to the closer match [Zn_4_(****24****_2_,****29****)_6_]^8+^.*

**Table S 50:** Summary of automated decision-maker outcomes for ^1^H NMR spectroscopy and ULPC-MS spectrometry.

**Figure S 171**: Mass spectrum of helicate [Zn_2_(**24**_2_,**29**)_3_]^4+^ replicate five.

**Figure S 172:** Dynamic time warp comparison of helicate [Zn_2_(**24**_2_,**29**)_3_]^4+^ replicate five (top) and screening (below).

#### Replication Six

| *NMR*: Pass | *MS*: Pass |
| --- | --- |
| *Matches reference from screening experiment* | *ion observed*: [Zn_2_(**24**_2_,**29**)_3_]^4+^  *charge*: 4+  *m/z expected*: 314.59  *m/z measured*: 314.99* |

** Due to the small difference between the mass-to-charge ration expected for [Zn_2_(****24****_2_,****29****)_3_]^4+^ (314.59) and* *[Zn_4_(****24****_2_,****29****)_6_]^8+^ (314.97) the peak in the original summary JSON is attributed to the closer match [Zn_4_(****24****_2_,****29****)_6_]^8+^.*

**Table S 51:** Summary of automated decision-maker outcomes for ^1^H NMR spectroscopy and ULPC-MS spectrometry.

**Figure S 173**: Mass spectrum of helicate [Zn_2_(**24**_2_,**29**)_3_]^4+^ replicate six.

**Figure S 174:** Dynamic time warp comparison of helicate [Zn_2_(**24**_2_,**29**)_3_]^4+^ replicate six (top) and screening (below).

## Host-Guest Binding Experiments

**Scheme S 44**: Host-guest binding studies for cage [Zn_4_(**24**_3_,**28**)_4_]^8+^ and helicate [Zn_2_(**24**_2_,**29**)_3_]^4+^_._

20 mL ISynth vials containing pre-weighed quantities of the candidate guests (cyclohexane, o-xylene, tert-butanol, cyclohexanol, cyclooctane, methyl-cyclohexane) were placed inside the Chemspeed. MeCN was dispensed to the vials containing guests and the vials were shaken to produce stock solutions (216 mM). Aliquots (1.2 mL, 60 eq.) of the guest stock solution were dispensed to the replicates previously produced. The vials were shaken for 5 minutes, and aliquots were dispensed into NMR tubes and transported robotically for analysis by NMR-Agent.

## Host-Guest Results

### Host-Guest Binding Studies of Cage [Zn_4_(24_3_,28)_4_](NTf_2_)_8_

| *Guest* | *NMR* |
| --- | --- |
| *tert-Butyl alcohol* | *Pass* |
| *Cyclohexane* | *Pass* |
| *Methylcyclohexane* | *No chemical shift difference* |
| *o-Xylene* | *No chemical shift difference* |
| *Cyclooctane* | *No chemical shift difference* |
| *Cyclohexanol* | *Pass* |

**Table S 52:** Summary of automated decision-maker outcomes for ^1^H NMR spectroscopy.

**Figure S 175:** Partial ^1^H NMR spectra (80 MHz, MeCN) of cage [Zn_4_(**24**_3_,**28**)_4_]^8+^ solutions with guests.

**Figure S 176**: ^1^H NMR spectra (80 MHz, MeCN) of cage [Zn_4_(**24**_3_,**28**)_4_]^8+^ bound with *tert-*butanol (above) and cyclohexane (below).

### Host-Guest Binding Studies of Helicate [Zn_2_(24_2_,29)_3_](NTf_2_)_4_

| *Guest* | *NMR* |
| --- | --- |
| *tert-Butyl alcohol* | *No chemical shift difference* |
| *Cyclohexane* | *No chemical shift difference* |
| *Methylcyclohexane* | *No chemical shift difference* |
| *o-Xylene* | *No chemical shift difference* |
| *Cyclooctane* | *No chemical shift difference* |
| *Cyclohexanol* | *No chemical shift difference* |

**Table S 53:** Summary of automated decision-maker outcomes for ^1^H NMR spectroscopy.

**Figure S 177:** Partial ^1^H NMR spectra (80 MHz, MeCN) of helicate [Zn_2_(**24**_2_,**29**)_3_]^4+^ solutions with guests.

# Offline Photochemical Synthesis

## General Reaction Procedure

Boc-Pro-OH (512 mg, 2.4 mmol, 1.2 eq.) and diethyl benzylidene malonate (0.45 ml, 2 mmol, 1 eq.) were dissolved in DMF (10 mL) and sparged with argon. Separately, vials were charged with K_2_HPO_4_ (42 mg, 0.24 mmol, 1.2 eq.) and the trial photocatalyst (3 mol% loading, or 10 mg for g-CN), and nitrogen flow was enabled to the Chemspeed. The vials and stock solution were placed inside the Chemspeed platform, and the Chemspeed was allowed to purge under nitrogen flow for a further 2 hours. Aliquots of the stock solution (1 mL) were then dispensed to each of the vials, and the vials were crimp sealed using the Chemspeed’s capping machine. The vials were transported robotically and placed inside the photoreactor by UPLC-Agent, and the irradiated overnight (16 h). The vials were then transported back to the Chemspeed, diluted with MeCN (7 mL), and aliquots (2 mL) reformatted into LC-MS vials. The LC-MS vials were then transported by UPLC-Agent for analysis.

Product identity was confirmed with comparison of spectroscopic data to an authentic sample.^12^

## Photocatalysis Results

### General Comments

**Scheme S 45:** Robotic screening of photocatalysts for decarboxylative conjugate addition.

From the catalyst screening experiments, the blank control yielded no product formation, as expected. Graphitic carbon nitride, Eosin Y, and triphenylpyrylium tetrafluoroborate also showed no product formation. 4CzIPN and Ir[dF(CF_3_)ppy]_2_(dtbpy))PF_6_ showed compete conversion of the starting material by LC, while [Ir(dtbbpy)(ppy)_2_]PF_6_ showed partial conversion to the conjugate addition product.

The conjugate addition product appeared as a partially separable mixture of diastereomers on UPLC chromatograms. LCAP for the product is given as an area sum of both diastereomeric peaks. The peak for the conjugate addition product on chromatogram (if any) is marked.

### Photocatalysis UPLC-MS Data

#### Eosin Y

**Figure S 178**: UPLC chromatogram of photocatalyst screening. Photocatalyst: Eosin Y. Conjugate addition products LCAP: 0%.

#### gCN

**Figure S 179**: UPLC chromatogram of photocatalyst screening. Photocatalyst: gCN. Conjugate addition products LCAP: 0%.

#### [Ir(dtbbpy)(ppy)_2_]PF_6_

**Figure S 180**: UPLC chromatogram of photocatalyst screening. Photocatalyst: [Ir(dtbbpy)(ppy)_2_]PF_6_. Conjugate addition products LCAP: 87%.

**Figure S 181**: Mass spectrum of conjugate addition product. Photocatalyst: [Ir(dtbbpy)(ppy)_2_]PF_6_.

**Figure S 182**: Mass spectrum of conjugate addition product. Photocatalyst: [Ir(dtbbpy)(ppy)_2_]PF_6_.

#### 4CzIPN

**Figure S 183**: UPLC chromatogram of photocatalyst screening. Photocatalyst: 4CzIPN. Conjugate addition products LCAP: 100%

**Figure S 184**: Mass spectrum of conjugate addition product. Photocatalyst: 4CzIPN.

**Figure S 185**: Mass spectrum of conjugate addition product. Photocatalyst: 4CzIPN.

#### Ir[dF(CF_3_)ppy]_2_(dtbpy))PF_6_

**Figure S 186**: UPLC chromatogram of photocatalyst screening. Photocatalyst: Ir[dF(CF_3_)ppy]_2_(dtbpy))PF_6_. Conjugate addition products LCAP: 100%.

**Figure S 187**: Mass spectrum of conjugate addition product. Photocatalyst: Ir[dF(CF_3_)ppy]_2_(dtbpy))PF_6_.

**Figure S 188**: Mass spectrum of conjugate addition product. Photocatalyst: Ir[dF(CF_3_)ppy]_2_(dtbpy))PF_6_.

#### Blank Control (no photocatalyst)

**Figure S 189**: UPLC chromatogram of photocatalyst screening. Photocatalyst: None (Blank control). Conjugate addition products LCAP: 0%.

#### TPT-BF_4_

**Figure S 190**: UPLC chromatogram of photocatalyst screening. Photocatalyst: TPT-BF_4_. Conjugate addition products LCAP: 0%

# Workflow Errors, Safety Considerations, and Workflow Autonomy

## Workflow Errors

We had no errors with the mobile robots in any of the three workflows presented here in terms of dropping vials, pick-and-place errors, missed vials, or hardware failures on the UPLC, NMR, Chemspeed, electric actuators or photoreactors over the almost nine combined days of experiment time. We had only two technical errors that required intervention – both involved a WiFi disconnection from UPLC-Agent that required a reconnection to the local network and a restart of the control software. However, this could be done fully remotely without humans needing to be physically present in the lab and required only around 10 minutes of researcher time over the course of the nine days.

## Safety Considerations

While we did not have any incidents with the robot arms dropping racks or vials in our workflows or the development cycle of the workflows, the possibility of this should be taken into careful consideration when implementing mobile robots in a laboratory whenever toxic chemicals are used. While the LIDAR scanners of the mobile base of the robots can detect objects in their path, any small vials and tubes that are dropped by the arm (below where the LIDAR scanners are located) would likely not be detected and could be crushed by the mobile base.

Our main modification to the Chemspeed was the installation of a pair of electric actuators on either side of the Chemspeed to facilitate automated opening and closing of the hood door. There is a particular safety risk to humans in case of the actuators closing the door automatically while humans are present, with the possibility of serious crush injury. To mitigate this risk, we attached a pair of light curtains that cut power to the actuators in the case of anything crossing the path of their infrared beams (Figure S191). We also put an emergency stop button on the front of the Chemspeed, along with alarms that emit warning sounds and flash visibly when the doors are automatically opening and closing. These actuators, however, also prevent access to Chemspeed in case of a power outage unlike an unmodified platform.

We advise researchers to carry out appropriate risk assessments and take necessary steps to ensure the safety of workers in the laboratory, particularly in the context of robots handling of toxic, carcinogenic, and mutagenic chemicals. We also note that while these experiments proved successful with no errors, other than the two WiFi errors mentioned above, we did monitor the reactions carefully throughout for these early-stage proof-of-concept experiments. Moreover, the robotic manipulations and Chemspeed automation were tested extensively over a period of 6 months without any chemicals or with pure water (to simulate sample weights) prior to the introduction of any actual chemistry (see Section 5.3). **We strongly advise a similar progressive approach when introducing new automation techniques into chemistry workflows, whether using mobile robots or otherwise.**

**Figure S 191**: Safety light curtains, warning lights, and emergency stop button on modified Chemspeed.

**Figure S 192**: Actuators for automated door opening on the Chemspeed platform.

## Workflow Autonomy

### Use of a Single Mobile Robot

Initially, two mobile robots were used in the autonomous workflow, each capable of interacting with only one analytical instrument (NMR and LC-MS). The main difference between these mobile robots was the gripper attached to the end of the robot arm. Each gripper was only compatible with its respective, dedicated sample rack (NMR or LC-MS) and there was no cross-compatibility; that is, the NMR robot was only able to pick up NMR racks and could not interact with the LC-MS
racks, and vice-versa.

To integrate the whole workflow within the operational capabilities of a single mobile robot, new LC-MS racks that are compatible with the “NMR gripper” were designed and produced by 3D printing. The gripper of the LC-MS robot was replaced by another 3D-printed “NMR gripper” so the LC-MS robot could carry both NMR racks and the newly designed LC-MS racks. The .STL files needed to 3D print the grippers and the custom NMR and LCMS racks are available as Supplementary Files.

### Human Involvement and Autonomy

Despite advances in robotics and artificial intelligence, any autonomous or automated platform for chemistry still requires human intervention and input. Necessary human involvement can be divided into three main categories: input required for establishing the platform and overall workflow (**setup**), input and interventions required during operation (**in-operation**), and post-operation quality checks (**post-operation**).

***Setup Tasks:*** The extent and nature of setup tasks requiring human intervention affect the ease of starting new projects, but not the platform's autonomy during execution. Human intervention in the setup phase allows for the incorporation of domain-specific expertise, enhancing the trustworthiness of decision-making processes. Different areas of chemistry have different needs; for example, here the diversification chemistry is relatively predictable, whereas the supramolecular chemistry has the propensity to produce a much larger range of products, and often complex product mixtures. Much like autonomous vehicles that require human input to start and set destinations, our platform needs human guidance for initial goal setting and configuration. Also, to carry the analogy, there is a big difference between an autonomous vehicle that navigates a relatively predictable environment, say within an airport, and one that must traverse an open highway. Typically, the setup phase for a new workflow here requires one day of human effort for each of the workflows exemplified here.

***In-operation Tasks:*** The extent and nature of in-operation tasks requiring human intervention directly affect the platform's autonomy during execution, and our aim was to minimize this. Automating most experimental and analytical steps and decision-making processes during operation increases autonomy, provided that the platform’s reliability remains above an acceptable threshold. However, some human intervention is inevitable during operation, primarily for restocking consumables, solvents, and reagents (as automating these processes would be highly cost- and time-inefficient) and performing routine safety checks. Besides restocking and performing safety checks, no human intervention was required during operation, as our automated platform was reliable enough to operate typically without any faults. Restocking and performing safety checks usually required 30 minutes of human intervention each day, which represents an enormous time saving in comparison to manual approaches, both in terms of the physical operations in the synthesis and the analysis, *and* in terms of the time saved in eliminating the need for human data interpretation and decision making.

***Post-operation* *Tasks*:** The extent and nature of post-operation tasks requiring human intervention do not impact the platform's autonomy, as they occur after the workflow has completed. The purpose of this post-operation evaluation is to review the platform's performance and ensure the quality and reliability of the data produced. Like the set-up phase, this also took around one day per workflow.

Due to the hazardous nature of the chemicals involved, as well as the general lack of any extensive prior safety and reliability records for mobile robots in chemistry laboratories – we artificially limited the autonomy of the workflow in these initial experiments. Following the end of the chemical manipulation on the Chemspeed platform, we implemented an artificial pause where a human checks the Chemspeed platform to ensure no failures on the synthesizer – such as spilled chemicals – and sends a confirmation to the scheduler software to permit the robots to open the Chemspeed door and continue with the next stage of the workflow.

While this proved to be not strictly necessary, we implemented it to avoid the possibility of other workers in the lab being accidentally exposed to the chemicals when the door of the Chemspeed is opened automatically. We also intentionally disabled the automatic robot arm background calibration that the mobile robots perform periodically (approximately every 2 hours) to avoid a calibration taking place in an area with low vertical clearance.

We anticipate that as the cumulative safety record for mobile robots in chemistry labs improve, or as future labs are constructed with robots in mind, an even higher degree of freedom and autonomy will be possible than demonstrated in this work here. For example, it might be possible to introduce sensors for volatile organic compounds to replace the manual inspection step outlined above.

The division of tasks between humans and robots/algorithms in our workflow is detailed in Figure S193. Additionally, human input and interventions required during both the setup and operation of our workflow are detailed below.

**Figure S 193**: Division of tasks between humans and robots/algorithms in our workflow.

- **Set-up Phase**
  - Definition of Scientific Targets
    - Selection of the chemistry to be studied and setting of the discovery goals
  - Tuning of the Generic Decision-Maker
    - Adaptation of the HPLC method to the selected chemistry and definition of success criteria
    - Selection of the appropriate NMR spectroscopy experiment and parameters to be used, definition of success criteria
  - Experimental Platform
    - Define Chemspeed operations to achieve the intended chemistry
    - Adapt the Chemspeed physical layout to meet workflow requirements.
    - Load the platform with consumable and reagents:
      - HPLC solvents
      - reagents (weighing reagents beforehand and recording the masses measured)
      - consumables (e.g., reaction vials, NMR tubes, MS vials etc.)
    - Activate all equipment, software and databases required for the platform operation.
    - Pre-run Safety Check of the Platform (e.g. no objects on the floor obstructing the way for the mobile robots, solvents waste empty, etc.)
- **In-operation Phase**
  - Post-run Safety Check of the Platform (e.g. platform seems secured before opening of the Chemspeed door such as no splashing of reagents, solvents waste at adequate level, etc.)
  - Restocking of the platform:
    - Unloading of spent materials and wastes
    - Reloading of the platform with consumable and reagents:
      - HPLC solvents
      - reagents (weighing reagents beforehand and recording the masses measured)
      - consumables (e.g., reaction vials, NMR tubes, MS vials etc.)
  - Pre-run Safety Check of the Platform (e.g. no objects on the floor obstructing the way for the mobile robots, solvents waste at adequate level, etc.)
- **Post-workflow Phase**
  - Evaluation of the platform results
    - This may include off-platform structural elucidation such as high filed NMR, HRMS MS or X-ray crystallography.

Using the definition of “automation” and “autonomy” provided by Jensen *et al.*^13^

*“[..] we define automation herein as the act of making a process occur without human intervention and autonomy as a paradigm where feedback and adaptive decision-making afford the system agency over the manner of its actions.”*

The system that we present is autonomous; it adapts according to feedback and autonomous decision making. Human involvement is limited to the input needed for setting up the platform and overall workflow, affecting only the ease of starting new workflows but not the platform's autonomy during execution.

# Crystallographic data

| **Identification code** | tf-sv3_autored | tf-jfa5_autored |
| --- | --- | --- |
| **Empirical formula** | C_17_H_11_F_6_N_3_O | C_177_H_149_B_4_N_15_Zn_2_ |
| **Formula weight** | 387.29 | 2660.08 |
| **Temperature/K** | 100.03(13) | 100.00(10) |
| **Crystal system** | monoclinic | orthorhombic |
| **Space group** | C2/c | P2_1_2_1_2 |
| **a/Å** | 32.6100(15) | 26.34270(10) |
| **b/Å** | 10.4072(6) | 19.25420(10) |
| **c/Å** | 9.5474(4) | 14.31920(10) |
| **α/°** | 90 | 90 |
| **β/°** | 90.286(4) | 90 |
| **γ/°** | 90 | 90 |
| **Volume/Å^3^** | 3240.1(3) | 7262.81(7) |
| **Z** | 8 | 2 |
| **ρ_calc_g/cm^3^** | 1.588 | 1.216 |
| **μ/mm^‑1^** | 1.32 | 0.863 |
| **F(000)** | 1568 | 2792 |
| **Crystal size/mm^3^** | 0.173 × 0.025 × 0.013 | 0.487 × 0.398 × 0.33 |
| **Radiation** | Cu Kα (λ = 1.54184) | Cu Kα (λ = 1.54184) |
| **2Θ range for data collection/°** | 5.42 to 165.342 | 5.686 to 161.974 |
| **Index ranges** | -41 ≤ h ≤ 41, -13 ≤ k ≤ 13, -12 ≤ l ≤ 10 | -24 ≤ h ≤ 32, -24 ≤ k ≤ 24, -18 ≤ l ≤ 18 |
| **Reflections collected** | 23078 | 150187 |
| **Independent reflections** | 3488 [R_int_ = 0.0825, R_sigma_ = 0.0421] | 15896 [R_int_ = 0.0261, R_sigma_ = 0.0133] |
| **Data/restraints/parameters** | 3488/0/248 | 15896/630/1117 |
| **Goodness-of-fit on F^2^** | 1.139 | 1.044 |
| **Final R indexes [I>=2σ (I)]** | R_1_ = 0.0786, wR_2_ = 0.1739 | R_1_ = 0.0647, wR_2_ = 0.1886 |
| **Final R indexes [all data]** | R_1_ = 0.1101, wR_2_ = 0.1907 | R_1_ = 0.0662, wR_2_ = 0.1911 |
| **Largest diff. peak/hole / e Å^-3^** | 0.36/-0.25 | 0.95/-0.80 |
| **Flack parameter** | - | 0.49(3) |
| **CSD deposition code** | 2355749 | 2355750 |

# Laboratory layout

**Figure S 194**: Physical layout and relative arrangement of mobile agents, chemistry equipment, and the paths taken in the laboratory by the UPLC-Agent and the NMR-Agent. The two mobile robot agents follow set paths between the equipment and return to their own charging stations while the NMR, Chemspeed ISynth, or UPLC are running. On average, the two mobile robot agents are active for just 10% of the total workflow time; as such, there is significant scope to expand the workflow without increasing the number of mobile agents. Total laboratory dimensions = 16x13 m.

**Figure S 195**: **a,** A photograph showing the overview of the laboratory and spatial arrangement of the instruments involved in the workflow. **b,** Photographs of the robot agents transferring sample racks from the ISynth to the NMR (left) and UPLC-MS (right). **c**, Photographs of screening samples being transferred into the SynLED photoreactor for irradiation.

# References

1. Sakai, R. *et al.* Efficient colorimetric anion detection based on positive allosteric system of urea-functionalized poly(phenylacetylene) receptor. *Macromolecules* **43**, 7406–7411 (2010).

2. Fleckenstein, C. A. & Plenio, H. Aqueous/organic cross coupling: Sustainable protocol for Sonogashira reactions of heterocycles. *Green Chem.* **10**, 563 (2008).

3. Mukherjee, C., Mäkinen, K., Savolainen, J. & Leino, R. Chemistry and biology of oligovalent β‐(1→2)‐linked oligomannosides: new insights into carbohydrate‐based adjuvants in immunotherapy. *Chem. – Eur. J.* **19**, 7961–7974 (2013).

4. Aiken, S. G. *et al.* Iterative synthesis of 1,3-polyboronic esters with high stereocontrol and application to the synthesis of bahamaolide A. *Nat. Chem.* **15**, 248–256 (2023).

5. Berrino, E. *et al.* Azidothymidine “clicked” into 1,2,3-triazoles: first report on carbonic anhydrase–telomerase dual-hybrid inhibitors. *J. Med. Chem.* **63**, 7392–7409 (2020).

6. Jiménez, A. *et al.* Selective encapsulation and sequential release of guests within a self-sorting mixture of three tetrahedral cages. *Angew. Chem. Int. Ed.* **53**, 4556–4560 (2014).

7. Zenka, M., Preinl, J., Pertermann, E., Lützen, A. & Tiefenbacher, K. A water‐ and base‐stable iminopyridine‐based cage that can bind larger organic anions. *Eur. J. Inorg. Chem.* **26**, e202300110 (2023).

8. Hauser, A., Vef, A. & Adler, P. Intersystem crossing dynamics in Fe(II) coordination compounds. *J. Chem. Phys.* **95**, 8710–8717 (1991).

9. Seredyuk, M., Gaspar, A. B., Kusz, J., Bednarek, G. & Gütlich, P. Variable-temperature X-ray crystal structure determinations of {Fe[tren(6-Mepy)_3_]}(ClO_4_)_2_ and {Zn[tren(6-Mepy)_3_]}(ClO_4_)_2_ compounds: correlation of the structural data with magnetic and Mössbauer spectroscopy data. *J. Appl. Crystallogr.* **40**, 1135–1145 (2007).

10. Yoshida, N. & Ichikawa, K. Synthesis and structure of a dinuclear zinc(ii) triple helix of an N,N-bis-bidentate Schiff base: new building blocks for the construction of helical structures. *Chem. Commun.* 1091–1092 (1997).

11. Schultz, D. & Nitschke, J. R. Designing multistep transformations using the hammett equation:  imine exchange on a copper(i) template. *J. Am. Chem. Soc.* **128**, 9887–9892 (2006).

12. Vijayakrishnan, S., Ward, J. W. & Cooper, A. I. Discovery of a covalent triazine framework photocatalyst for visible-light-driven chemical synthesis using high-throughput screening. *ACS Catal.* **12**, 10057–10064 (2022).

13. Canty, R. B., Koscher, B. A., McDonald, M. A. & Jensen, K. F. Integrating autonomy into automated research platforms. *Digital Discovery* **2**, 1259–1268 (2023).
